# Discovery of sulfonamide-tethered isatin derivatives as novel anticancer agents and VEGFR-2 inhibitors

**DOI:** 10.1080/14756366.2023.2203389

**Published:** 2023-04-25

**Authors:** Moataz A. Shaldam, Hadia Almahli, Andrea Angeli, Rehab Mustafa Badi, Eman F. Khaleel, Abdelrahman I. Zain-Alabdeen, Zainab M. Elsayed, Eslam B. Elkaeed, Rofaida Salem, Claudiu T. Supuran, Wagdy M. Eldehna, Haytham O. Tawfik

**Affiliations:** a Department of Pharmaceutical Chemistry, Faculty of Pharmacy, Kafrelsheikh University, Kafrelsheikh, Egypt; b Department of Chemistry, University of Cambridge, Cambridge, UK; c Department of NEUROFARBA, Section of Pharmaceutical and Nutraceutical Sciences, University of Florence, Sesto Fiorentino, Italy; d Department of Medical Physiology, College of Medicine, King Khalid University, Abha, Saudi Arabia; e Department of Pharmaceutical Chemistry, Faculty of Pharmacy, Tanta University, Tanta, Egypt; f Scientific Research and Innovation Support Unit, Faculty of Pharmacy, Kafrelsheikh Uinversity, Kafrelsheikh, Egypt; g Department of Pharmaceutical Sciences, College of Pharmacy, AlMaarefa University, Riyadh, Saudi Arabia; h Department of Pharmaceutical Organic Chemistry, Faculty of Pharmacy (Boys), Al-Azhar University, Cairo, Egypt; i School of Biotechnology, Badr University in Cairo, Badr City, Egypt

**Keywords:** Synthesis, antitumor agents, biological activities, VEGFR-2 inhibitors, molecular dynamics

## Abstract

In this work, new isatin-based sulphonamides (**6a-i**, **11a-c**, **12a-c**) were designed and synthesised as potential dual VEGFR-2 and carbonic anhydrase inhibitors with anticancer activities. Firstly, all target isatins were examined for *in vitro* antitumor action on NCI-USA panel (58 tumour cell lines). Then, the most potent derivatives were examined for the potential CA inhibitory action towards the physiologically relevant hCA isoforms I, II, and tumour-linked *h*CA IX isoform, in addition, the VEGFR-2 inhibitory activity was evaluated. The target sulphonamides failed to inhibit the CA isoforms that could be attributable to the steric effect of the neighbouring methoxy group, whereas they displayed potent VEGFR-2 inhibitory effect. Following that, isatins **11b** and **12b** were tested for their influence on the cell cycle disturbance, and towards the apoptotic potential. Finally, detailed molecular modelling analyses, including docking and molecular dynamics, were carried out to assess the binding mode and stability of target isatins.

## Introduction

Cancer, a condition of uncontrolled cell growth, remains the most difficult life-threatening disease to treat, despite advances in understanding of its biochemistry and progression^1^. The rising prevalence of cancer treatment failure derives mainly from antitumor drug resistance in cancer cell, posing new challenges to the healthcare system^2^. Many targets have been identified that are involved in one or more steps in regulating tumour cell growth or death^3^. Combining anticancer drugs is becoming a widely accepted strategy and treatment standard for avoiding drug resistance and treatment failure^4^.

**Figure 1.**
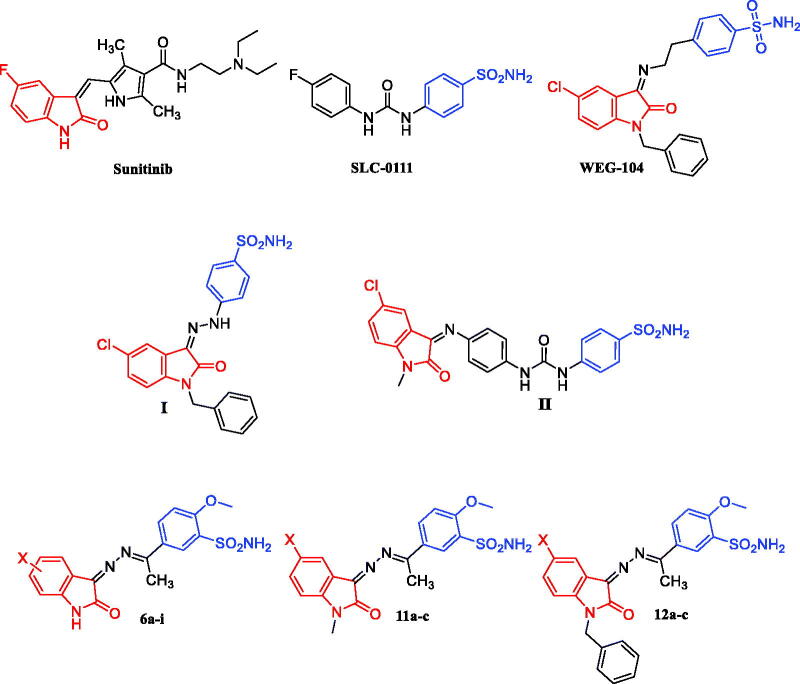
Structure of Sunitinib, SLC-0111, WEG-104, compounds **I-II**, and target compounds **6a-i**, **11a-c** and **12a-c**.

Carbonic anhydrase (CA) is an effector enzyme in the tumour cell survival mechanism that regulates the pH of the tumour microenvironment^5^. CAs are zinc metalloenzymes that catalyse the reversible interconversion of CO_2_ and bicarbonate ions. Several CA isoforms have been identified, with CA IX and CA XII isoforms being upregulated in nearly all hypoxic tumours, promoting tumour growth and metastasis. CA IX isoform overexpression is associated with a poor prognosis in many cancers and is involved in cell proliferation and communication^6^. The primary sulfonamide-based small molecules were discovered to be the most potent chemotype of the identified CA inhibitors (CAIs)^7^. Among those sulfonamide-based inhibitors, **SLC-0111** (Figure 1) displayed effective CA IX and XII inhibitory action, and is currently being examined in the clinical trials for the hypoxic malignancies management^8^.

The clinically validated anticancer drug target, vascular endothelial growth factor receptor-2 (VEGFR-2) is one of the receptor tyrosine kinases that have critical role in vasculogenesis and angiogenesis in many solid cancers. VEGFR-2 mediates the phosphorylation of many proteins in the downstream signalling pathways promoting tumour angiogenesis^9^. Clinical antiangiogenic drugs that are VEGFR-2 inhibitors, such as Sunitinib (Figure 1) showed to achieve normal tumour vasculature and, consequently, contribute in improving chemotherapy treatment. These treatments are planned to hit both antiapoptotic functions and pro-angiogenic activities of VEGF.

Isatin, an endogenic molecule in mammalaian tissues including human, stands for valuable privileged scaffold in drug design and pharmaceutical chemistry^10^. Isatin-tethered compounds have been found to display various pharmacological effects, in particular carbonic anhydrase^11–14^ and VEGFR-2^15–17^ inhibitory activities. In the last few years, isatin motif has been exploited to develop several CAIs with effective *in vitro* and *in vivo* antitumor activities, such as **WEG-104**^18^^,^^19^ and compound **I**^20^ (Figure 1). In 2019, we reported a new set of *N*-substituted isatin derivatives as promising CAIs. Among this set, compound **II** (Figure 1) displayed excellent activity against cancer-related CA IX and XII isoforms; *K*_I_ = 5.2 and 6.3 nM, respectively. Also, it exerted good VEGFR-2 inhibitory action (IC_50_ **=** 260.64 nM), as well as effective cell growth inhibitory action on breast cancer cell lines^21^.

Herein, we decided to develop new isatin-based sulphonamides (**6a-i**, **11a-c** and **12a-c**) as potential dual VEGFR-2 and carbonic anhydrase activities. All target synthesised isatin derivatives **6a-i**, **11a-c** and **12a-c** will be evaluated for *in vitro* antitumor action on NCI-USA panel covering 58 distinct human tumour cell lines. Thereafter, the most potent derivatives will be examined for the potential CA inhibitory action towards physiologically relevant hCA isoforms I, II, and cancer-related hCA IX isoform, in addition, the inhibitory activity against VEGFR-2 will be evaluated.

## Results and discussion

### Chemistry

**Scheme 1.**
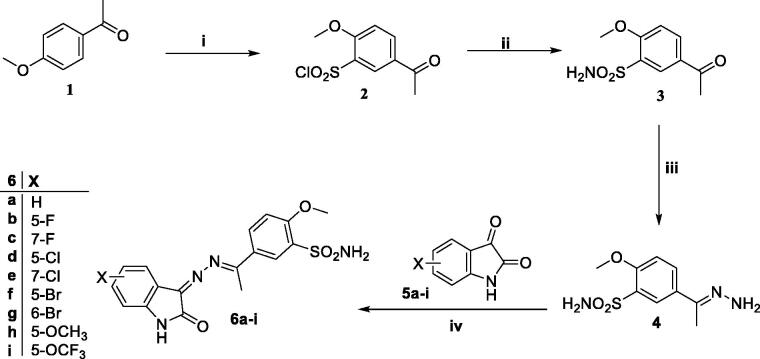
General procedure for the synthesis of target compounds **6a-i**. Reagents and conditions: i) HOSO_2_Cl, SOCl_2_ at 0 °C and then rt, 26 h ii) EtOH, ammonia, rt iii) NH_2_NH_2_.H_2_O, AcOH (cat.), and EtOH, reflux, 4 h. iv) EtOH, AcOH (cat.), reflux, 6–8 h.

General preparation procedures used in synthesising the designed compounds **6a-i** are shown in Scheme 1. The first step in synthesis was the preparation of benzenesulfonyl chloride **2** using thionyl chloride and chlorosulfonic acid in performing chlorosulfonation of compound **1**. An excess chlorinating agent 2:1 was used to enhance the yield after 26 h. The formation of benzenesulfonamide intermediate **3** was accomplished by reacting compound **2** with ammonia using ethanol as solvent^22^. The synthesis of hydrazone intermediate **4** was carried out by refluxing compound **3** with hydrazine hydrate for 4 h in the presence of glacial acetic acid as catalyst and ethanol as solvent^23^. The target compounds **6a-i** were prepared by reacting them with various isatin derivatives **5a-i** in the presence of a catalytic amount of glacial acetic acid and under reflux conditions^24–26^.

**Scheme 2.**
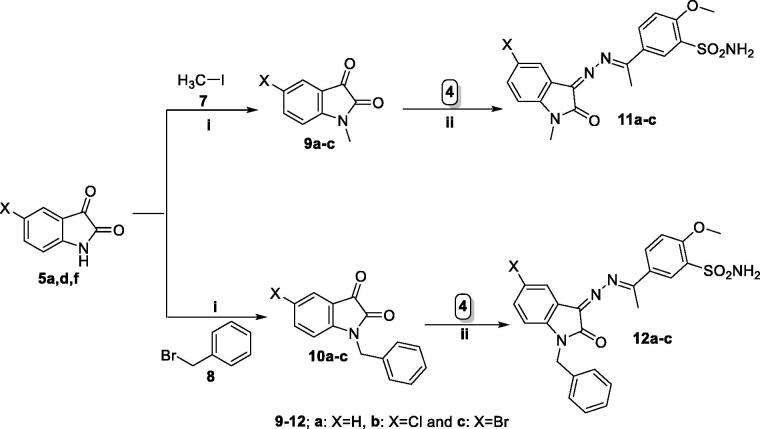
General procedure for the synthesis of target compounds **11a-c** and **12a-c**. Reagents and conditions: **i**) K_2_CO_3_, acetonitrile, reflux, 5 h; **ii**) EtOH, AcOH, reflux, 4 h.

The general procedures for synthesising target *N*-methylated/benzylated isatin derivatives are illustrated in Scheme 2. The *N*-methylation step of isatins **5a**, **5d**, and **5f** was carried out by their reaction with methyl iodide **7** in the presence of potassium carbonate and acetonitrile to afford desired *N*-methylated isatins **9a-c**, which undergo a further condensation reaction with compound **4** in glacial acetic acid to obtain the final compounds **11a-c**. Similarly, benzylated isatin derivatives **10a-c** were synthesised by reacting isatins **5a**, **5d**, and **5f** with benzyl bromide **8** in the presence of acetonitrile and potassium carbonate under refluxing conditions^24^. Thereafter, *N*-benzyl derivatives **10a-c** were condensed with hydrazone intermediate **4** using glacial acetic acid as a catalyst to produce target 2-indolinones **12a-c**.

All the newly prepared molecules were confirmed with ^1^H and ^13^C NMR, mass spectroscopy, and elemental analysis. The protons and carbons signals emerged in the expected chemical shifts (experimental section). According to the ^1^H NMR spectra, the target isatin derivatives exist as E- and Z-isomers, but they interconvert fast in solution at room temperature and cannot be separated. As previously described, the ratio of isomers for isatin hydrazones is solvent and temperature dependent^27–29^, we have reported several studies with such E/Z mixture for different isatin hydrazones^24^^,^^30–32^.

### Biological activity

#### *In vitro* single-dose cellular antiproliferative assay

**Figure 2.**
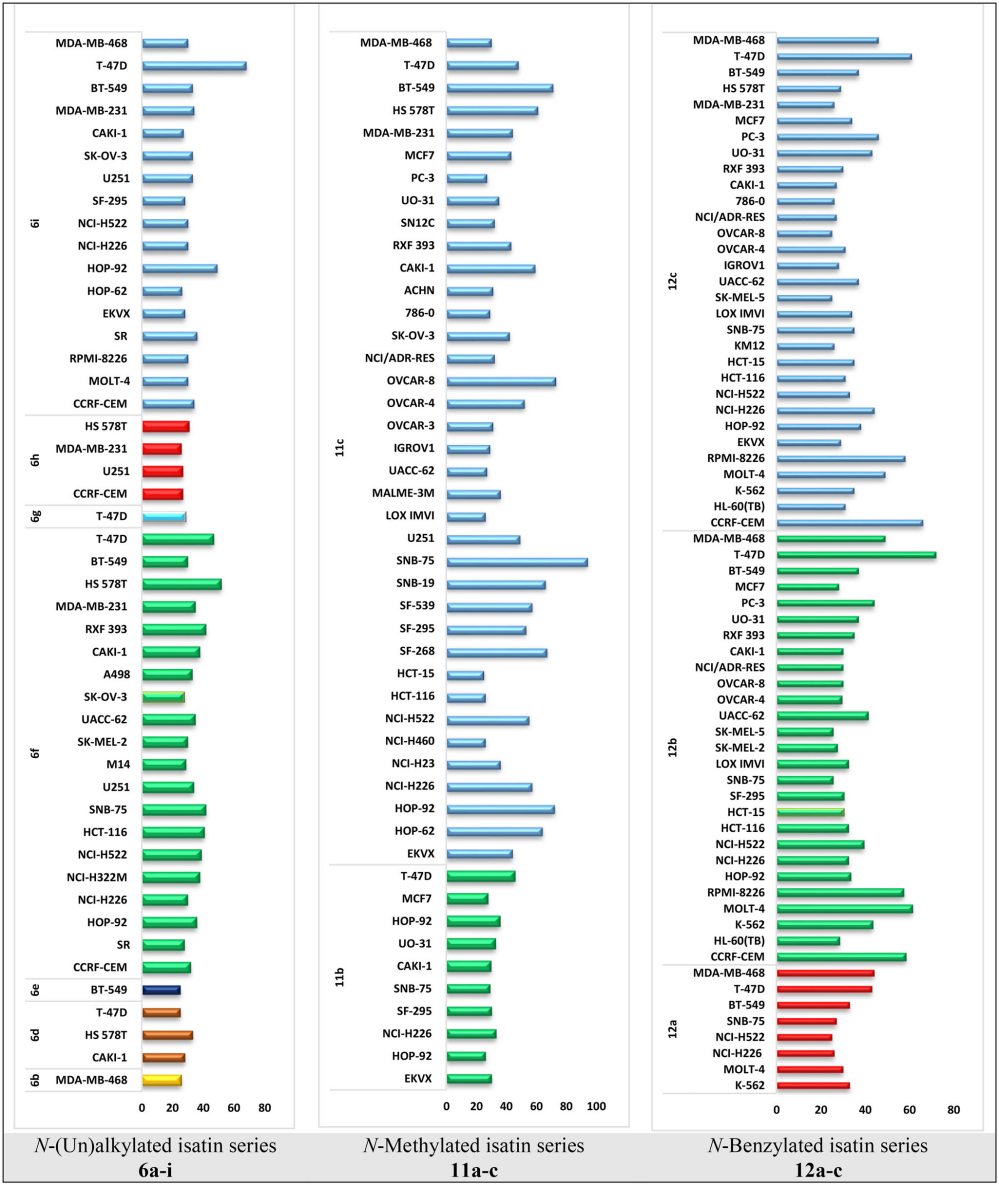
% GI for the highly affected cell lines on treatment by the target isatin derivatives using single dose of 10 μM.

The designed isatin derivatives **6a-i**, **11a-c** and **12a-c** were examined for *in vitro* antitumor activity on NCI panel involving 58 distinct human tumour cell lines covering nine distinct types of cancer, at single concentration of 10 μM (supplementary file). For analysing the activity of the new isatin series on 58-cancer cell line, GI (mean % growth inhibition) displayed by **6a-i**, **11a-c** and **12a-c** on different cell lines were calculated, and results were presented in Figure 2. Based on the findings, we concluded that none of compounds **6a**, **6c**, or **11a** were able to increase cell inhibition by more than 20%. And by looking at the mean growth inhibition of the other compounds on each cancer, we discovered that, in addition to CNS cancer in compound **11c** and leukaemia in compounds **12b** and **12c**, all the compounds had the strongest mean growth inhibition on breast cancer cell lines. We also found that the most sensitive cells in breast cancer subpanel is the T47D cells, and it responded best GI% to compounds **6f**, **6i**, **11b**, **11c**, **12a**, **12b**, and **12c** with GI%; 54, 32, 55, 52, 57, 28 and 39, respectively (Figure 2).

#### *In vitro* cytotoxicity against T47D

For further exploration of possible anti-proliferative properties of isatin derivatives, dose-response evaluation was carried out on the T47D breast cancer cells (the most sensitive cancer cell line according to % GI *in vitro* single-dose cellular antiproliferative assay), employing the sulforhodamine B colorimetric test. Doxorubicin was set as reference in this assay. IC_50_ values were ascertained and are displayed in Table 1. Derivatives **6f, 11b-c,** and **12b** revealed good cytotoxic action demonstrating IC_50_ from 1.83 to 10.40 µM in comparison to doxorubicin (IC_50_ of 2.26 µM). As earlier noted in single-dose assay, a bromo-isatin analogue of C-5-functionalized isatins was preferred in the *N*-(un)alkylated derivatives of isatin series. Compound **6f** (IC_50_ = 5.45 ± 0.24 µM) was found to be the most potent among the studied series. Regarding the *N*-alkylated/arylated analogs of isatin series with methyl or benzyl moiety, the presence of a chloro substitution at position 5 led to the best potency (*N*-methyl analogue **11b**, IC_50_ of 1.83 ± 0.08 µM and *N*-benzyl analogue **12b**, IC_50_ of 3.59 ± 0.16 µM), followed by the C-5-bromo substituted derivatives (compounds **11c** and **12c**, IC_50_ of 10.40 ± 0.47 and 16.52 ± 0.74 µM respectively) as shown in Table 1.

**Table 1.** Cytotoxicity of selected compounds **6f**, **6i**, **11b-c**, and **12a-c** (IC_50_) against T47D cells.

Compound	IC_50_ ± S.D. (µM) T47D^a^
**6f**	5.45 ± 0.24
**6i**	24.13 ± 1.08
**11b**	1.83 ± 0.08
**11c**	10.40 ± 0.47
**12a**	11.58 ± 0.52
**12b**	3.59 ± 0.16
**12c**	16.52 ± 0.74
Doxorubicin^33^	2.26 ± 0.10

^a^IC_50_ values are mean ± SD of triplicate experiments.

#### *In vitro* VEGFR-2 and carbonic anhydrase inhibition activity

Isatins **6f**, **11b-c**, and **12b**, which revealed the most cytotoxic activity on the T47D cells that characterised by the overexpression of VEGFR-2 and carbonic anhydrase^34–36^, were selected for further analysis of their *in vitro* VEGFR-2 and CA inhibition properties (Table 2).

**Table 2.** IC_50_ values of selected compounds **6f**, **11b-c**, and **12b** against VEGFR-2 and CA inhibition activity.

Compound	VEGFR-2^a^		Carbonic anhydrase activity K_I_ (µM)^b^		
	% Inhibition at 10 µM	IC_50_ ± S.D. (nM)	CA I	CA II	CA IX
**6f**	86.82	56.70 ± 0.72^a^	>100	>100	>100
**11b**	72.67	63.40 ± 0.72^a^	>100	>100	>100
**11c**	92.03	30.10 ± 0.31^a^	>100	>100	>100
**12b**	97.18	23.10 ± 0.41^a^	>100	>100	>100
Sorafenib	96.40	29.70 ± 0.17^a^	–	–	–
Acetazolamide (AAZ)	–	–	0.25	0.012	0.026

^a^IC_50_ value is mean ± SD of triplicate experiments, ^b^Mean of triplicate different assays, *via* stopped flow assay.

All derivatives under test demonstrated good VEGFR-2 inhibition with an IC_50_ of 23.10 to 63.40 nM, with compound **12b** being the most potent (IC_50_ = 23.10 nM) and compound **11c** being equivalent to the commonly used kinase inhibitor sorafenib (IC_50_ = 30.10 nM), which had an IC_50_ value of 29.70 nM and the remaining two being marginally less potent.

On the other hand, all the evaluated isatin derivatives failed to inhibit the CA isoforms (*K*_I_ > 100 µM), contrary to predictions, that could be attributable to the steric hindrance by the neighbouring methoxy group^37^.

#### Cell cycle distribution analysis

**Figure 3.**
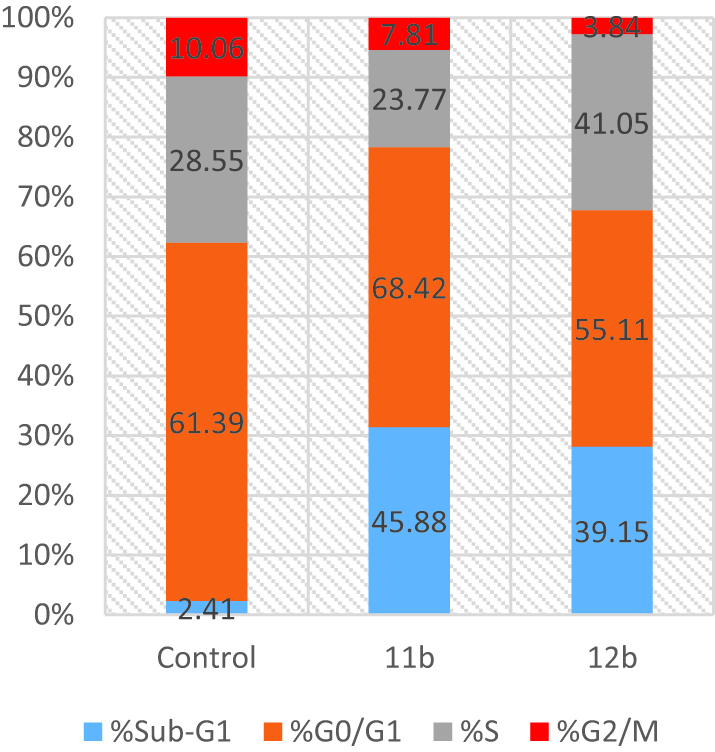
Phases distribution of T47D cells upon incubation with **11b** and **12b**.

To learn more about their cellular mechanisms of action, the most potent compounds (**11b** and **12b**) were selected. DMSO (control) or compounds **11b** and **12b** were applied to T47D cells, and flow cytometry was used to determine the DNA concentration. Figure 3 presents the findings. Both compounds showed different cell-cycle arrest patterns.

Cells treated with **11b** revealed an increase in sub-G1 and G0/G1 phases (45.88% and 68.42%, respectively), compared to 61.39% and 2.41%, respectively, in control. Additionally, compound **12b** increased the proportion of cells in the S and sub-G1 phases of the cell cycle from 28.55% and 2.41% for untreated T47D cells to 41.05% and 39.15%, respectively (Figure 3).

#### Apoptosis assay

**Figure 4.**
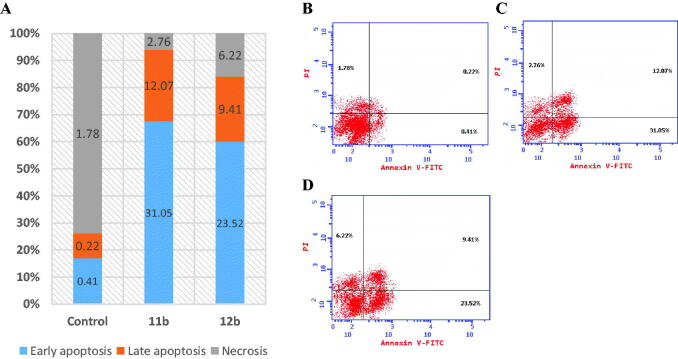
Apoptosis rate quantification (%) and necrosis in (**A**) flow cytometry, effect of (**B**) control, (**C**) **11b** and (**D**) **12b** on annexin V-FITC-positive staining (%) in T47D cell line.

To explore link between growth suppression activity of compounds **11b** and **12b** with initiation of apoptosis indicated by rising population of sub-G1 in treated T47D cell lines, the Annexin V-FITC/PI double labelling (AV/PI) apoptosis assay was done. The outcomes of the experiment are shown in Figure 4, showing that compounds **11b** and **12b** caused T47D cell lines to undergo early and late apoptosis. In fact, % of apoptotic cells amplified from 0.41% at early and 0.22% at late apoptosis in T47D cells that had not been treated to 31.0% of early and 12.07% of late apoptosis in T47D cell that had been treated with compound **11b** and to 23.5% at early and 9.4% at late apoptosis of T47D cells that had been treated with compound **12b**. According to these findings, compounds **11b** and **12b** increased the overall apoptosis of T47D cells by roughly 19 and 16.2 times, respectively, in comparison to the control.

### *In silico* studies

#### Molecular docking

The VEGFR-2 crystal structure (code 4ASD) was used in the molecular docking investigation that was done for predicting binding mode of **12b** as most active compound presented in this study. As predicted, **12b** fits deeply into the active site region with nearly the same orientation as the co-crystallized ligand (sorafenib, Figure S64).

**Figure 5.**
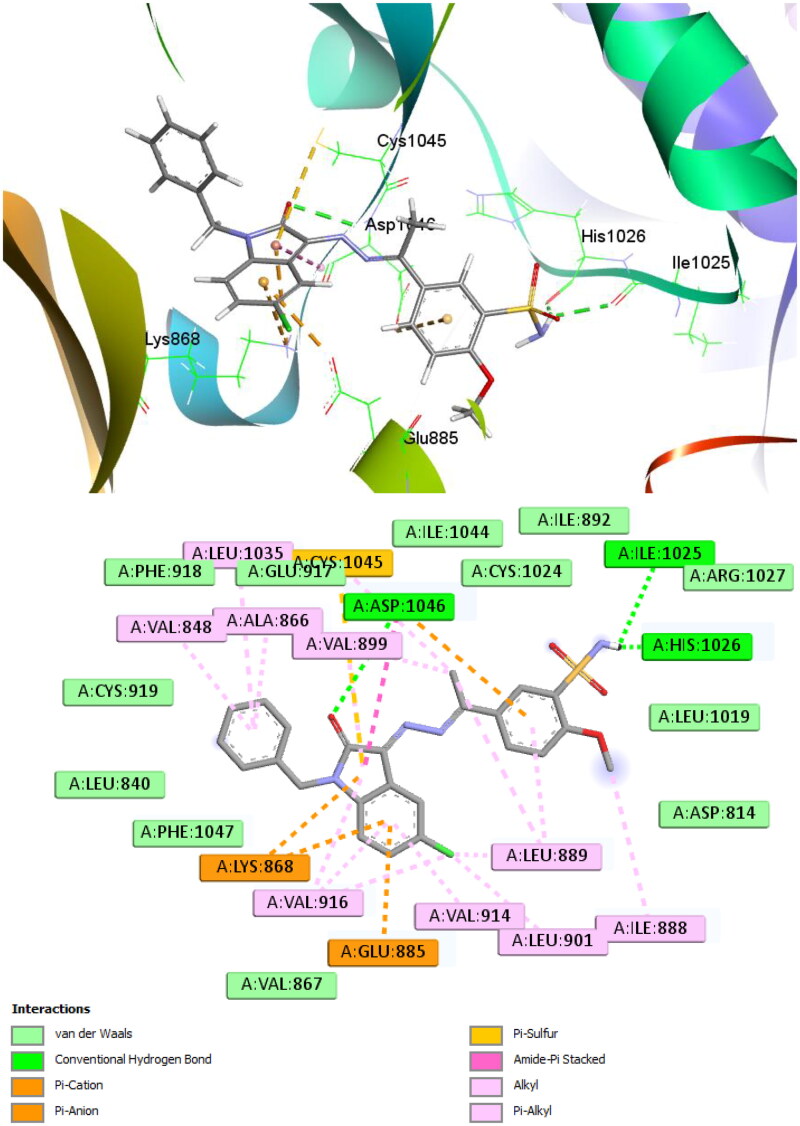
Docking of **12b** inside the active site of VEGFR-2 receptor (code: 4ASD); active site view (top) and 2D schematic view of the interactions (bottom).

In addition, the H-bonds were formed by the sulfamoyl amino group with Ile1025 and His1026 and between the isatin carbonyl group with Asp1046 (Figure 5). The heterocyclic rings of **12b w**ere involved in π-ion interactions with Asp1046, Lys868 and Glu885 while Cys1045 interacted with the five membered ring of isatin via π-sulfur interaction. Additionally, the benzyl ring and other hydrophobic components of **12b** were shown to interact with several hydrophobic residues found in the active site of the VEGFR-2 receptor, including Val848, Ala866, Leu889, Val899, Leu901, Val914, Val916, and Leu1035. All the aforementioned interactions were responsible for the good affinity of **12b** to the studied receptor as indicated by the docking score of −8.5 kcal/mol.

#### Molecular dynamic simulation

**Figure 6.**
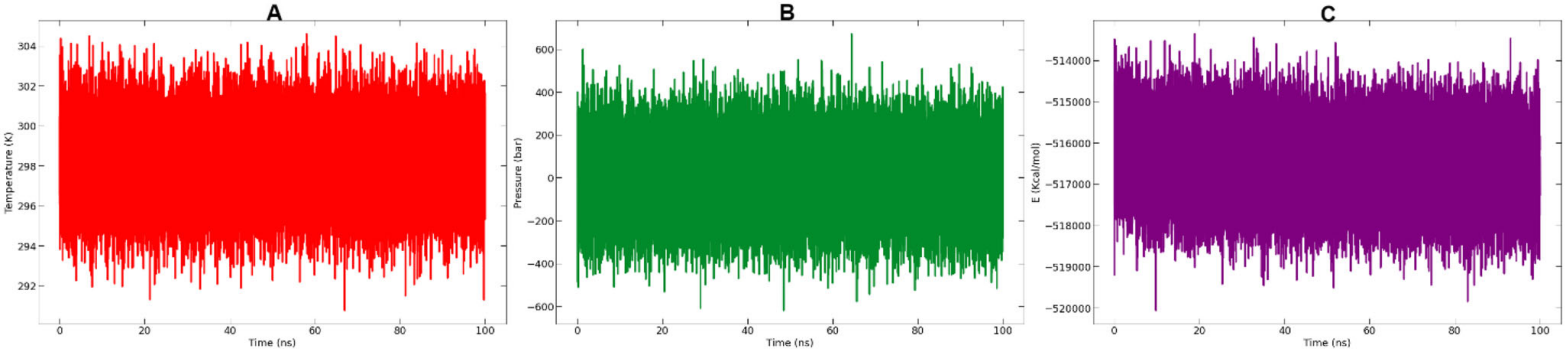
From left to right: **(A)** Temperature, **(B)** pressure and **(C)** potential energy during the 100 ns MD simulations.

After the docking process of **12b** into the VEGFR-2 receptor, the stability of the complex structure needed to be examined. To test the durability of the best pose for the **12b**-VEGFR-2 complex at room temperature circumstances, a 100 ns MD simulation was used. Over this simulation period, the complex system’s temperature, pressure and potential energy, are shown in Figure 6 showing converged system throughout whole simulation period.

**Figure 7.**
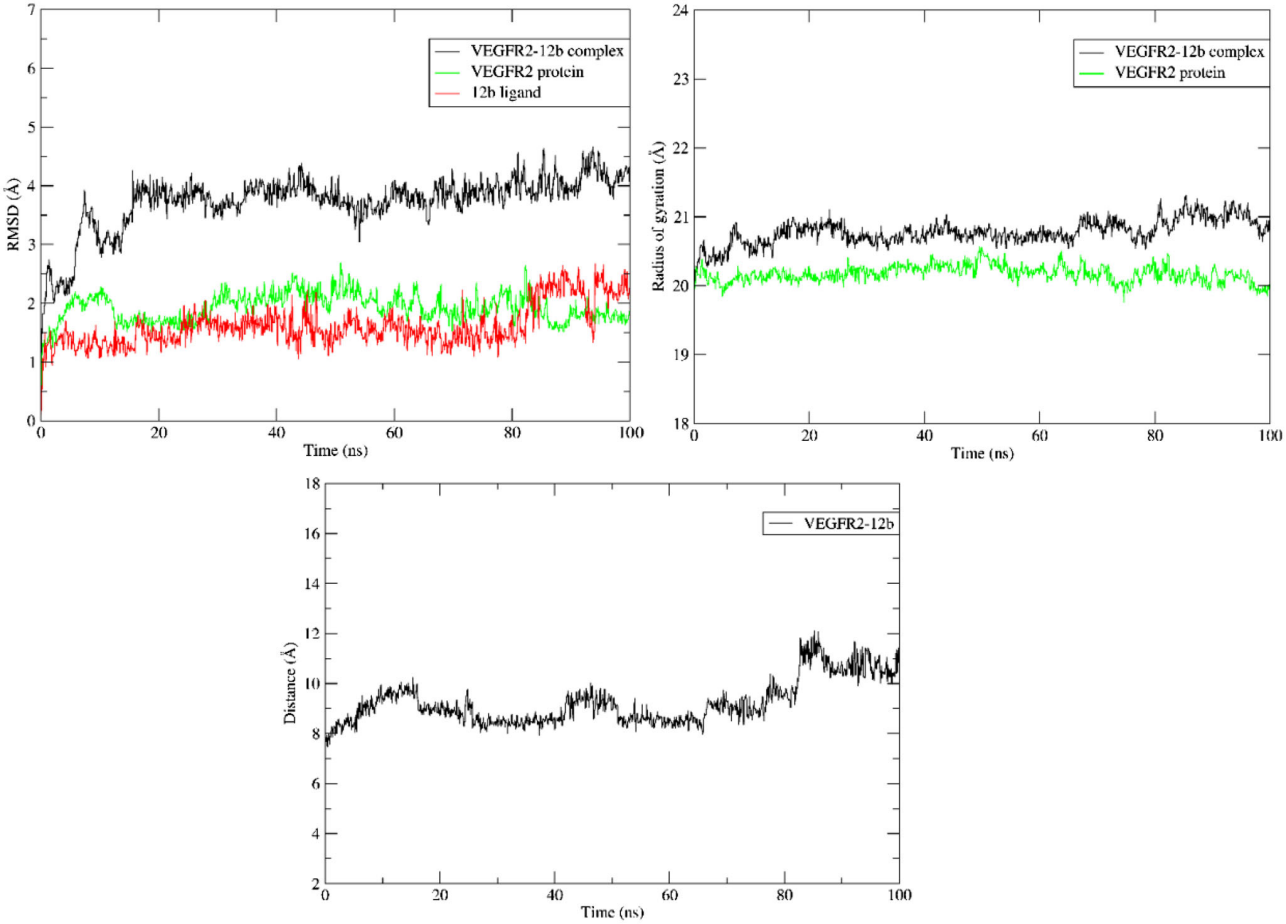
RMSD, Radius of gyration and average centre of mass distance of heavy atoms of **12b** during 100 ns MD simulation.

Analysing trajectories after simulation showing **12b** ligands stayed attached to active site in protein pocket as indicated by the calculated RMSD, radius of gyration and average of distance at mass centre between ligand and protein Figure 7.

**Figure 8.**
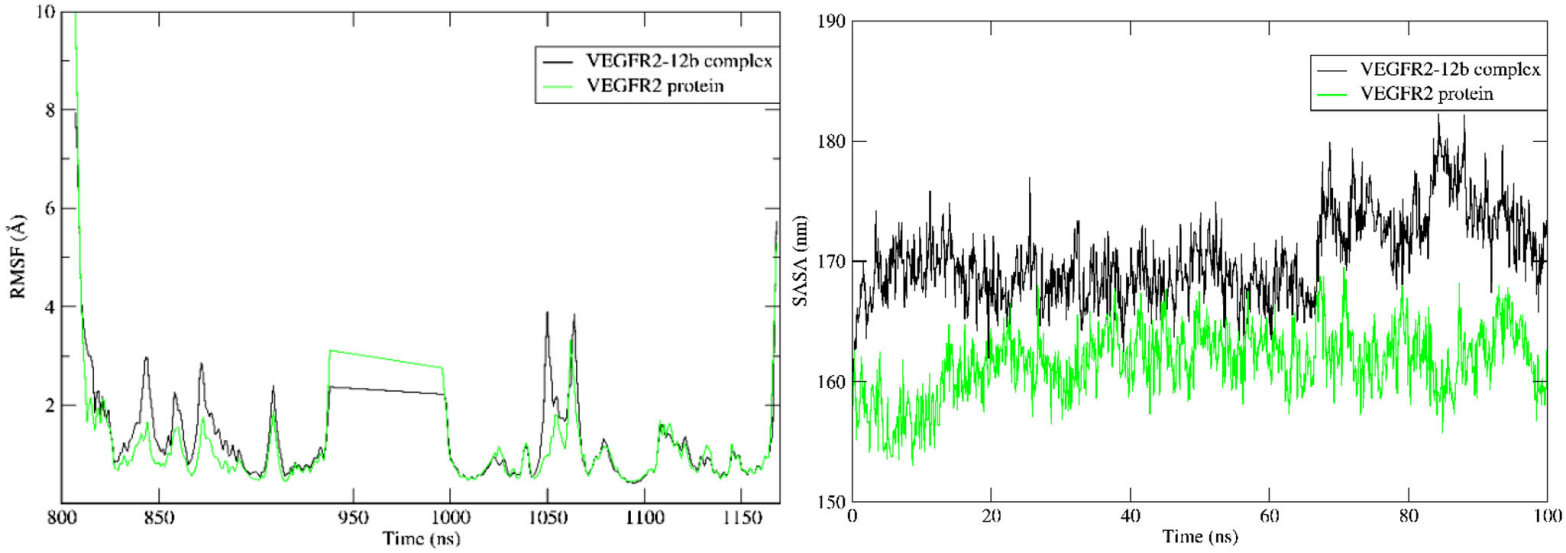
RMSF and SASA of VEGFR-2 in presence and absence of **12b** over 100 ns MD simulation.

Furthermore, SASA and RMSF of protein over the simulation period showed no significate change in absence and presence of **12b** as shown in Figure 8.

**Figure 9.**
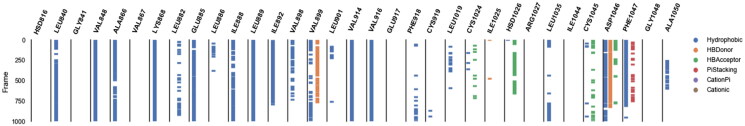
Different types of interaction exhibited by **12b** with the amino acids within the active site of VEGFR-2 during the whole MD simulation frames.

**Figure 10.**
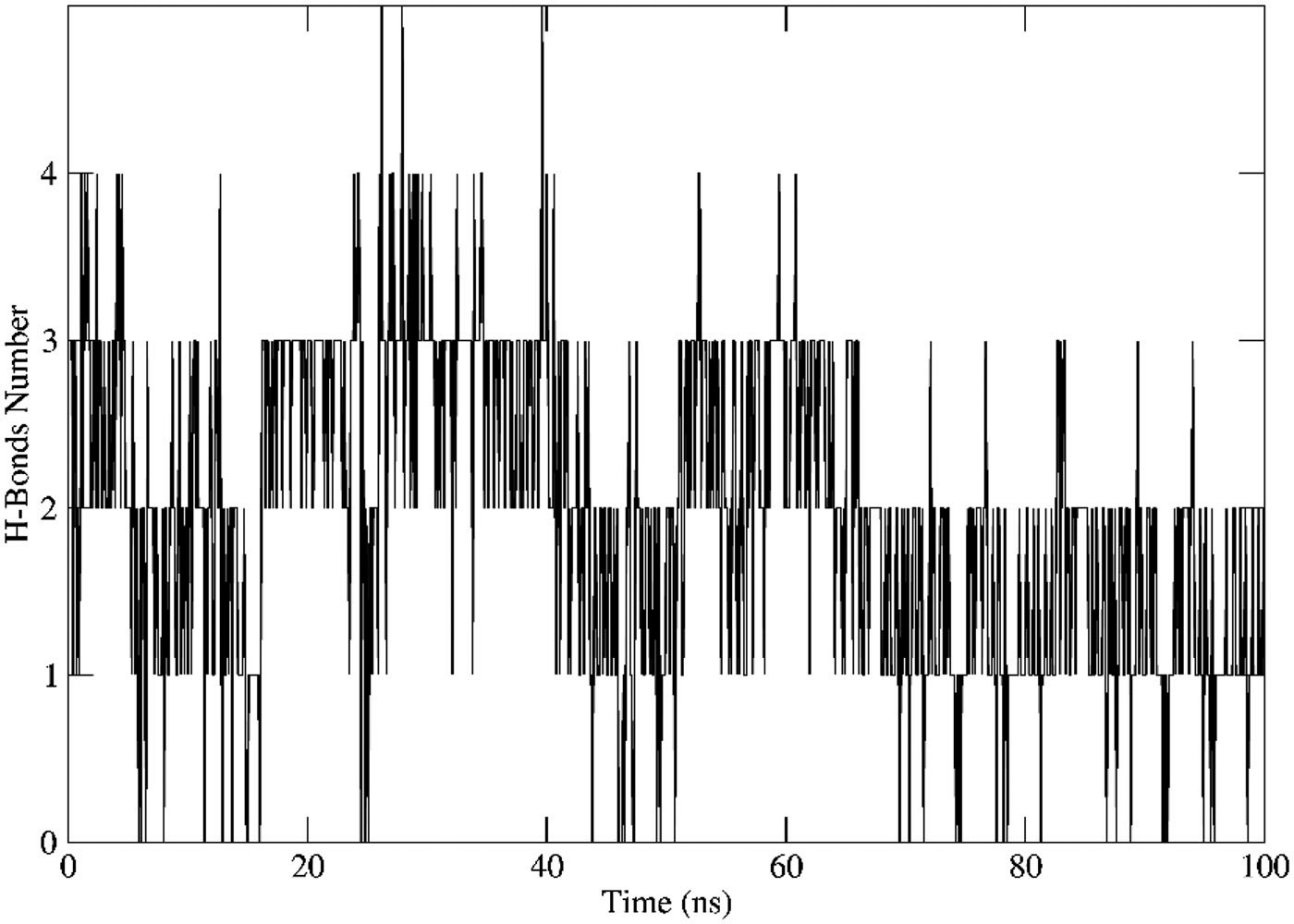
Hydrogen bonds between VEGFR-2 and **12b** during 100 ns MD simulation.

Evaluating interactions of **12b** to the nearby residues within the active site of VEGFR-2 showed various stable interactions over the simulation period (Figure 9). Different forces contributing to the affinity of **12b** to the receptor were observed during 100 ns of the simulation run including π-stacking, H-bonding and hydrophobic, Figure S65–67. Figure 10 depicts the total number of H-bonds formed by the protein and ligand during the simulation period. On average, there were two to three H-bonds to the VEGFR-2 receptor remained stable throughout the entire simulation. Finally, all these interactions were reflected in the binding free energy (MMPBSA) that were carried out to assess the stability of the formed complex structure (Table 3).

**Table 3.** Free binding energies of **12b** with VEGFR-2 in kJ/mol.

Binding energies	kJ/mol
Electrostatic energy	−84.723 ± 46.032
Polar solvation energy	189.645 ± 48.831
SASA energy	−24.848 ± 1.635
van der Waal energy	−195.211 ± 25.865
ΔG	−115.137 ± 28.682

## Conclusions

In the current study, different sets of isatin-based sulphonamides (**6a-i**, **11a-c** and **12a-c**) were reported with the prime goal of developing new anticancer candidates with dual VEGFR-2 and carbonic anhydrase inhibitory actions. *In vitro* anticancer activities of target isatins were first explored towards 58 tumour cell lines (NCI-USA panel). The findings revealed that the breast cancer subpanel was the most susceptible to the influence of target isatins. In particular, the T47D cells growth was effectively inhibited by compounds **6f**, **6i**, **11b-c** and **12a-c**. Then, the IC_50_ values of these isatins towards T47D cells were determined (IC_50_ range: 1.83 − 24.13 µM). Thereafter, the VEGFR-2 and carbonic anhydrase inhibitory actions of isatins **6f**, **11b-c**, and **12b** were evaluated. While the target isatin sulphonamides potently inhibited VEGFR-2 (IC_50_ range: 23.10 − 63.40 nM), they failed to inhibit the CA isoforms (*K*_I_ > 100 µM), contrary to predictions, that could be attributable to the steric hindrance by the neighbouring methoxy group. Moreover, isatins **11b** and **12b** were tested for their effect on cell cycle, and towards apoptotic potential.

## Experimental

### Chemistry

#### General

Reactions progress was checked by TLC sheets (Silica 60 F_254_, Fastman Kodak Co.) using methanol and chloroform: (10: 90) as elution system: and visualisation was done at 254 nm. Melting point was measured on Stuart SMP10 using the open capillary technique and was reported uncorrected. ^13^C NMR (101–126 MHz) and ^1^H (400–500 MHz) spectra were obtained using Bruker FTNMR spectrometer using DMSO-*d*_6_ as a solvent. Spectra of high-resolution mass were recorded on Bruker MicroTOF spectrometer. Elemental analysis (% C, H, N, and S) was done using CHNS analyser (2400 Perkin–Elmer). The spectral data and their interpretation were deposited in the supplementary file.

#### General steps for preparation of 5-acetyl-2-methoxybenzene-1-sulfonyl chloride (2)

Chlorosulfonic acid (90 mmol) was stirred with thionyl chloride (30 mmol) in an ice bath for 30 min. After that, 4-methoxyacetophenone **1** (15 mmol) was added to the mixture dropwise, followed by stirring the reaction mixture for 26 h at room temperature. Subsequently, ice water was added, and the resulting pale orange precipitate **2** was taken after being filtered and washed using H_2_O (3.35 g, 90%), Mp: 102–103 °C^22^^,^^37^.

#### General steps for preparation of 5-acetyl-2-methoxybenzenesulfonamide (3)

Compound **2** (10 mmol) was stirred in ethanol (30 ml) as solvent at room temperature while 30 ml ammonia was added to the mixture. Completion of the reaction detected *via* TLC. The pink precipitated compound **3** was isolated and purified using ethanol and water (1.81 g, 79%), Mp: 202–203 °C^22^^,^^38^.

#### General procedure for preparation of 5-[(1E)-ethanehydrazonoyl]-2-methoxybenzene-1-sulfonamide (4)

Compound **3** (7.9 mmol) was refluxed with hydrazine hydrate (10 mmol), glacial acetic acid (2.5 ml) and ethanol (50 ml) for 4 h. Next, the mixture brought to room temperature, then its extraction with DCM (50 ml × 3). The obtained product **4** was dried over Na_2_SO_4_, concentrated under vacuum, and used in the next step without further purification.

#### General steps for preparation of 2-methoxy-6-[1–(2-[2-oxo-2,3-dihydro-1H-indol-3-ylidene]hydrazin-1-ylidene)ethyl]benzene-1-sulfonamide derivatives (6a-i)

Compound **4** (0.4 mmol) was stirred with different derivatives of isatin **5a-i** (0.4 mmol) using absolute ethanol as solvent under reflux conditions for 6–8 h with the addition of glacial acetic acid using catalytic amount. The formed precipitate was filtered and washed with ethanol before recrystallizing it from DMF to afford desired compounds **6a-i**.

#### General steps for preparation of N-methylated derivatives of 2-methoxy-6-[(1E)-1–(2-[(3E)-2-oxo-2,3-dihydro-1H-indol-3-ylidene]hydrazin-1-ylidene)ethyl]benzene-1-sulphonamides (11a-c)

Isatin derivatives **5a**, **5d** and **5f** (2 mmol) were refluxed with (2.8 mmol) of methyl iodide **7** in acetonitrile (20 ml) using a catalytic amount of potassium iodide and dry potassium carbonate (10 mmol). TLC was used in monitoring the reaction progression. After reaction completion, it was added over ice water; the resulting solid was collected, washed with water, and recrystallized from ethanol and water to produce the intermediate compounds **9a–c**. Subsequently, isatin derivatives **9a-c** reacted with compound **4** in the same conditions described previously in the preparation of compounds **6a-i**.

#### General procedure for preparation of N-benzylated derivatives of 2-methoxy-6-[(1E)-1–(2-[(3E)-2-oxo-2,3-dihydro-1H-indol-3-ylidene]hydrazin-1-ylidene)ethyl]benzene-1-sulphonamides (12a-c)

Isatin derivatives **5a**, **5d** and **5f** (2 mmol) were refluxed with (2.8 mmol) of benzyl bromide **8** in acetonitrile (20 ml) using a catalytic amount of potassium iodide and dry potassium carbonate (4 mmol). TLC was used in monitoring the reaction progression. After reaction completion, it was added over ice water; the resulting solid was collected, washed with water, and recrystallized from ethanol and water to produce the intermediate compounds **10a-c**. Subsequently, isatin derivatives **10a-c** reacted with compound **4** in the same conditions described previously in the synthesis of compounds **6a-i**.

### Biological evaluation

The NCI-USA antitumor assessment^39^, MTT viability^40^^,^^41^, VEGFR-2 inhibition^42^^,^^43^, CA I, II, and IX inhibition studies^44–47^, cell cycle analysis and annexin V-FITC apoptosis assay^48^^,^^49^ were all carried out as previously published in the current study. The supplementary data included descriptions of every experimental technique.

### Molecular modeling

#### Molecular docking studies

The crystal structure of VEGFR-2 (Code: 4ASD)^50^ was retrieved from protein data bank. The docking study was carried out on the co-crystalized ligand (sorafenib) and **12b** as a promising inhibitor. Ligands were drawn into Marvin Sketch V19.12^51^. The docking was performed using AutoDock Vina^52^ in accordance with our previous report^5^. The active site was defined by the grid box of (x= −23.3, y = 0.1 and z= −10.1) coordinates with size (x = 23.2, y = 18.3, z = 21.6). Using the Discovery studio client, the 3D visualisation and the 2D schematic presentation were produced^53^.

#### MD Simulation

The best docking pose was subjected for molecular dynamics (MD) using GROMACS 2021 over 100 ns^54^. The details of applied parameters and procedures were mentioned in the supplementary file.

## Supplementary Material

Supplemental Material
