# Supplementary material for: Discovery of sulfonamide-tethered isatin derivatives as novel anticancer agents and VEGFR-2 inhibitors
Source: J Enzyme Inhib Med Chem. 2023 Apr 25;38(1):2203389. doi: 10.1080/14756366.2023.2203389 (PMC10134960; doi:10.1080/14756366.2023.2203389)
Supplement: Supplemental Material [file IENZ_A_2203389_SM2212.pdf]

Supplementary Material

Discovery of sulfonamide-tethered isatin derivatives as novel anticancer agents and VEGFR-2 inhibitors

Moataz A. Shaldam[a], Hadia Almahli[b], Andrea Angeli[c], Rehab Mustafa Badi[d], Eman F. Khaleel[d], Abdelrahman I. Zain-Alabdeen[e], Zainab M. Elsayed[f], Eslam B. Elkaeed[g,h], Rofaida Salem[a], Claudiu T. Supuran[c,*], Wagdy M. Eldehna[a,i,*], Haytham O. Tawfik[e,*]

[a] *Department of Pharmaceutical Chemistry, Faculty of Pharmacy, Kafrelsheikh University, Kafrelsheikh, P.O. Box 33516, Egypt*
[b] *Department of Chemistry, University of Cambridge, Cambridge, CB2 1EW, United Kingdom*
[c] *Department of NEUROFARBA, Section of Pharmaceutical and Nutraceutical Sciences, University of Florence, Polo Scientifico, Via U. Schiff 6, 50019, Sesto Fiorentino, Firenze, Italy*
[d] *Department of Medical Physiology, College of Medicine, King Khalid University, Abha, Saudi Arabia*
[e] *Department of Pharmaceutical Chemistry, Faculty of Pharmacy, Tanta University, Tanta 31527, Egypt*
[f] *Scientific Research and Innovation Support Unit, Faculty of Pharmacy, Kafrelsheikh Uinversity, Kafrelsheikh, Egypt*
[g] *Department of Pharmaceutical Sciences, College of Pharmacy, AlMaarefa University, Riyadh 13713, Saudi Arabia*
[h] *Department of Pharmaceutical Organic Chemistry, Faculty of Pharmacy (Boys), Al-Azhar University, Cairo 11884, Egypt*
[i] *School of Biotechnology, Badr University in Cairo, Badr City 11829, Egypt*

*Corresponding author: Claudiu T. Supuran; claudiu.supuran@unifi.it, Wagdy M. Eldehna; wagdy2000@gmail.com, Haytham O. Tawfik haytham.omar.mahmoud@pharm.tanta.edu.eg

List of content

Chemistry data	S3
Characterization and spectral analyses of compounds **6a-i, 11a-c,** and **12a-c**	S10
Elemental Analyses	S34
NCI *in vitro* single-dose cellular antiproliferative assay results	S35
Biological Evaluation	S53
MD simulation	S55
References	S56

Chemistry data

5-[(1*E*)-ethanehydrazonoyl]-2-methoxybenzene-1-sulfonamide (4)

Yield (75%) as a white powder with m.p 218-220°C. ^{1}H NMR (500 MHz, DMSO-d_6) δ (*ppm*): 1.99 (3H, s, CH_3), 3.88 (3H, s, CH_3), 6.36 (2H, s, Ar-H), 7.06 (2H, s, SO_2NH_2), 7.13 (1H, d, *J*= 8.0 Hz, Ar-H), 7.72-7.74 (1H, m, Ar-H), 8.05 (1H, s, Ar-H). ^{13}C NMR (125 MHz, DMSO) δ (ppm): 11.24, 56.15, 112.26, 123.93, 129.57, 131.05, 131.78, 140.74, 155.02. Anal. Calcd. For $C_9H_{13}N_3O_3S$: C, 41.15; H, 4.22; N, 10.66; S, 12.20. Found: C, 40.90; H, 4.16; N, 10.49; S, 12.14.

2-Methoxy-6-[(1*E*)-1-(2-[(3*E*)-2-oxo-2,3-dihydro-1*H*-indol-3-ylidene]hydrazin-1-ylidene)ethyl] benzene-1-sulfonamide (6a)

Yield (75%) as a white powder, *E*:*Z* ratio= 55:45, with m.p 250-252°C. ^{1}H NMR (400 MHz, DMSO-d_6) δ (*ppm*): 2.28, 2.31 (2s, 3H, CH_3), 3.95, 3.98 (2s, 3H, OCH_3), 6.88-6.96 (m, 1.45H, Ar-H), 7.16, 7.24 (2s, 2H, SO_2NH_2), 7.27 (d, 1.65H, *J*= 8.0 Hz, Ar-H), 7.34-7.38 (m, 1.35H, Ar-H), 7.49 (d, 0.55H, *J*= 8.0 Hz, Ar-H), 8.06 (dd, 0.55H, *J*= 8.0, 2.4 Hz, Ar-H), 8.16 (dd, 0.45H, *J*= 8.0, 2.4 Hz, Ar-H), 8.33 (d, 0.55H, *J*= 2.4 Hz, Ar-H), 8.39 (d, 0.45H, *J*= 2.4 Hz, Ar-H), 10.84 (s, 1H, NH of isatin). ^{13}C NMR (100 MHz, DMSO) δ (ppm): 14.97, 15.46, 49.05, 56.85, 56.98, 111.28, 113.02, 113.46, 116.84, 122.69, 126.68, 127.92, 128.60, 130.02, 131.80, 132.08, 132.34, 132.91, 133.83, 145.18, 146.55, 157.46, 157.64, 158.03, 158.29, 164.65. EI-MS: *m/z*: 391.088 $[M+H]^+$. Anal. Calcd. For $C_{17}H_{16}N_4O_4S$: C, 52.30; H, 3.87; N, 14.35; S, 8.21. Found: C, 52.11; H, 3.95; N, 14.50; S, 8.18.

5-[(1*E*)-1-(2-[(3*E*)-5-Fluoro-2-oxo-2,3-dihydro-1*H*-indol-3-ylidene]hydrazin-1-ylidene)ethyl]-2-methoxybenzene-1-sulfonamide (6b)

Yield (71%) as a red powder, *E*:*Z* ratio= 70:30, with m.p 262-264°C. ^{1}H NMR (400 MHz, DMSO-d_6) δ (ppm): 2.31, 2.37 (2s, 3H, CH_3), 3.98, 4.01 (2s, 3H, OCH_3), 6.90-6.93 (m, 0.70H, Ar-H), 7.19, 7.26 (2s, 2H, SO_2NH_2), 7.28-7.30 (m, 2.60H, Ar-H), 7.39 (d, 0.70H, *J*= 8.0 Hz, Ar-H), 8.08 (dd, 0.30H, *J*= 8.0, 2.4 Hz, Ar-H), 8.20 (dd, 0.70H, *J*= 8.0, 2.4 Hz, Ar-H), 8.36 (d, 0.30H, *J*= 2.4 Hz, Ar-H), 8.42 (d, 0.70H, *J*= 2.4 Hz, Ar-H), 10.90 (s, 1H, NH of isatin). ^{13}C NMR (100 MHz, DMSO) δ (ppm): 14.98, 15.63, 56.87, 57.02, 112.29,112.37, 113.04, 113.56, 114.59, 114.84, 117.18, 117.26, 120.08, 120.31, 126.21, 126.78, 128.43, 130.05, 131.82, 132.12, 132.36, 133.05, 141.56, 146.69, 156.71, 157.46, 157.66, 158.49, 159.77, 164.73. EI-MS: *m/z*: 391.088 $[M+H]^+$. Anal. Calcd. For $C_{17}H_{15}FN_4O_4S$: C, 52.30; H, 3.87; N, 14.35; S, 8.21. Found: C, 52.11; H, 3.95; N, 14.50; S, 8.18.

5-[(1*E*)-1-(2-[(3*E*)-7-Fluoro-2-oxo-2,3-dihydro-1*H*-indol-3-ylidene]hydrazin-1-ylidene) ethyl]-2-methoxybenzene-1-sulfonamide (**6c**).

Yield (78%) as a red powder, *E*:*Z* ratio= 75:25, with m.p 266-268°C. 1H NMR (500 MHz, DMSO-d_6) δ (ppm): 2.28, 2.32 (2s, 3H, CH_3), 3.95, 3.98 (2s, 3H, OCH_3), 6.95-6.99 (m, 1.50H, Ar-H), 7.16, 7.23 (2s, 2H, SO_2NH_2), 7.25-7.37 (m, 2H, Ar-H), 8.06 (dd, 0.50H, *J*= 8.5, 2.25 Hz, Ar-H), 8.17 (dd, 0.75H, *J*= 8.5, 2.25 Hz, Ar-H), 8.26 (d, 0.25H, *J*= 2.4 Hz, Ar-H), 8.33 (d, 0.25H, *J*= 2.4 Hz, Ar-H), 8.39 (d, 0.75H, *J*= 2.4 Hz, Ar-H), 11.39 (s, 1H, NH of isatin). ^{13}C NMR (125 MHz, DMSO) δ (ppm): 14.97, 15.60, 26.91, 30.05, 32.56, 56.28, 56.28, 57.00, 57.14, 68.96, 113.07,113.49, 119.41, 119.44, 120.55, 120.69,123.50, 123.99, 126.19, 126.77, 128.15, 128.45, 129.19, 130.02, 131.80, 132.85, 132.07, 132.11, 132.17, 133.03, 134.88, 145.89, 145.92, 146.10, 148.04, 157.45, 157.64, 158.60, 160.03, 164.40, 196.20, 208.84. EI-MS: *m/z*: 391.088 $[M+H]^+$. Anal. Calcd. For $C_{17}H_{15}FN_4O_4S$: C, 52.30; H, 3.87; N, 14.35; S, 8.21. Found: C, 52.08; H, 3.85; N, 14.23; S, 8.15.

5-[(1*E*)-1-(2-[(3*E*)-5-Chloro-2-oxo-2,3-dihydro-1*H*-indol-3-ylidene]hydrazin-1-ylidene)ethyl]-2-methoxybenzene-1-sulfonamide (**6d**).

Yield (79%) as yellow powder, *E*:*Z* ratio= 75:25, m.p 268-270°C. ^{1}H NMR (400 MHz, DMSO-d_6) δ (ppm): 2.31, 2.37 (2s, 3H, CH_3), 3.98, 4.02 (2s, 3H, OCH_3), 6.94 (d, 1H, *J*= 8.0 Hz, Ar-H), 7.19, 7.25 (2s, 2H, SO_2NH_2), 7.26-7.31 (m, 0.75H, Ar-H), 7.40 (d, 0.75H, *J*= 8.0 Hz, Ar-H), 7.45 (dd, 1H, *J*= 8.0, 2.4 Hz, Ar-H), 7.51 (d, 0.75H, *J*= 2.4 Hz, Ar-H), 8.09 (dd, 0.25H, *J*= 8.0, 2.4 Hz, Ar-H), 8.20 (dd, 0.75H, *J*= 8.0, 2.4 Hz, Ar-H), 8.36 (d, 0.25H, *J*= 2.4 Hz, Ar-H), 8.40 (d, 0.75H, *J*= 2.4 Hz, Ar-H), 11.01 (s, 1H, NH of isatin). ^{13}C NMR (100 MHz, DMSO) δ (ppm): 14.99, 15.72, 56.87, 57.03, 57.14, 112.84, 113.04,113.56, 118.01, 126.21, 126.29, 127.44, 128.42, 130.05, 131.83, 132.14, 132.35, 132.98, 133.22, 143.95, 146.18, 157.66, 158.50, 159.78, 164.43. EI-MS: *m/z*: 407.0586 $[M+H]^+$. Anal. Calcd. For $C_{17}H_{15}ClN_4O_4S$: C, 50.19; H, 3.72; N, 13.77; S, 7.88. Found: C, 49.95; H, 3.75; N, 13.90; S, 8.00.

5-[(1*E*)-1-(2-[(3*E*)-7-Chloro-2-oxo-2,3-dihydro-1*H*-indol-3-ylidene]hydrazin-1-ylidene)ethyl]-2-methoxybenzene-1-sulfonamide (**6e**).

Yield (68%) as a yellow powder, *E*:*Z* ratio= 70:30, m.p 278-280°C. ^{1}H NMR (500 MHz, DMSO -d_6) δ (ppm): 2.31, 2.35 (2s, 3H, CH_3), 3.98, 4.01 (2s, 3H, CH_3), 7.00 (t, 0.7H, *J*= 8.0 Hz, Ar-H),

7.19, 7.28 (2s, 2H, SO_2NH_2), 7.29-7.31 (m, 0.9H, Ar-H), 7.38 (d, 0.7H, *J*= 8.0 Hz, Ar-H), 7.47-7.51 (m, 1.7H, Ar-H), 8.08 (dd, 0.3H, *J*= 8.0, 4.0 Hz, Ar-H), 8.20 (dd, 0.7H, *J*= 8.0, 4.0 Hz, Ar-H), 8.36 (d, 0.3H, *J*= 4.0 Hz, Ar-H), 8.41 (d, 0.7H, *J*= 2.4 Hz, Ar-H), 11.33 (s, 1H, NH of isatin). ^{13}C NMR (125 MHz, DMSO) δ (ppm): 14.99, 15.64, 56.87, 57.02, 113.04,113.51, 123.92, 126.21, 126.43, 126.82, 132.36, 133.09, 133.34. EI-MS: *m/z*: 407.0585 $[M+H]^+$. Anal. Calcd. For $C_{17}H_{15}ClN_4O_4S$: C, 50.19; H, 3.72; N, 13.77; S, 7.88. Found: C, 50.40; H, 3.69; N, 13.65; S, 7.80.

5-[(1*E*)-1-(2-[(3*E*)-5-Bromo-2-oxo-2,3-dihydro-1*H*-indol-3-ylidene]hydrazin-1-ylidene)ethyl]-2-methoxybenzene-1-sulfonamide (**6f**).

Yield (80%) as an orange powder, *E*:*Z* ratio= 65:35, with m.p 274-276°C. ^{1}H NMR (400 MHz, DMSO-*d*$_6$) δ (ppm): 2.30, 2.36 (2s, 3H, CH_3), 3.97, 4.02 (2s, 3H, OCH_3), 6.89 (d, 0.70H, *J*= 8.0 Hz, Ar-H), 7.18, 7.24 (2s, 2H, SO_2NH_2), 7.29 (d, 1.35H, *J*= 8.0 Hz, Ar-H), 7.40 (d, 0.65H, *J*= 8.0 Hz, Ar-H), 7.57 (dd, 0.65H, *J*= 8.0, 2.4 Hz, Ar-H), 7.64 (d, 0.65H, *J*= 2.4 Hz, Ar-H), 8.09 (dd, 0.35H, *J*= 8.0 Hz, Ar-H), 8.20 (dd, 0.65H, *J*= 8.0, 2.4 Hz, Ar-H), 8.36 (d, 0.35H, *J*= 2.4 Hz, Ar-H), 8.39 (d, 0.65H, *J*= 2.4 Hz, Ar-H), 11.01 (s, 1H, NH of isatin). ^{13}C NMR (100 MHz, DMSO) δ (ppm): 14.99, 15.74, 56.87, 57.03, 113.04,113.55, 113.91, 118.50, 126.21, 126.85, 128.41, 130.05, 130.23, 131.82, 132.14, 132.35, 132.92, 136.01, 144.30, 146.01, 157.47, 157.66, 159.62, 164.28. EI-MS: *m/z*: 451.008 $[M+H]^+$. Anal. Calcd. For $C_{17}H_{15}BrN_4O_4S$: C, 45.24; H, 3.35; N, 12.41; S, 7.10. Found: C, 45.02; H, 3.30; N, 12.25; S, 7.08.

5-[(1*E*)-1-(2-[(3*E*)-6-Bromo-2-oxo-2,3-dihydro-1*H*-indol-3-ylidene]hydrazin-1-ylidene)ethyl]-2-methoxybenzene-1-sulfonamide (**6g**).

Yield (73%) as an orange powder, *E*:*Z* ratio= 65:35, with m.p 276-278°C. ^{1}H NMR (500 MHz, DMSO-*d*$_6$) δ (ppm): 2.26, 2.29 (2s, 3H, CH_3), 3.92, 3.95 (2s, 3H, OCH_3), 7.02 (d, 0.65H, *J*= 2.0 Hz, Ar-H), 7.13-7.14 (m, 2H, Ar-H), 7.15, 7.24 (2s, 2H, SO_2NH_2), 7.32 (d, 0.70H, *J*= 9.0 Hz, Ar-H), 7.38 (d, 0.65H, *J*= 8.0 Hz, Ar-H), 8.03 (dd, 0.35H, *J*= 9.0, 2.0 Hz, Ar-H), 8.14 (dd, 0.65H, *J*= 8.0, 2.0 Hz, Ar-H), 8.31 (d, 0.35H, *J*= 2.5 Hz, Ar-H), 8.35 (d, 0.65H, *J*= 2.5 Hz, Ar-H), 11.00 (s, 1H, NH of isatin). ^{13}C NMR (125 MHz, DMSO) δ (ppm): 14.52, 15.14, 56.39, 56.54, 112.56, 113.02,113.67, 115.51, 125,01, 126.34, 126.42, 132.58, 145.57, 146.01, 157.01,

157.18, 157.96, 158.51, 164.09. EI-MS: *m/z*: 451.008 $[M+H]^+$. Anal. Calcd. For $C_{17}H_{15}BrN_4O_4S$: C, 45.24; H, 3.35; N, 12.41; S, 7.10. Found: C, 45.06; H, 3.29; N, 12.52; S, 7.14.

2-Methoxy-5-[(1*E*)-1-(2-[(3*E*)-5-methoxy-2-oxo-2,3-dihydro-1*H*-indol-3-ylidene]hydrazin-1-ylidene)ethyl]benzene-1-sulfonamide (**6h**).

Yield (70%) as a red powder, *E*:*Z* ratio= 75:25, with m.p 264-266°C. ^1H NMR (400 MHz, DMSO-d_6) δ (ppm): 2.31, 2.35 (2s, 3H, CH_3), 3.62 (s, 3H, OCH_3), 3.97, 4.01 (2s, 3H, OCH_3), 6.84 (d, 0.75H, *J*= 8.0 Hz, Ar-H), 6.99 (dd, 0.75H, *J*= 8.5, 2.6 Hz, Ar-H), 7.17 (d, 1.50H, *J*= 2.4 Hz, Ar-H), 7.19, 7.23 (2s, 2H, SO_2NH_2), 7.30 (d, 0.25H, *J*= 8.8 Hz, Ar-H), 7.38 (d, 0.75H, *J*= 8.8 Hz, Ar-H), 8.09 (dd, 0.25H, *J*= 8.8, 2.4 Hz, Ar-H), 8.20 (dd, 0.75H, *J*= 8.8, 2.4 Hz, Ar-H), 8.36 (d, 0.25H, *J*= 2.4 Hz, Ar-H), 8.46 (d, 0.75H, *J*= 2.4 Hz, Ar-H), 10.67 (s, 1H, NH of isatin). ^{13}C NMR (100 MHz, DMSO) δ (ppm): 14.99, 15.43, 49.06, 55.87, 56.87, 57.00, 111.93, 113.05,113.45, 113.49, 117.25, 119.41, 126.21, 126.50, 128.56, 130.05, 131.83, 132.15, 132.35, 132.96, 138.82, 147.10, 155.09, 157.47, 157.66, 158.32, 158.64, 164.77. EI-MS: *m/z*: 403.108 $[M+H]^+$. Anal. Calcd. For $C_{18}H_{18}N_4O_5S$: C, 53.72; H, 4.51; N, 13.92; S, 7.97. Found: C, 53.99; H, 4.45; N, 14.02; S, 7.93.

2-Methoxy-5-[(1*E*)-1-(2-[(3*E*)-2-oxo-5-(trifluoromethoxy)-2,3-dihydro-1*H*-indol-3-ylidene]-hydrazin-1-ylidene)ethyl]benzene-1-sulfonamide (**6i**).

Yield (76%) as an orange powder, *E*:*Z* ratio= 60:40, with m.p 270-272°C. ^1H NMR (500 MHz, DMSO-d_6) δ (ppm): 2.26, 2.34 (2s, 3H, CH_3), 3.92, 3.97 (2s, 3H, OCH_3), 6.96 (d, 0.60H, *J*= 9.0 Hz, Ar-H), 7.15 (s, 1.20H, Ar-H), 7.18 (s, 2H, SO_2NH_2), 7.25 (d, 0.40H, *J*= 8.5 Hz, Ar-H), 7.34 (d, 0.60H, *J*= 9.0 Hz, Ar-H), 7.38 (d, 0.60H, *J*= 8.0 Hz, Ar-H), 7.46 (s, 0.60H, Ar-H), 8.03 (dd, 0.40H, *J*= 8.0, 2.5 Hz , Ar-H), 8.15 (dd, 0.60H, dd, *J*= 8.0, 2.5 Hz , Ar-H), 8.31 (d, 0.40H, *J*= 2.5 Hz , Ar-H), 8.35 (d, 0.60H, *J*= 2.5 Hz , Ar-H), 11.02 (s, 1H, NH of isatin). ^{13}C NMR (125 MHz, DMSO) δ (ppm): 14.51, 15.19, 56.39, 56.57, 111.92, 112.99, 116.89, 120.32, 125.72, 126.28, 126.38, 127.85, 131.67, 131.88, 132.43, 142.85, 143.70, 146.10, 157.01, 157.18, 158.08, 160.05, 164.24. EI-MS: *m/z*: 457.082 $[M+H]^+$. Anal. Calcd. For $C_{18}H_{15}F_3N_4O_5S$: C, 47.37; H, 3.31; N, 12.28; S, 7.02. Found: C, 47.60; H, 3.33; N, 12.17; S, 7.07.

2-Methoxy-5-[(1*E*)-1-(2-[(3*E*)-1-methyl-2-oxo-2,3-dihydro-1*H*-indol-3-ylidene]hydrazin-1-ylidene)ethyl]benzene-1-sulfonamide (**11a**).

Yield (83%) as a yellow powder, *E*:*Z* ratio= 70:30, with m.p 236-238°C. ^{1}H NMR (500 MHz, DMSO-d_6) δ (ppm): 2.26, 2.28 (2s, 3H, CH_3), 3.30 (s, 3H, CH_3), 3.92, 3.96 (2s, 3H, OCH_3), 6.97 (t, 0.70H, *J*= 8.0 Hz, Ar-H), 7.05 (d, 0.60H, *J*= 8.0 Hz, Ar-H), 7.15, 7.23 (2s, 2H, SO_2NH_2), 7.24-7.25 (m, 1.60H, Ar-H), 7.33 (d, 0.70H, *J*= 9.0 Hz, Ar-H), 7.43 (t, 0.70H, *J*= 9.0 Hz, Ar-H), 7.49 (d, 0.70H, *J*= 9.0 Hz, Ar-H), 8.03 (dd, 0.30H, *J*= 8.0, 2.0 Hz, Ar-H), 8.15 (dd, 0.70H, *J*= 8.0, 2.0 Hz, Ar-H), 8.31 (d, 0.30H, *J*= 2.5 Hz, Ar-H), 8.37 (d, 0.70H, *J*= 2.5 Hz, Ar-H). ^{13}C NMR (125 MHz, DMSO) δ (ppm): 14.51, 15.06, 26.02, 56.39, 56.52, 109.54, 112.56, 113.01, 115.70, 122.73, 125.73, 126.26, 127.11, 128.08, 131.63, 132.50, 133.30, 145.45, 145.80, 157.01, 157.18, 157.71, 157.88, 162.82. EI-MS: *m/z*: 387.112 $[M+H]^+$. Anal. Calcd. For $C_{18}H_{18}N_4O_4S$: C, 55.95; H, 4.70; N, 14.50; S, 8.30. Found: C, 56.20; H, 4.75; N, 14.39; S, 8.21.

5-[(1*E*)-1-(2-[(3*E*)-5-Chloro-1-methyl-2-oxo-2,3-dihydro-1*H*-indol-3-ylidene]hydrazin-1-ylidene)ethyl]-2-methoxybenzene-1-sulfonamide (**11b**).

Yield (80%) as a yellow powder, *E*:*Z* ratio= 70:30, with m.p 248-250°C. ^{1}H NMR (400 MHz, DMSO-d_6) δ (ppm): 2.31, 2.37 (2s, 3H, CH_3), 3.21 (s, 3H, CH_3) 3.98, 4.02 (2s, 3H, OCH_3), 7.14 (d, 1H, *J*= 8.0 Hz, Ar-H), 7.19, 7.25 (2s, 2H, SO_2NH_2), 7.30 (d, 0.70H, *J*= 8.5 Hz, Ar-H), 7.40 (d, 0.70H, *J*= 8.5 Hz, Ar-H), 7.53-7.57 (m, 1.60H, Ar-H), 8.09 (dd, 0.30H, *J*= 8.5, 2.15 Hz, Ar-H), 8.20 (dd, 0.70H, *J*= 8.0, 2.15 Hz, Ar-H), 8.35 (d, 0.30H, *J*= 2.15 Hz, Ar-H), 8.41 (d, 0.70H, *J*= 2.15 Hz, Ar-H). ^{13}C NMR (100 MHz, DMSO) δ (ppm): 14.99, 15.77, 26.67, 49.07, 56.87, 57.04, 111.61, 113.05, 113.56, 117.33, 126.21, 126.86, 127.16, 128.36, 130.05, 131.83, 132.15, 132.35, 133.03, 145.06, 145.52, 147.27, 157.47, 157.66, 158.54, 159.95, 163.07. EI-MS: *m/z*: 421.074 $[M+H]^+$. Anal. Calcd. For $C_{18}H_{17}ClN_4O_4S$: C, 51.37; H, 4.07; N, 13.31; S, 7.62. Found: C, 51.11; H, 4.04; N, 13.20; S, 7.55.

5-[(1*E*)-1-(2-[(3*E*)-5-Bromo-1-methyl-2-oxo-2,3-dihydro-1*H*-indol-3-ylidene]hydrazin-1-ylidene)ethyl]-2-methoxybenzene-1-sulfonamide (**11c**).

Yield (85%) as an orange powder, *E*:*Z* ratio= 65:35, with m.p 263-265°C. ^{1}H NMR (500 MHz, DMSO-d_6) δ (ppm): 2.28, 2.34 (2s, 3H, CH_3), 3.18 (s, 3H, s, CH_3), 3.95, 4.00 (2s, 3H, OCH_3), 7.08 (d, 0.65H, *J*= 8.5 Hz, Ar-H), 7.17, 7.23 (2s, 2H, SO_2NH_2), 7.27 (d, 1.35H, *J*= 9.0 Hz, Ar-H), 7.38 (d, 0.65H, *J*= 9.0 Hz, Ar-H), 7.63-7.65 (m, 1.35H, Ar-H), 8.06 (dd, 0.35H, *J*= 8.5, 2.5 Hz, Ar-H), 8.18 (dd, 0.65H, *J*= 9.0, 2.5 Hz, Ar-H), 8.34 (d, 0.35H, *J*= 2.5 Hz, Ar-H), 8.38 (d,

0.65H, J= 2.5 Hz Ar-H). ^{13}C NMR (125 MHz, DMSO) δ (ppm): 14.95, 15.78, 26.63, 56.84, 57.04, 112.02, 113.01, 113.51, 114.50, 117.73, 126.18, 126.85, 128.33, 129.86, 145.28, 145.39, 157.41, 157.69, 158.48, 159.77, 162.91. EI-MS: *m/z*: 465.023 $[M+H]^+$. Anal. Calcd. For $C_{18}H_{17}BrN_4O_4S$: C, 46.46; H, 3.68; N, 12.04; S, 6.89. Found: C, 46.69; H, 3.72; N, 12.19; S, 6.99.

5-[(1*E*)-1-(2-[(3*E*)-1-Benzyl-2-oxo-2,3-dihydro-1*H*-indol-3-ylidene]hydrazin-1-ylidene)ethyl]-2-methoxybenzene-1-sulfonamide (**12a**).

Yield (86%) as a yellow powder, *E*:*Z* ratio= 65:35, with m.p 235-237°C. ^{1}H NMR (500 MHz, DMSO-d_6) δ (ppm): 2.29, 2.35 (2s, 3H, CH_3), 3.95, 3.99 (2s, 3H, OCH_3), 4.98 (s, 2H, CH_2), 6.98-7.02 (m, 1.65H, Ar-H), 7.17, 7.25 (2s, 2H, SO_2NH_2), 7.28 (d, 2.35H, J= 8.0 Hz, Ar-H), 7.32-7.40 (m, 5.35H, Ar-H), 7.57 (d, 0.65H, J= 8.0 Hz, Ar-H), 8.06 (dd, 0.35H, J= 8.0, 2.5 Hz, Ar-H), 8.18 (dd, 0.65H, J = 8.0, 2.5 Hz, Ar-H), 8.34 (d, 0.35H, d, J= 2.5 Hz, Ar-H), 8.41 (d, 0.65H, J= 2.5 Hz, Ar-H). ^{13}C NMR (125 MHz, DMSO) δ (ppm): 15.08, 15.71, 56.85, 56.98, 110.55, 113.03, 113.50, 116.37, 123.40, 126.18, 126.78, 127.74, 127.88, 127.98, 128.53, 129.18, 130.02, 131.79, 132.08, 132.33, 133.68, 136.54, 145.25, 145.78, 157.44, 157.66, 158.42, 158.58, 163.45. EI-MS: *m/z*: 463.144 $[M+H]^+$. Anal. Calcd. For $C_{24}H_{22}N_4O_4S$: C, 62.32; H, 4.79; N, 12.11; S, 6.93. Found: C, 62.00; H, 4.78; N, 12.03; S, 7.00.

5-[(1*E*)-1-(2-[(3*E*)-1-Benzyl-5-chloro-2-oxo-2,3-dihydro-1*H*-indol-3-ylidene]hydrazin-1-ylidene)ethyl]-2-methoxybenzene-1-sulfonamide (**12b**).

Yield (82%) as an orange powder, *E*:*Z* ratio= 50:50, with m.p 245-247°C. ^{1}H NMR (500 MHz, DMSO-d_6) δ (ppm): 2.26, 2.36 (2s, 3H, CH_3), 3.92, 3.97 (2s, 3H, OCH_3), 4.95 (s, 2H, CH_2), 7.00 (d, 0.50H, J= 8.0 Hz, Ar-H), 7.15, 7.22 (2s, 2H, SO_2NH_2), 7.24-7.25 (m, 3.0H, Ar-H), 7.29-7.37 (m, 4.50H, Ar-H), 7.43 (d, 0.50H, J= 8.0 Hz, Ar-H),), 7.53 (d, 0.50H, J= 2.0 Hz, Ar-H), 8.07 (dd, 0.50H, J= 9.0, 2.0 Hz, Ar-H), 8.17 (dd, 0.50H, J= 9.0, 2.0 Hz, Ar-H), 8.31 (d, 0.50H, J = 2.0 Hz, Ar-H), 8.37 (d, 0.50H, J = 2.0 Hz, Ar-H). ^{13}C NMR (125 MHz, DMSO) δ (ppm): 14.51, 15.40, 42.86, 56.39, 56.57, 111.64, 112.56, 113.11, 117.14, 127.28, 128.76, 131.88, 132.62, 143.50, 144.93, 157.01, 157.18, 158.11, 159.89, 162.77. EI-MS: *m/z*: 497.105 $[M+H]^+$. Anal. Calcd. For $C_{24}H_{21}ClN_4O_4S$: C, 58.00; H, 4.26; N, 11.27; S, 6.45. Found: C, 57.75; H, 4.28; N, 11.18; S, 6.50.

5-[(1*E*)-1-(2-[(3*E*)-1-Benzyl-5-bromo-2-oxo-2,3-dihydro-1*H*-indol-3-ylidene]hydrazin-1-ylidene)ethyl]-2-methoxybenzene-1-sulfonamide (**12c**).

Yield (88%) as an orange powder, *E*:*Z* ratio= 65:35, with m.p 260-262°C. ^{1}H NMR (500 MHz, DMSO-d_6) δ (ppm): 2.31, 2.41 (2s, 3H, CH_3), 3.98, 4.02 (2s, 3H, OCH_3), 5.00 (s, 2H, CH_2), 7.00 (d, 0.65H, *J*= 9.0 Hz, Ar-H), 7.20, 7.25 (2s, 2H, SO_2NH_2), 7.25-7.28 (m, 2.65H, Ar-H), 7.33-7.42 (m, 4.40H, Ar-H), 7.60 (d, 0.65H, *J*= 9.0 Hz, Ar-H), 7.71 (d, 0.65H, *J*= 8.5 Hz, Ar-H), 8.08 (dd, 0.35H, *J*= 9.0, 2.4 Hz, Ar-H), 8.22 (dd, 0.65H, *J* = 8.5, 2.15 Hz, Ar-H), 8.37 (d, 0.35H, *J*= 2.15 Hz, Ar-H), 8.42 (d, 0.65H, *J*= 2.15 Hz, Ar-H). ^{13}C NMR (125 MHz, DMSO) δ (ppm): 15.00, 15.90, 56.88, 57.06, 112.58, 113.05, 113.59, 126.22, 126.95, 127.74, 128.07, 129.23, 130.23, 132.37, 133.03, 135.82, 136.22, 144.35, 145.24, 157.48, 157.67, 158.59, 160.20, 163.12. EI-MS: *m/z*: 541.054 $[M+H]^+$. Anal. Calcd. For $C_{24}H_{21}BrN_4O_4S$: C, 53.24; H, 3.91; N, 10.35; S, 5.92. Found: C, 53.45; H, 3.90; N, 10.27; S, 5.95.

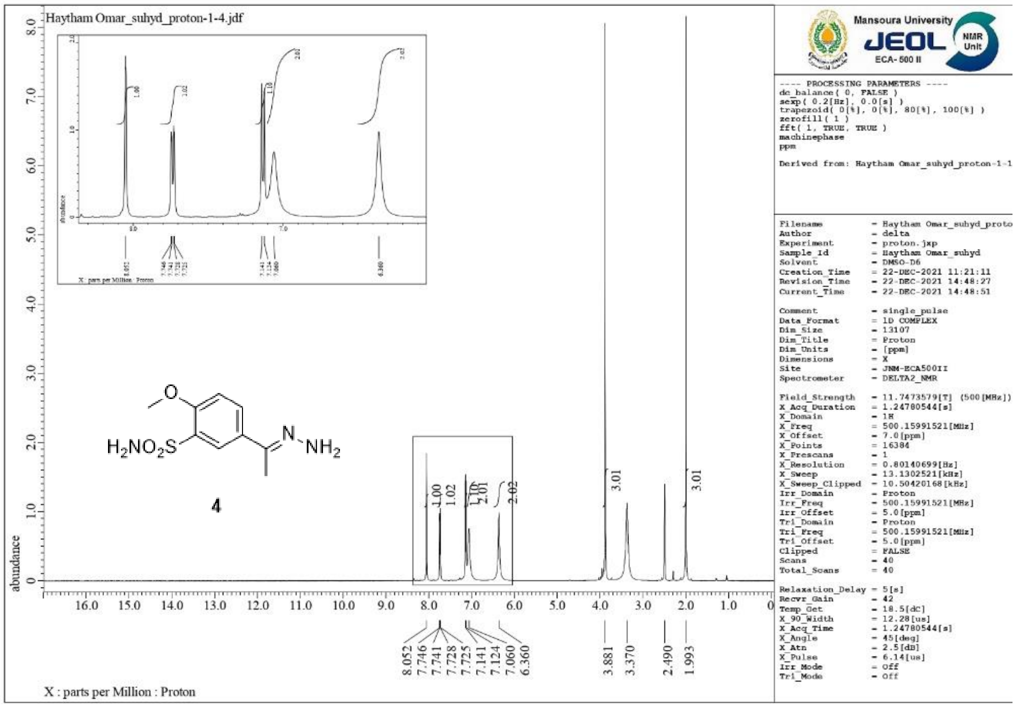

Figure S1. ^{1}H NMR (500 MHz, DMSO-*d6*) spectrum of compound **4**

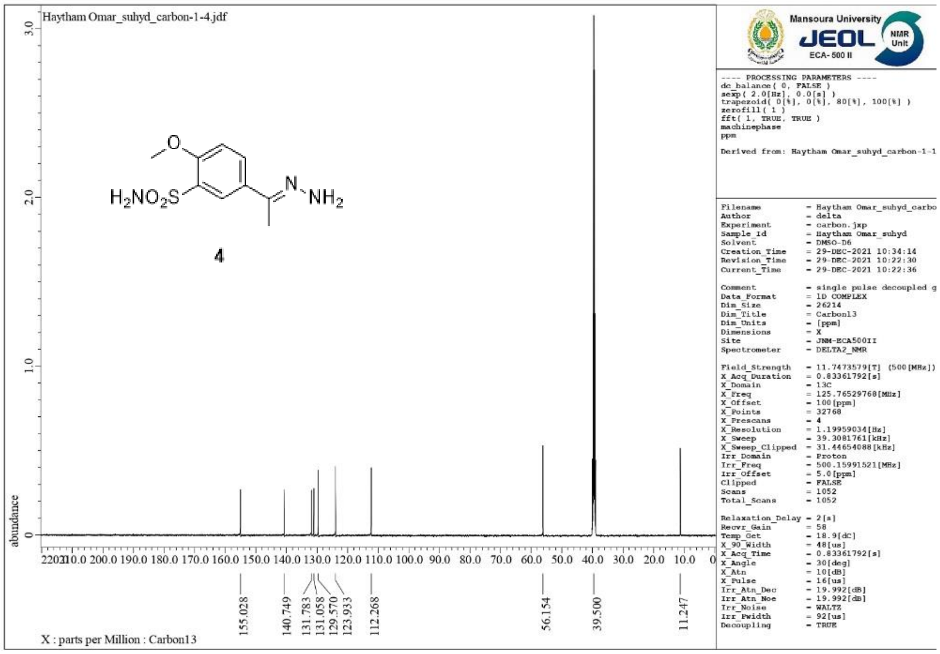

Figure S2. ^{13}C NMR (125 MHz, DMSO-*d6*) spectrum of compound **4**

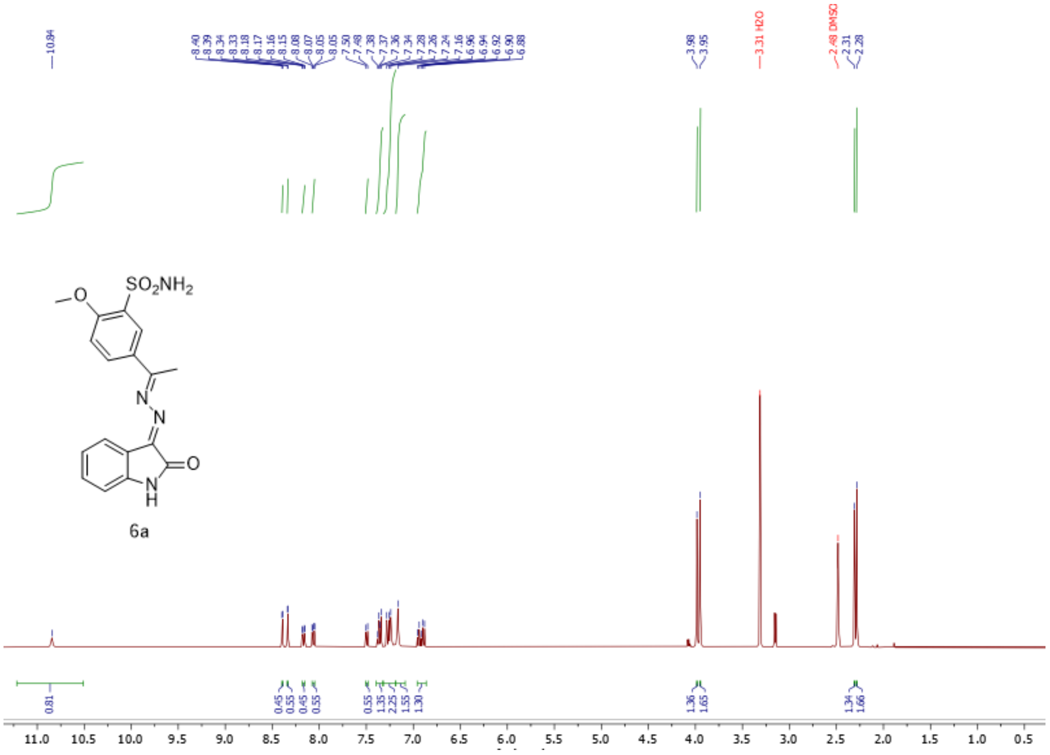

Figure S3. ^{1}H NMR (400 MHz, DMSO-*d6*) spectrum of compound **6a**

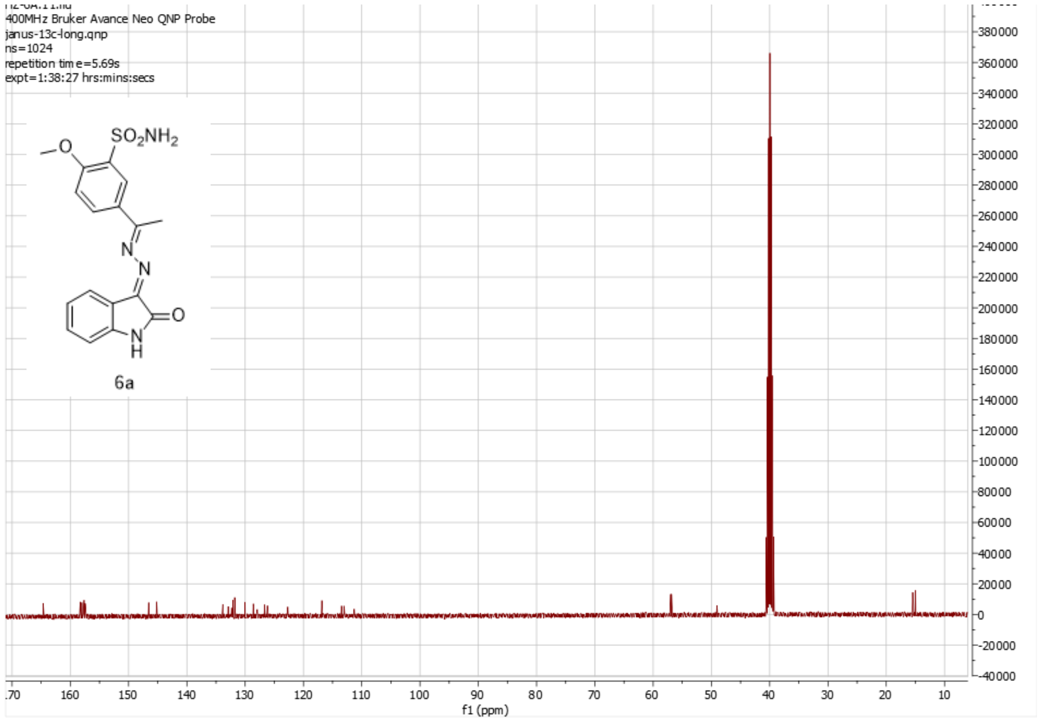

Figure S4. ^{13}C NMR (100 MHz, DMSO-*d6*) spectrum of compound **6a**

WalkUp FbF Workflow Report

UNIVERSITY OF CAMBRIDGE

Spectrum Peaks

m/z	m/z (Calc)	Diff (ppm)	Abund	Height %	Height % (Calc)	Ion Species	Z
373.0975	373.0965	2.78	549410	100.00	100.00	(M+H)+	1
374.1006	374.0993	3.50	116101	21.13	20.99	(M+H)+	1
375.0976	375.0960	4.28	38374	6.98	7.39	(M+H)+	1

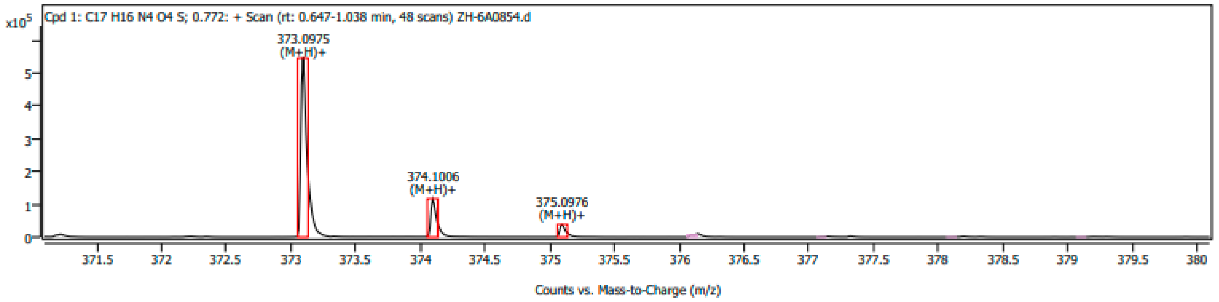

Spectrum Peaks

m/z	m/z (Calc)	Diff (ppm)	Abund	Height %	Height % (Calc)	Ion Species	Z
373.0975	373.0965	2.78	549410	100.00	100.00	(M+H)+	1
374.1006	374.0993	3.50	116101	21.13	20.99	(M+H)+	1
375.0976	375.0960	4.28	38374	6.98	7.39	(M+H)+	1

MassHunter Qual 10.0
(End of Report)

Figure S5. Mass spectrum of compound **6a**

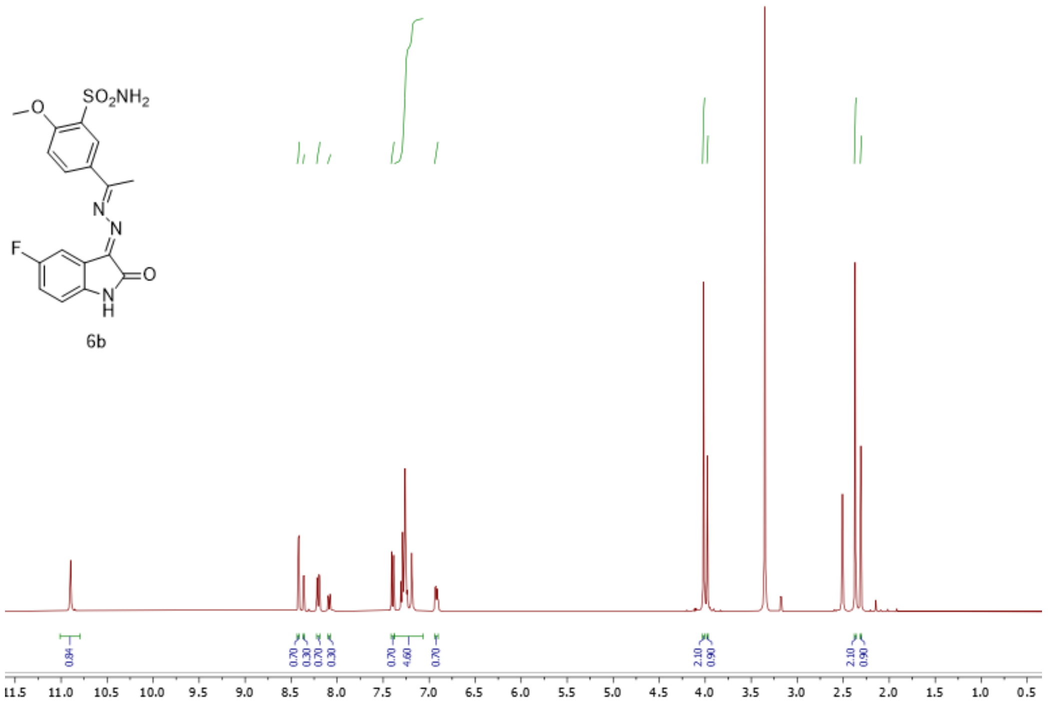

Figure S6. ^{1}H NMR (400 MHz, DMSO-*d6*) spectrum of compound **6b**

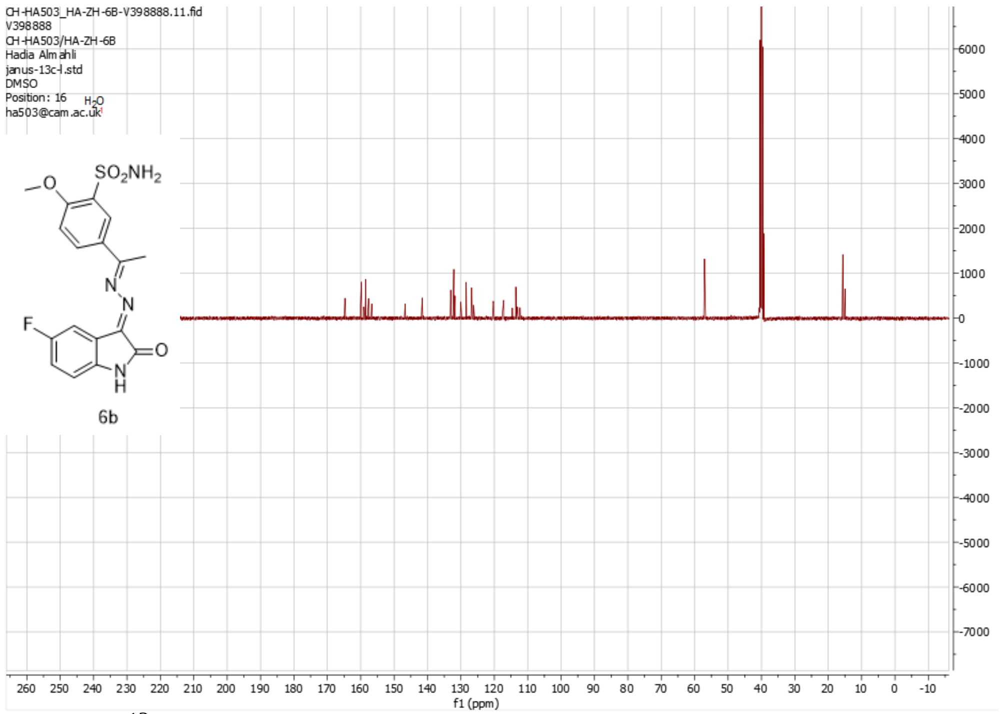

Figure S7. ^{13}C NMR (100 MHz, DMSO-*d6*) spectrum of compound **6b**

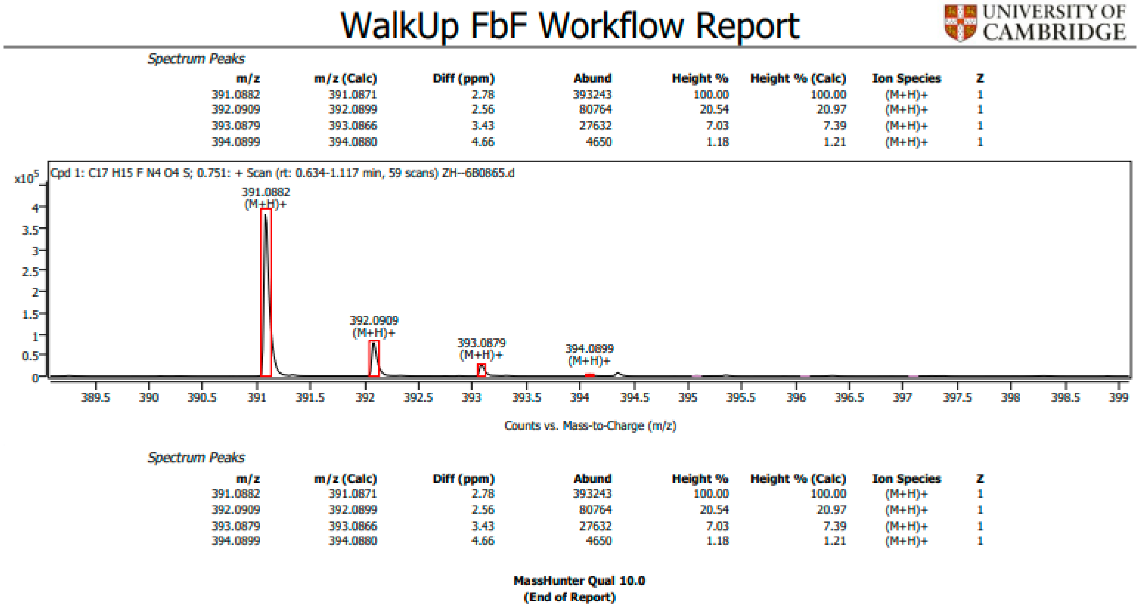

m/z	m/z (Calc)	Diff (ppm)	Abund	Height %	Height % (Calc)	Ion Species	Z
391.0882	391.0871	2.78	393243	100.00	100.00	(M+H)+	1
392.0909	392.0899	2.56	80764	20.54	20.97	(M+H)+	1
393.0879	393.0866	3.43	27632	7.03	7.39	(M+H)+	1
394.0899	394.0880	4.66	4650	1.18	1.21	(M+H)+	1

m/z	m/z (Calc)	Diff (ppm)	Abund	Height %	Height % (Calc)	Ion Species	Z
391.0882	391.0871	2.78	393243	100.00	100.00	(M+H)+	1
392.0909	392.0899	2.56	80764	20.54	20.97	(M+H)+	1
393.0879	393.0866	3.43	27632	7.03	7.39	(M+H)+	1
394.0899	394.0880	4.66	4650	1.18	1.21	(M+H)+	1

Figure S8. Mass spectrum of compound **6b**

WalkUp FbF Workflow Report

UNIVERSITY OF CAMBRIDGE

Spectrum Peaks

m/z	m/z (Calc)	Diff (ppm)	Abund	Height %	Height % (Calc)	Ion Species	z
391.0880	391.0871	2.32	734016	100.00	100.00	(M+H)+	1
392.0911	392.0899	3.14	150282	20.47	20.97	(M+H)+	1
393.0881	393.0866	3.85	50575	6.89	7.39	(M+H)+	1
394.0895	394.0880	3.77	8397	1.14	1.21	(M+H)+	1

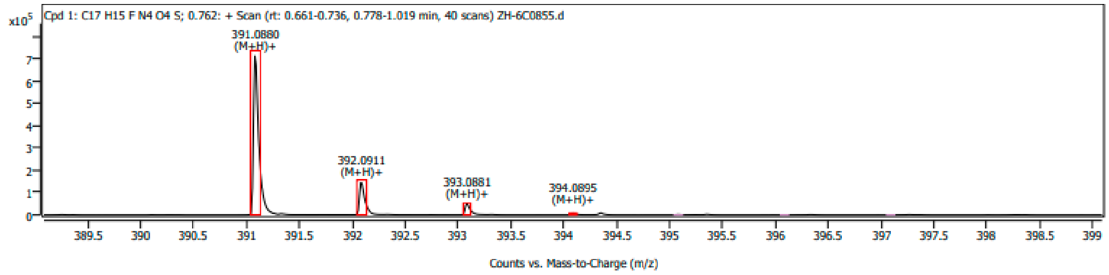

Spectrum Peaks

m/z	m/z (Calc)	Diff (ppm)	Abund	Height %	Height % (Calc)	Ion Species	z
391.0880	391.0871	2.32	734016	100.00	100.00	(M+H)+	1
392.0911	392.0899	3.14	150282	20.47	20.97	(M+H)+	1
393.0881	393.0866	3.85	50575	6.89	7.39	(M+H)+	1
394.0895	394.0880	3.77	8397	1.14	1.21	(M+H)+	1

MassHunter Qual 10.0
(End of Report)

Figure S9. Mass spectrum of compound **6c**

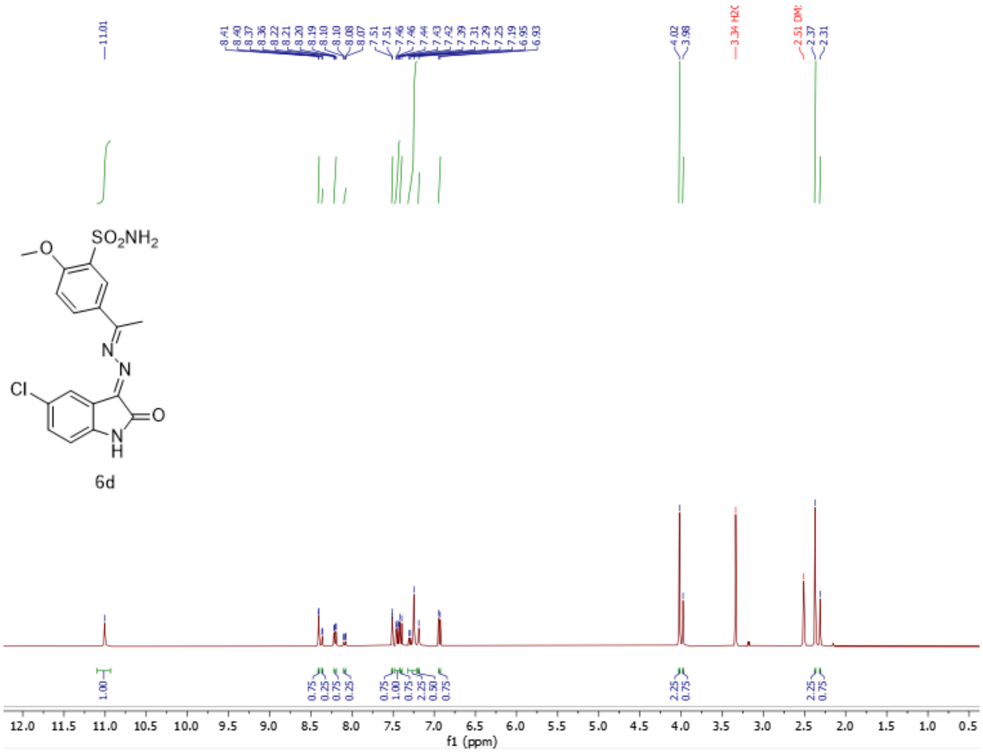

Figure S10. ^{1}H NMR (400 MHz, DMSO-*d6*) spectrum of compound **6d**

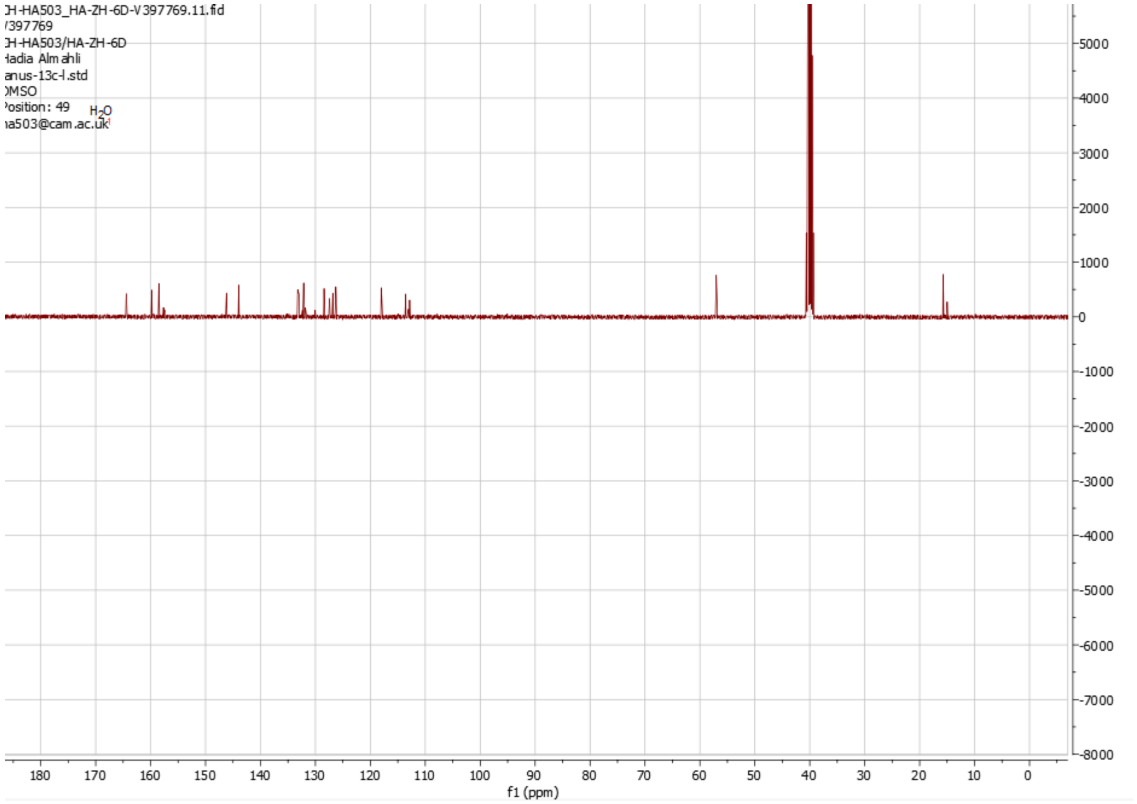

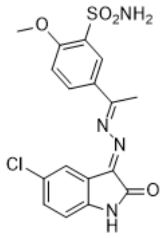

6d

Figure S11. ^{13}C NMR (100 MHz, DMSO-*d6*) spectrum of compound **6d**

WalkUp FbF Workflow Report

UNIVERSITY OF CAMBRIDGE

Spectrum Peaks

m/z	m/z (Calc)	Diff (ppm)	Abund	Height %	Height % (Calc)	Ion Species	Z
407.0586	407.0575	2.65	173417	100.00	100.00	(M+H)+	1
408.0611	408.0603	1.88	37323	21.52	20.97	(M+H)+	1
409.0560	409.0550	2.35	68640	39.58	39.39	(M+H)+	1
410.0580	410.0575	1.22	14131	8.15	7.92	(M+H)+	1
411.0558	411.0544	3.34	4544	2.62	2.52	(M+H)+	1

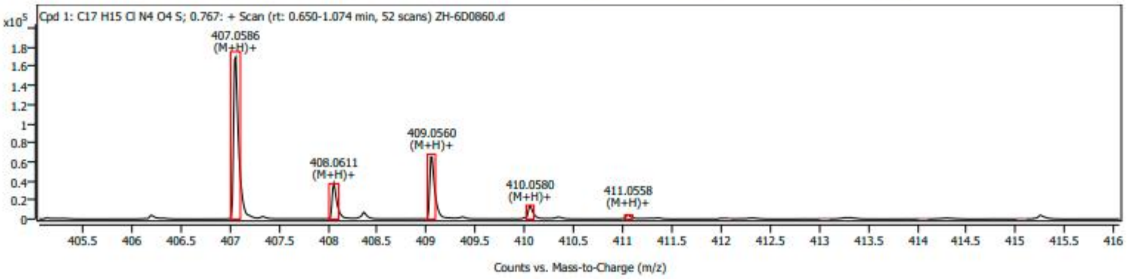

Spectrum Peaks

m/z	m/z (Calc)	Diff (ppm)	Abund	Height %	Height % (Calc)	Ion Species	Z
407.0586	407.0575	2.65	173417	100.00	100.00	(M+H)+	1
408.0611	408.0603	1.88	37323	21.52	20.97	(M+H)+	1
409.0560	409.0550	2.35	68640	39.58	39.39	(M+H)+	1
410.0580	410.0575	1.22	14131	8.15	7.92	(M+H)+	1
411.0558	411.0544	3.34	4544	2.62	2.52	(M+H)+	1

MassHunter Qual 10.0
(End of Report)

Figure S12. Mass spectrum of compound **6d**

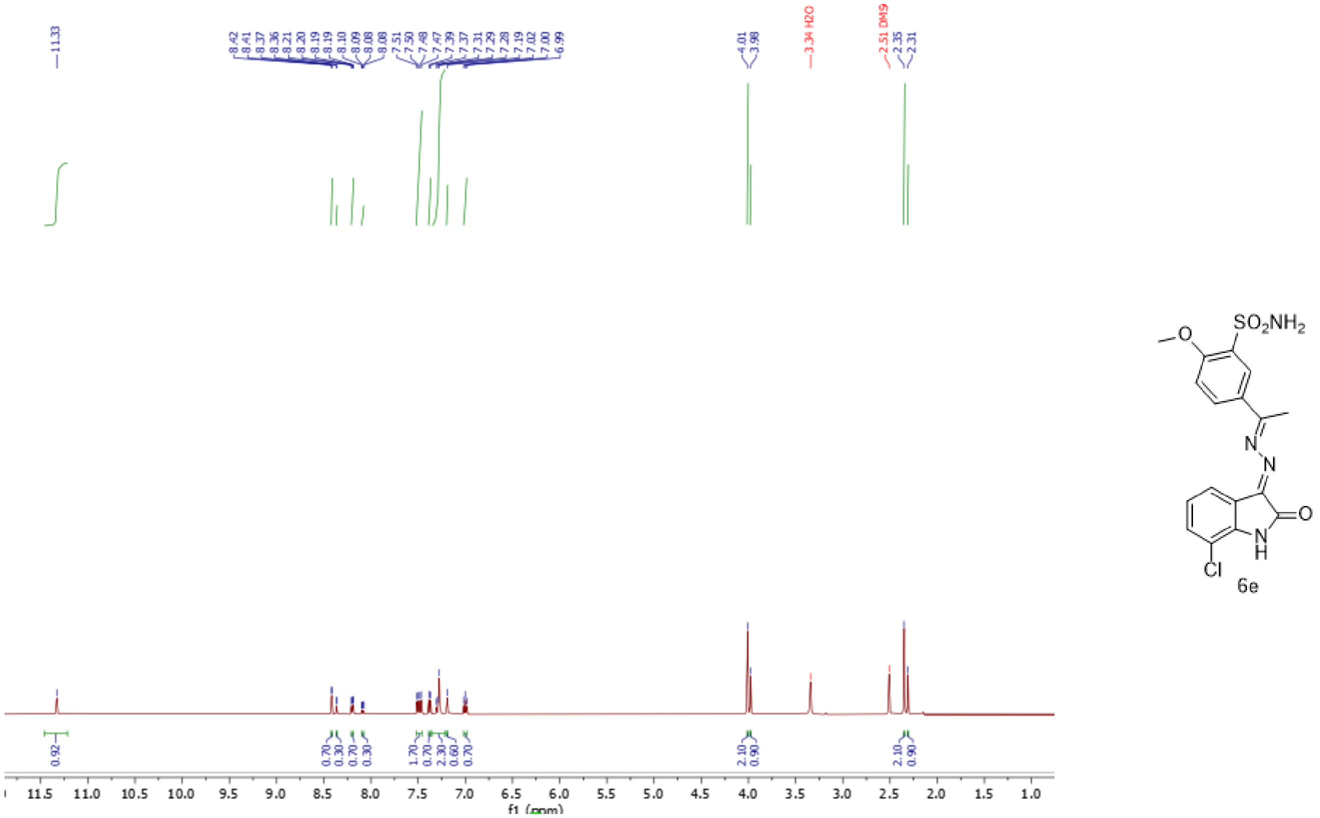

Figure S13. ^{1}H NMR (500 MHz, DMSO-*d6*) spectrum of compound **6e**

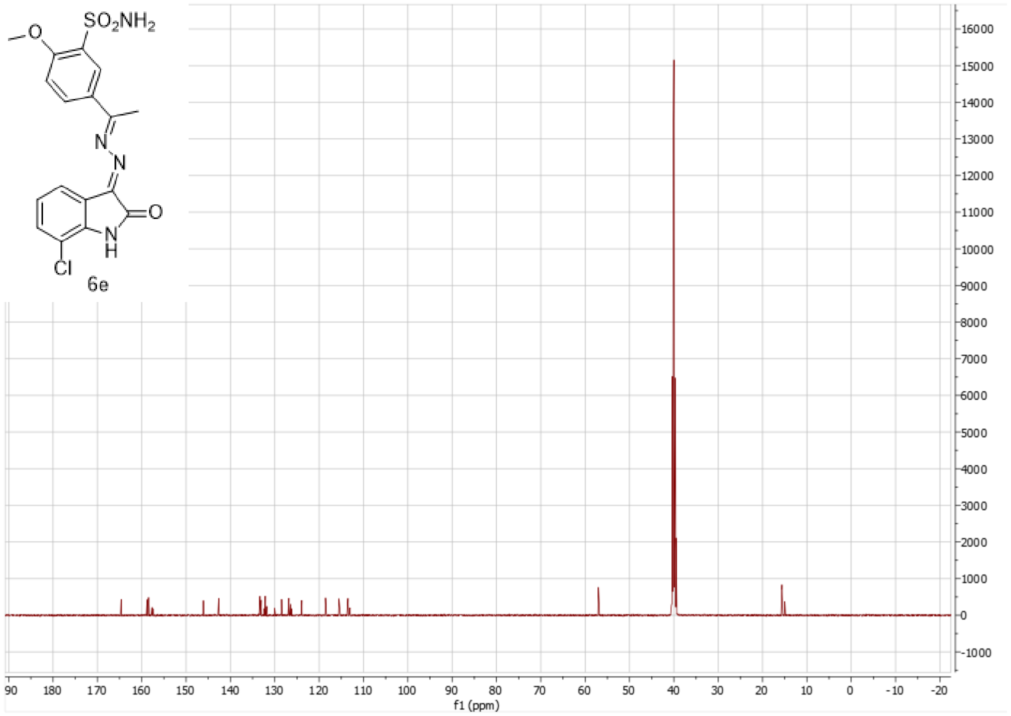

Figure S14. ^{13}C NMR (125 MHz, DMSO-*d6*) spectrum of compound **6e**

WalkUp FbF Workflow Report

Spectrum Peaks

m/z	m/z (Calc)	Diff (ppm)	Abund	Height %	Height % (Calc)	Ion Species	Z
407.0585	407.0575	2.44	471091	100.00	100.00	(M+H)+	1
408.0614	408.0603	2.61	101057	21.45	20.97	(M+H)+	1
409.0563	409.0550	2.97	180931	38.41	39.39	(M+H)+	1
410.0585	410.0575	2.31	37301	7.92	7.92	(M+H)+	1
411.0558	411.0544	3.29	11893	2.52	2.52	(M+H)+	1
412.0563	412.0558	1.38	1897	0.40	0.40	(M+H)+	1

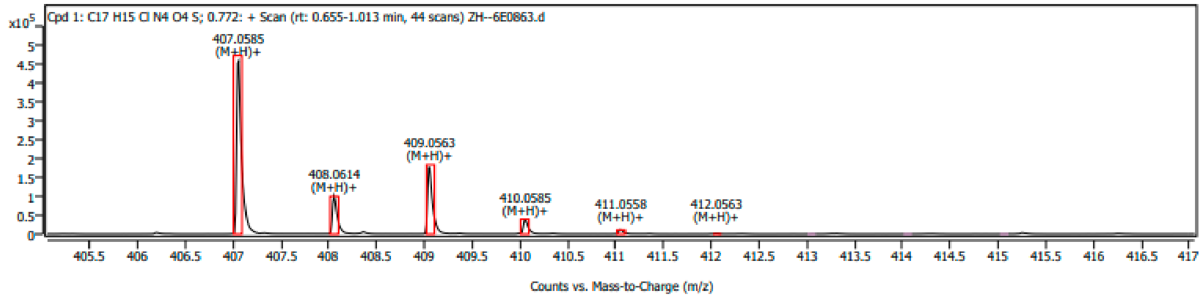

Spectrum Peaks

m/z	m/z (Calc)	Diff (ppm)	Abund	Height %	Height % (Calc)	Ion Species	Z
407.0585	407.0575	2.44	471091	100.00	100.00	(M+H)+	1
408.0614	408.0603	2.61	101057	21.45	20.97	(M+H)+	1
409.0563	409.0550	2.97	180931	38.41	39.39	(M+H)+	1
410.0585	410.0575	2.31	37301	7.92	7.92	(M+H)+	1
411.0558	411.0544	3.29	11893	2.52	2.52	(M+H)+	1
412.0563	412.0558	1.38	1897	0.40	0.40	(M+H)+	1

MassHunter Qual 10.0
(End of Report)

Figure S15. Mass spectrum of compound **6e**

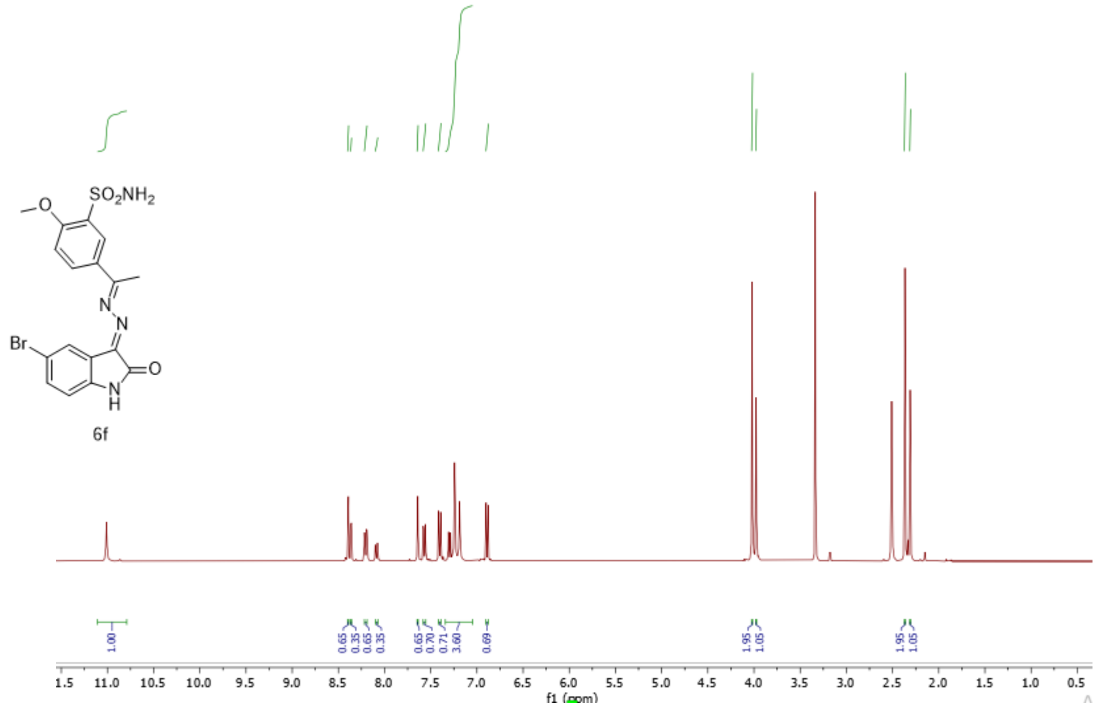

Figure S16. ^{1}H NMR (400 MHz, DMSO-*d6*) spectrum of compound **6f**

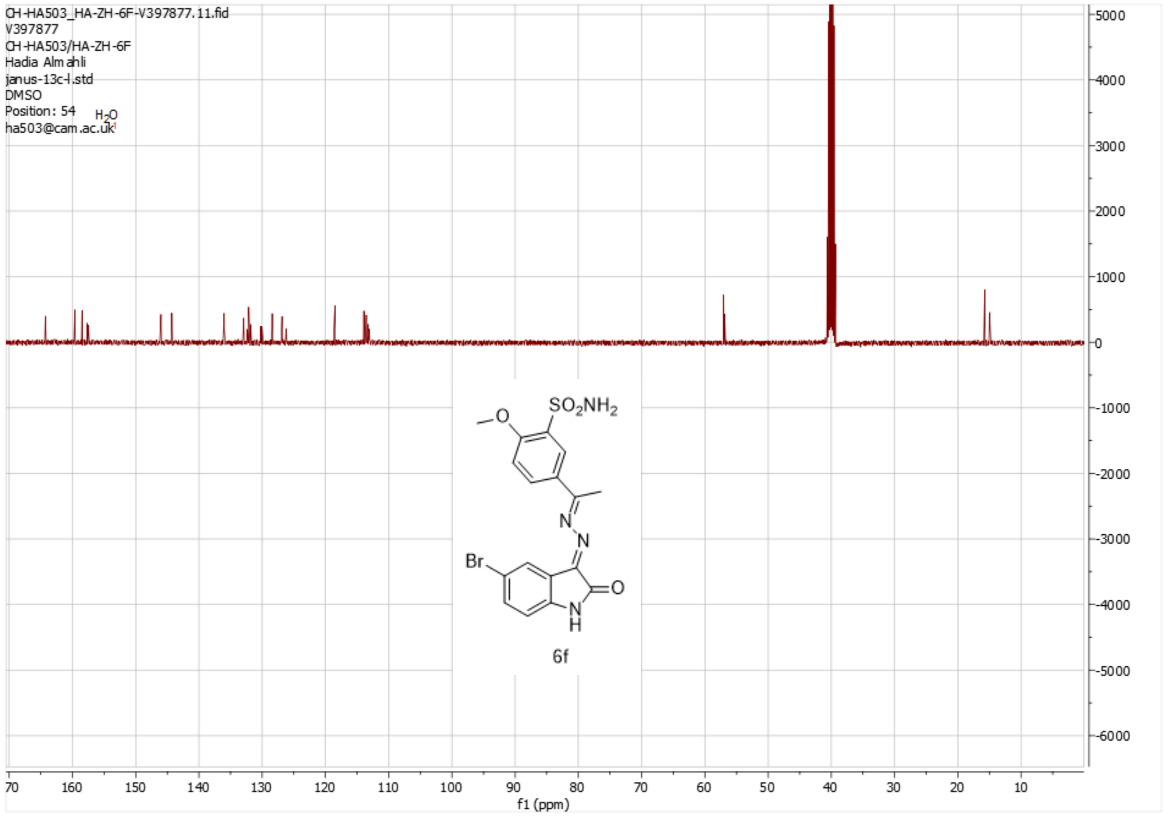

Figure S17. ^{13}C NMR (100 MHz, DMSO-*d6*) spectrum of compound **6f**

WalkUp FbF Workflow Report

UNIVERSITY OF CAMBRIDGE

Spectrum Peaks

m/z	m/z (Calc)	Diff (ppm)	Abund	Height %	Height % (Calc)	Ion Species	Z
451.0080	451.0070	2.18	159100	97.62	95.54	(M+H)+	1
452.0106	452.0098	1.73	33288	20.42	20.04	(M+H)+	1
453.0061	453.0051	2.25	162985	100.00	100.00	(M+H)+	1
454.0085	454.0078	1.68	33992	20.86	20.65	(M+H)+	1
455.0095	455.0046	10.74	11965	7.34	7.02	(M+H)+	1

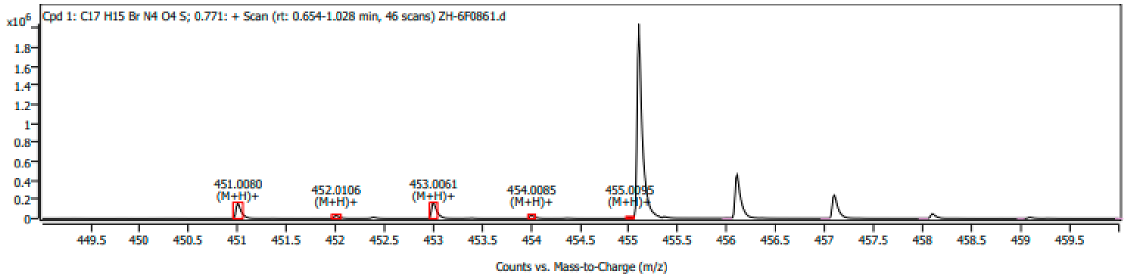

Spectrum Peaks

m/z	m/z (Calc)	Diff (ppm)	Abund	Height %	Height % (Calc)	Ion Species	Z
451.0080	451.0070	2.18	159100	97.62	95.54	(M+H)+	1
452.0106	452.0098	1.73	33288	20.42	20.04	(M+H)+	1
453.0061	453.0051	2.25	162985	100.00	100.00	(M+H)+	1
454.0085	454.0078	1.68	33992	20.86	20.65	(M+H)+	1
455.0095	455.0046	10.74	11965	7.34	7.02	(M+H)+	1

MassHunter Qual 10.0
(End of Report)

Figure S18. Mass spectrum of compound **6f**

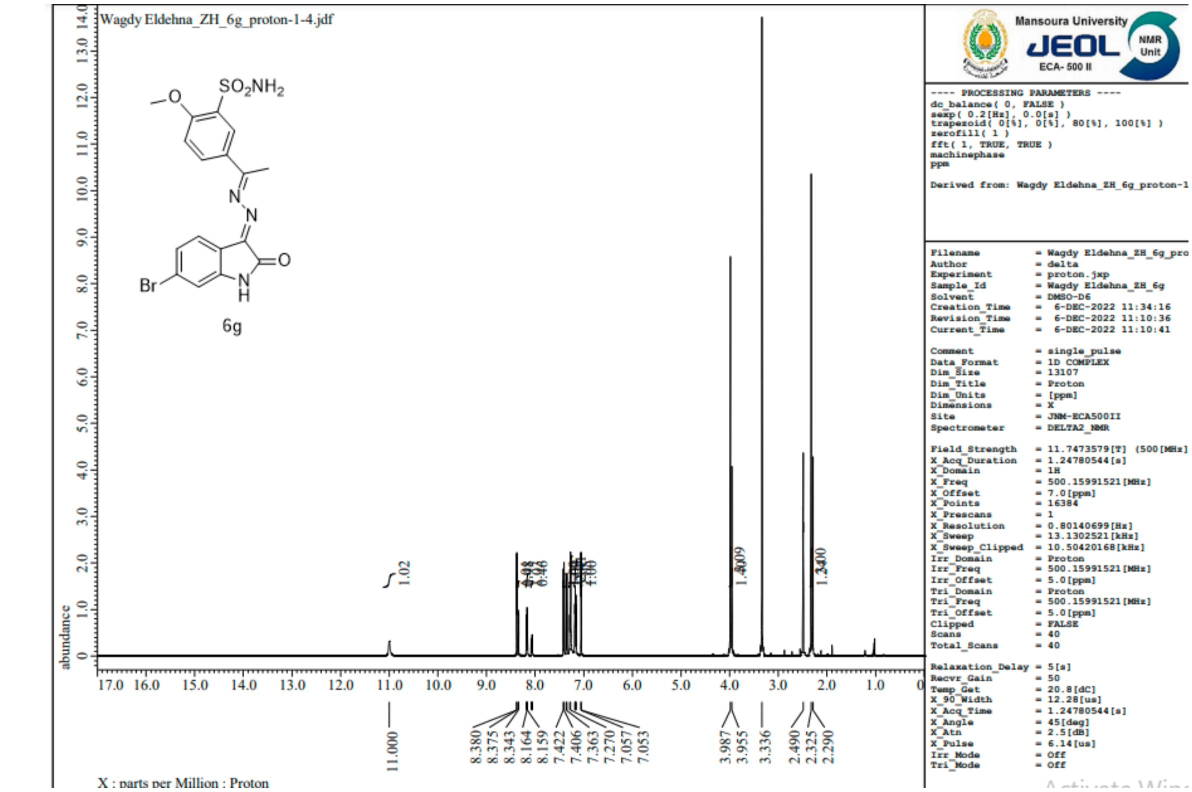

Figure S19. ^{1}H NMR (500 MHz, DMSO-*d6*) spectrum of compound **6g**

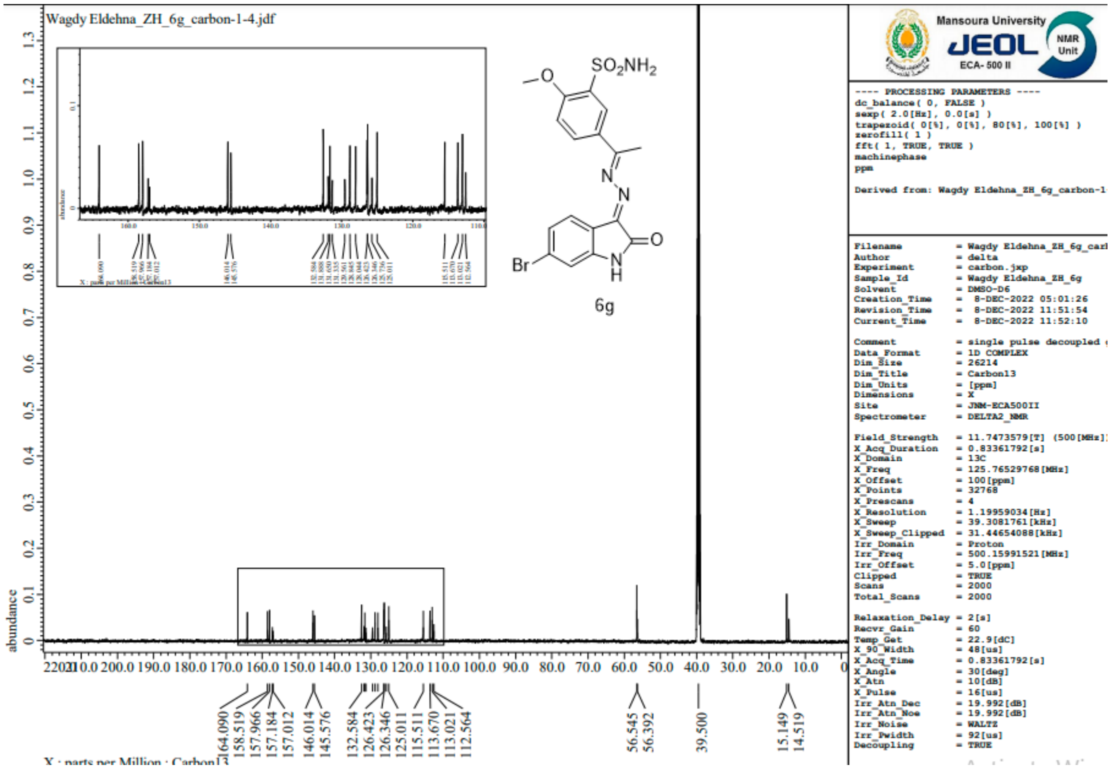

Figure S20. ^{13}C NMR (125 MHz, DMSO-*d6*) spectrum of compound **6g**

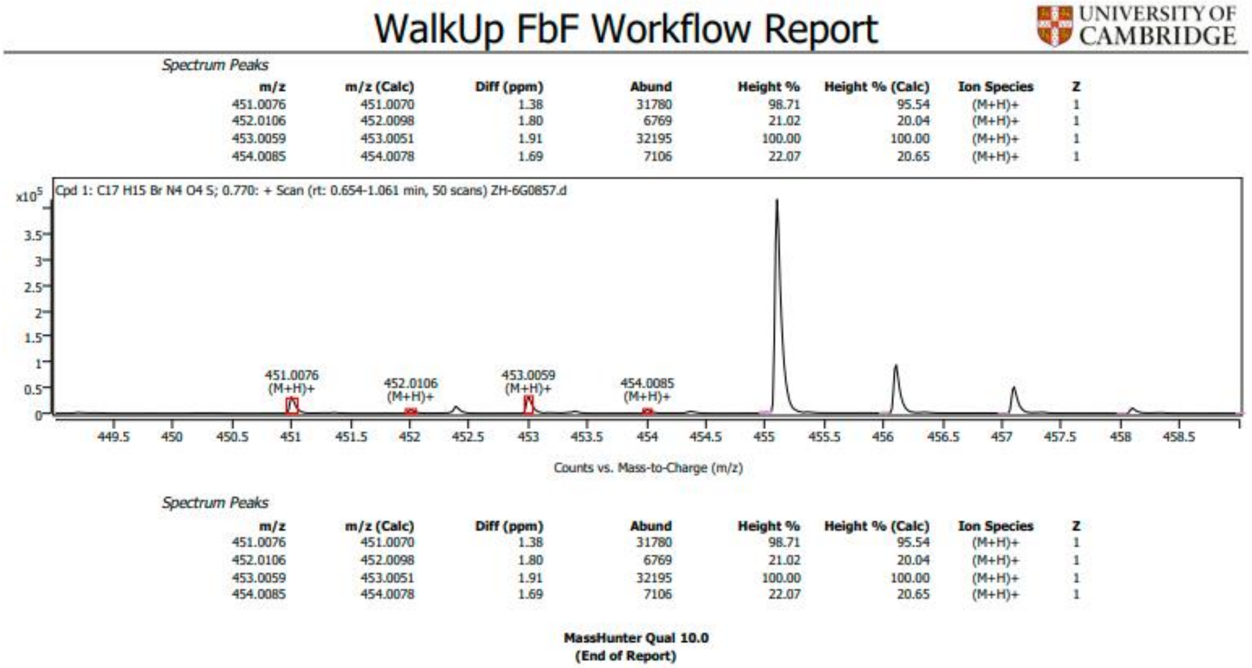

WalkUp FbF Workflow Report

UNIVERSITY OF CAMBRIDGE

Spectrum Peaks

m/z	m/z (Calc)	Diff (ppm)	Abund	Height %	Height % (Calc)	Ion Species	Z
451.0076	451.0070	1.38	31780	98.71	95.54	(M+H)+	1
452.0106	452.0098	1.80	6769	21.02	20.04	(M+H)+	1
453.0059	453.0051	1.91	32195	100.00	100.00	(M+H)+	1
454.0085	454.0078	1.69	7106	22.07	20.65	(M+H)+	1

Spectrum Peaks

m/z	m/z (Calc)	Diff (ppm)	Abund	Height %	Height % (Calc)	Ion Species	Z
451.0076	451.0070	1.38	31780	98.71	95.54	(M+H)+	1
452.0106	452.0098	1.80	6769	21.02	20.04	(M+H)+	1
453.0059	453.0051	1.91	32195	100.00	100.00	(M+H)+	1
454.0085	454.0078	1.69	7106	22.07	20.65	(M+H)+	1

MassHunter Qual 10.0
(End of Report)

Figure S21. Mass spectrum of compound **6g**

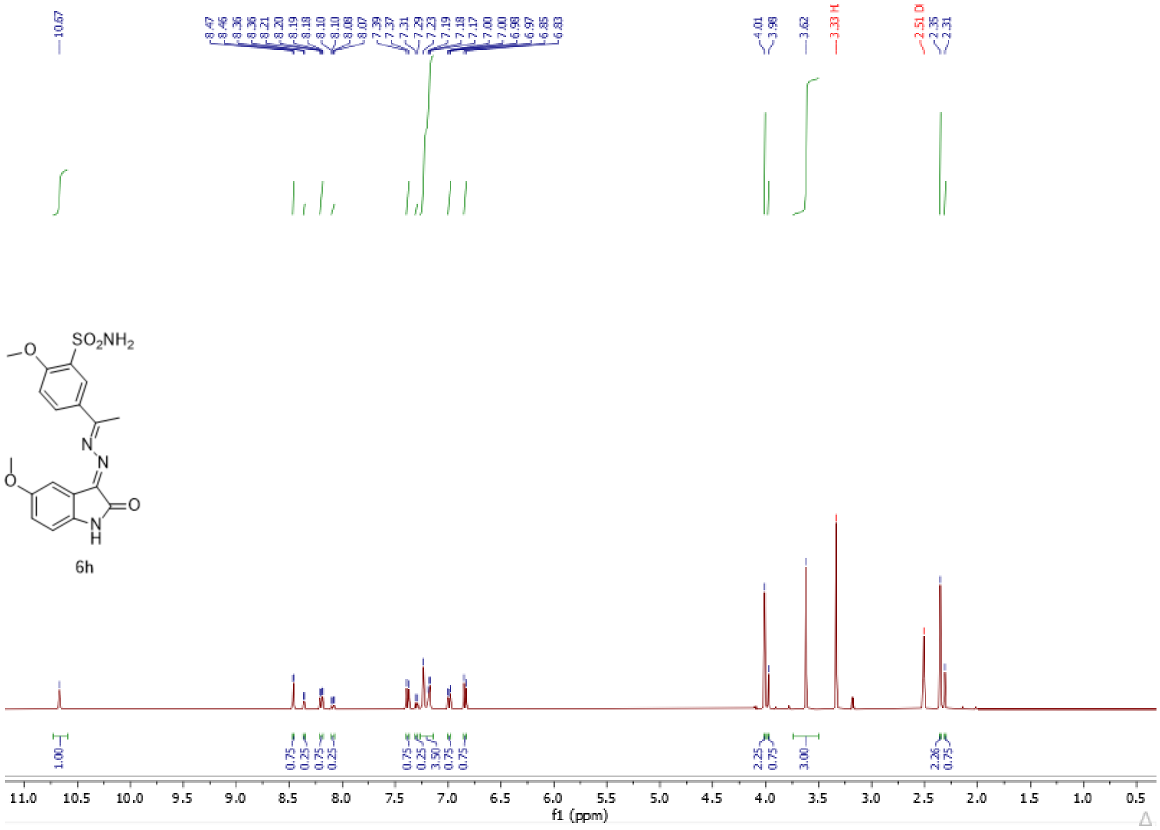

Figure S22. ^{1}H NMR (400 MHz, DMSO-*d6*) spectrum of compound **6h**

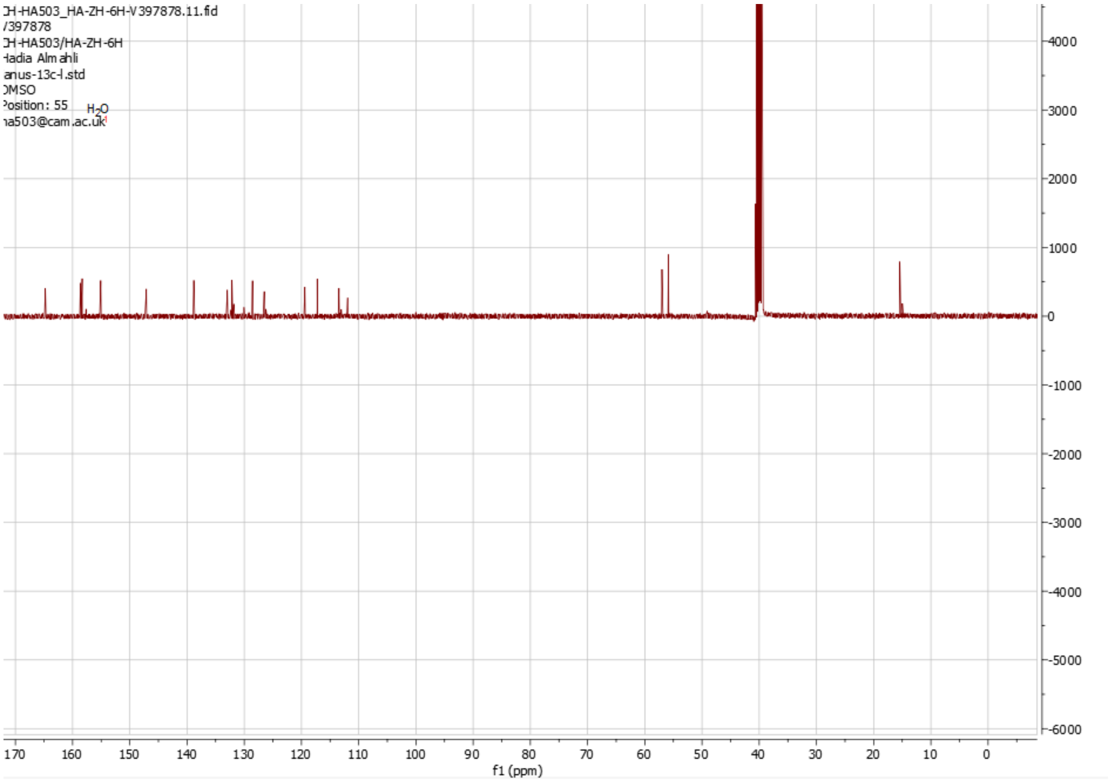

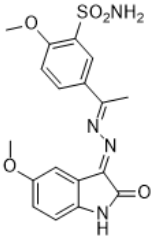

6h

Figure S23. ^{13}C NMR (100 MHz, DMSO-*d6*) spectrum of compound **6h**

Figure S24. Mass spectrum of compound **6h**

WalkUp FbF Workflow Report

UNIVERSITY OF CAMBRIDGE

Spectrum Peaks

m/z	m/z (Calc)	Diff (ppm)	Abund	Height %	Height % (Calc)	Ion Species	Z
403.1082	403.1071	2.70	308633	100.00	100.00	(M+H)+	1
404.1107	404.1099	2.07	68856	22.31	22.13	(M+H)+	1
405.1080	405.1069	2.77	23314	7.55	7.84	(M+H)+	1
406.1097	406.1084	3.17	3954	1.28	1.34	(M+H)+	1

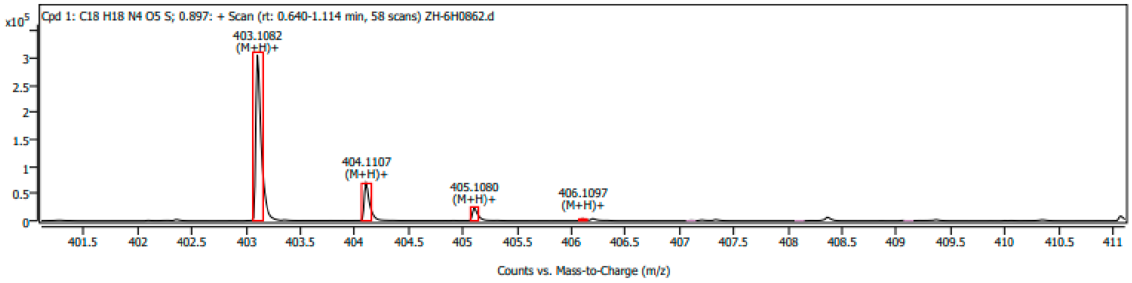

Spectrum Peaks

m/z	m/z (Calc)	Diff (ppm)	Abund	Height %	Height % (Calc)	Ion Species	Z
403.1082	403.1071	2.70	308633	100.00	100.00	(M+H)+	1
404.1107	404.1099	2.07	68856	22.31	22.13	(M+H)+	1
405.1080	405.1069	2.77	23314	7.55	7.84	(M+H)+	1
406.1097	406.1084	3.17	3954	1.28	1.34	(M+H)+	1

MassHunter Qual 10.0
(End of Report)

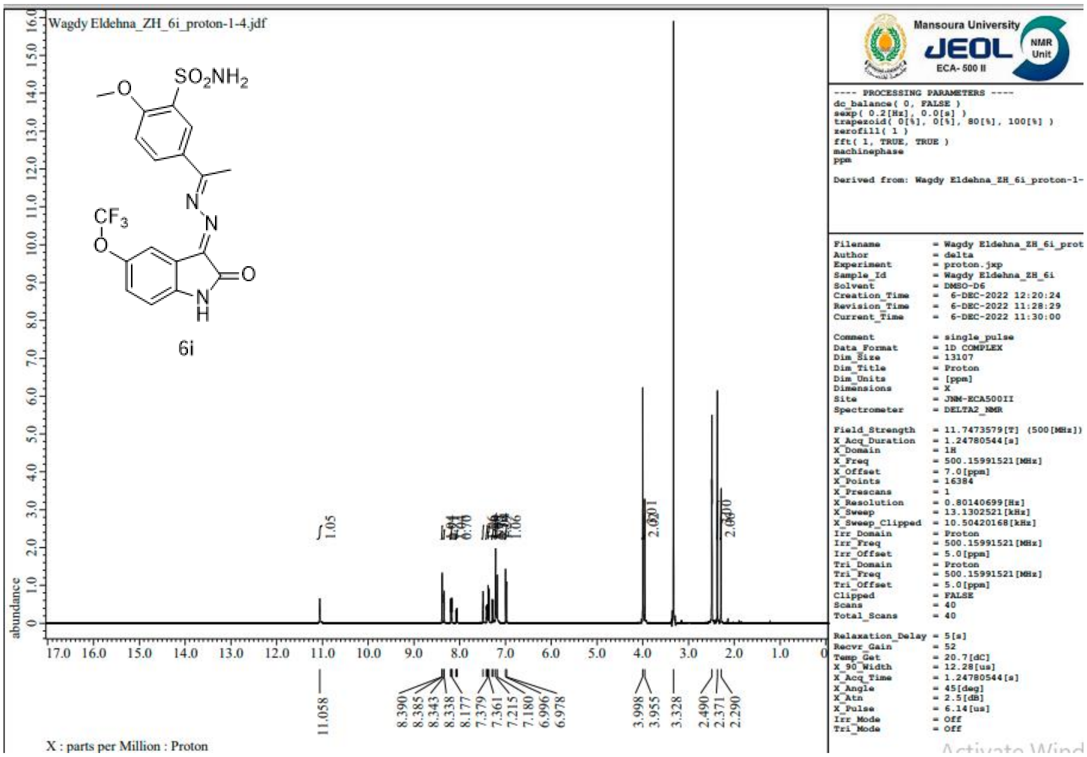

Figure S25. ^{1}H NMR (500 MHz, DMSO-*d6*) spectrum of compound **6i**

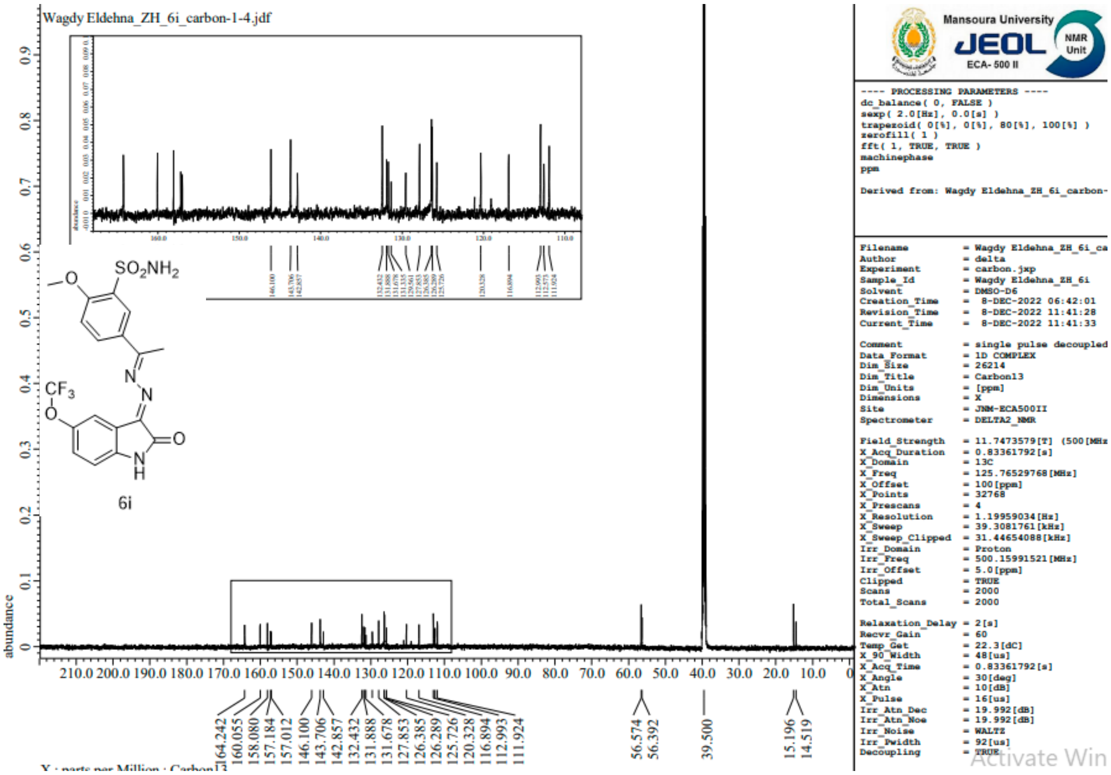

Figure S26. ^{13}C NMR (125 MHz, DMSO-*d6*) spectrum of compound **6i**

WalkUp FbF Workflow Report

UNIVERSITY OF CAMBRIDGE

Spectrum Peaks

m/z	m/z (Calc)	Diff (ppm)	Abund	Height %	Height % (Calc)	Ion Species	Z
457.0827	457.0788	8.52	402889	100.00	100.00	(M+H)+	1
458.0850	458.0816	7.29	89667	22.26	22.09	(M+H)+	1
459.0817	459.0786	6.70	30264	7.51	7.83	(M+H)+	1
460.0844	460.0801	9.28	5049	1.25	1.34	(M+H)+	1

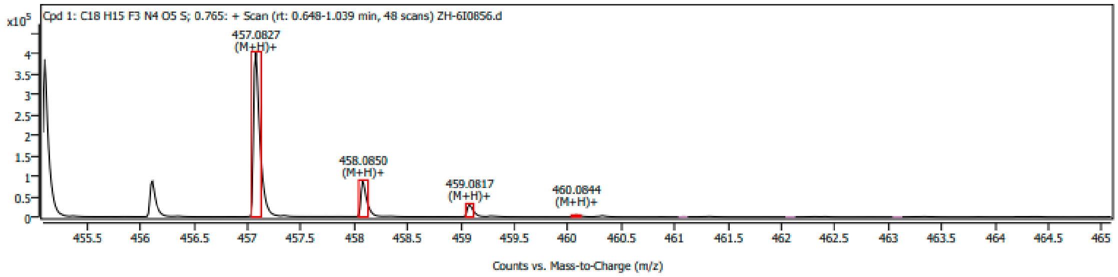

Spectrum Peaks

m/z	m/z (Calc)	Diff (ppm)	Abund	Height %	Height % (Calc)	Ion Species	Z
457.0827	457.0788	8.52	402889	100.00	100.00	(M+H)+	1
458.0850	458.0816	7.29	89667	22.26	22.09	(M+H)+	1
459.0817	459.0786	6.70	30264	7.51	7.83	(M+H)+	1
460.0844	460.0801	9.28	5049	1.25	1.34	(M+H)+	1

MassHunter Qual 10.0
(End of Report)

Figure S28. Mass spectrum of compound **6i**

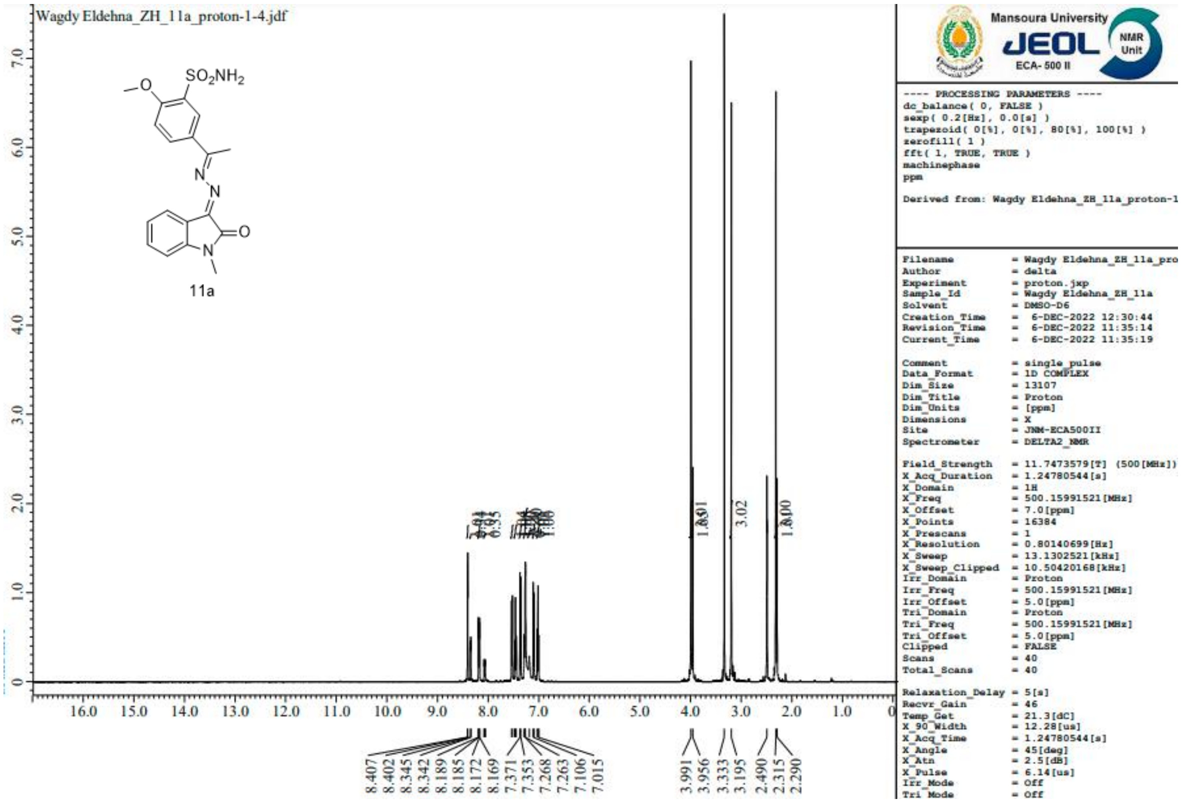

Figure S29. ^{1}H NMR (500 MHz, DMSO-*d6*) spectrum of compound **11a**

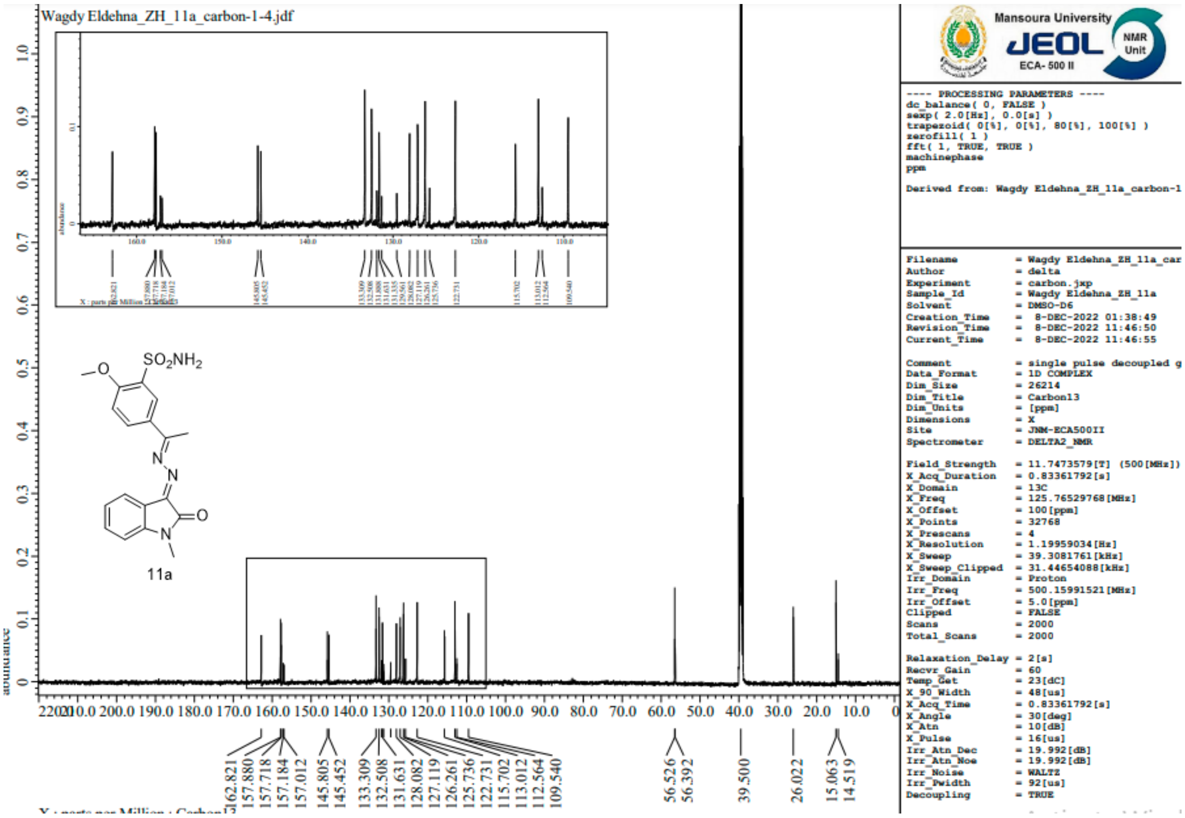

Figure S30. ^{13}C NMR (125 MHz, DMSO-*d6*) spectrum of compound **11a**

WalkUp FbF Workflow Report

UNIVERSITY OF CAMBRIDGE

Spectrum Peaks

m/z	m/z (Calc)	Diff (ppm)	Abund	Height %	Height % (Calc)	Ion Species	Z
387.1125	387.1122	0.89	1418141	100.00	100.00	(M+H)+	1
388.1160	388.1150	2.53	292609	20.63	22.09	(M+H)+	1
389.1129	389.1119	2.75	97667	6.89	7.62	(M+H)+	1
390.1134	390.1132	0.49	17400	1.23	1.29	(M+H)+	1

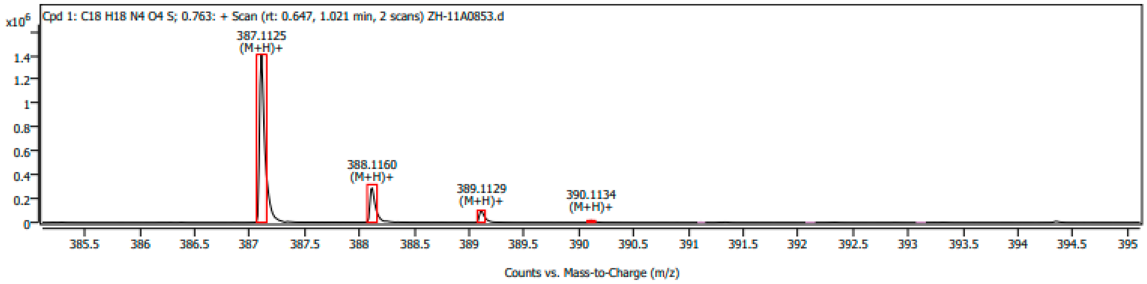

Spectrum Peaks

m/z	m/z (Calc)	Diff (ppm)	Abund	Height %	Height % (Calc)	Ion Species	Z
387.1125	387.1122	0.89	1418141	100.00	100.00	(M+H)+	1
388.1160	388.1150	2.53	292609	20.63	22.09	(M+H)+	1
389.1129	389.1119	2.75	97667	6.89	7.62	(M+H)+	1
390.1134	390.1132	0.49	17400	1.23	1.29	(M+H)+	1

MassHunter Qual 10.0
(End of Report)

Figure S31 . Mass spectrum of compound **11a**

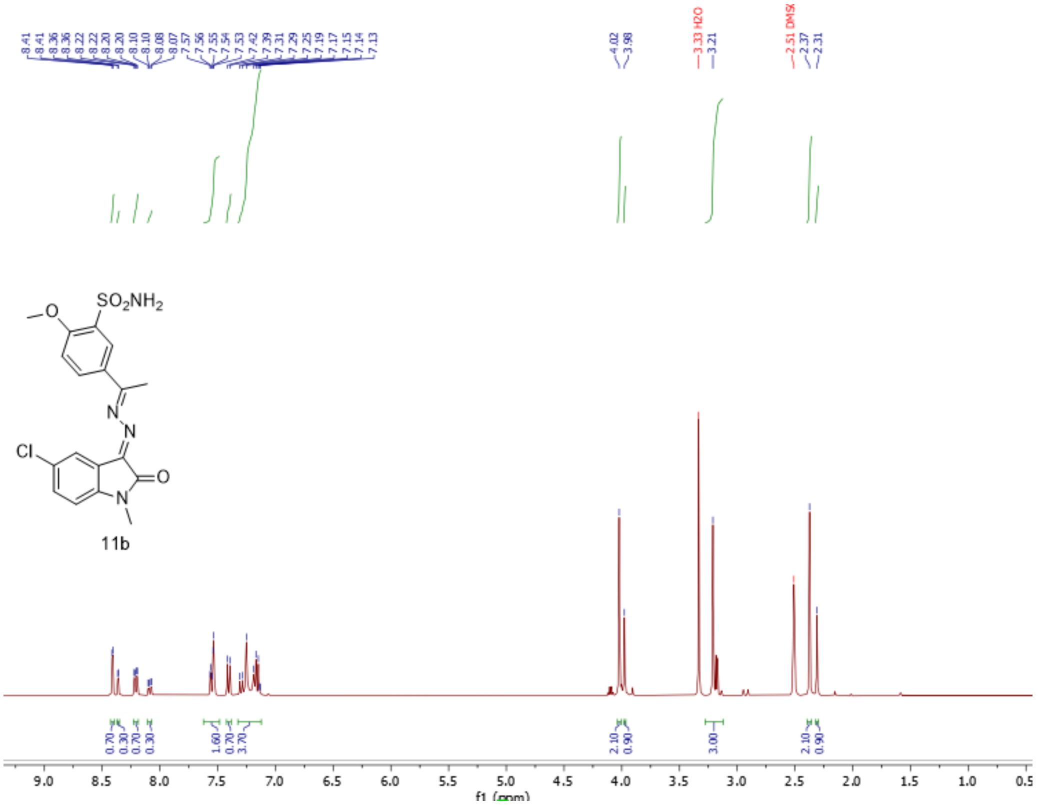

Figure S33. ^{1}H NMR (400 MHz, DMSO-*d6*) spectrum of compound **11b**

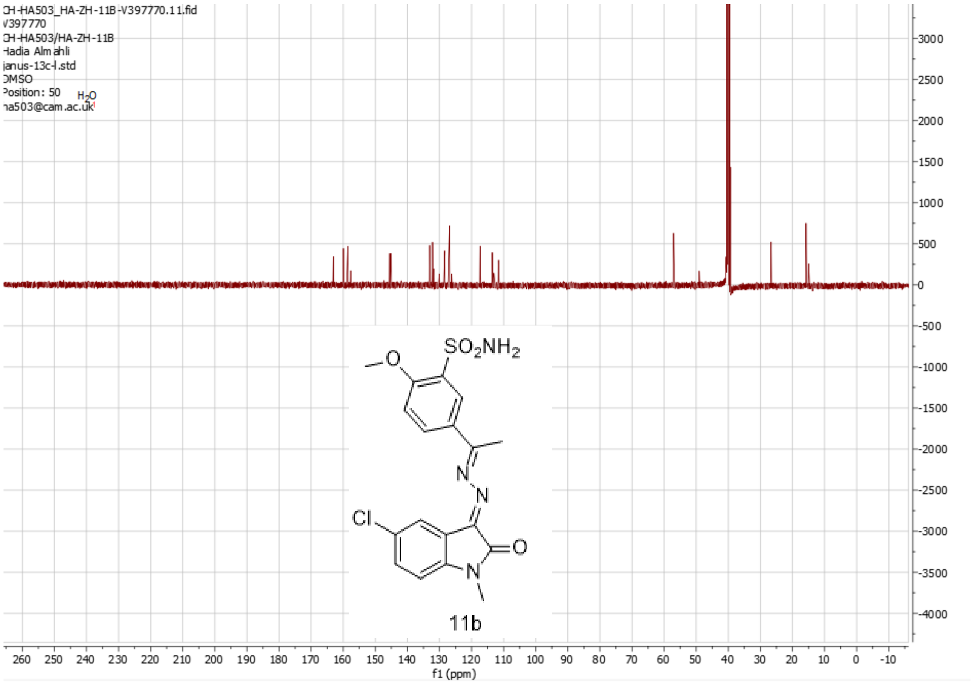

Figure S34. ^{13}C NMR (100 MHz, DMSO-*d6*) spectrum of compound **11b**

WalkUp FbF Workflow Report

UNIVERSITY OF CAMBRIDGE

Spectrum Peaks

m/z	m/z (Calc)	Diff (ppm)	Abund	Height %	Height % (Calc)	Ion Species	Z
421.0741	421.0732	2.28	629087	100.00	100.00	(M+H)+	1
422.0771	422.0760	2.52	137629	21.88	22.08	(M+H)+	1
423.0719	423.0707	2.75	244041	38.79	39.62	(M+H)+	1
424.0741	424.0732	1.92	51591	8.20	8.35	(M+H)+	1
425.0716	425.0703	3.00	15738	2.50	2.61	(M+H)+	1
426.0729	426.0716	3.06	2742	0.44	0.43	(M+H)+	1

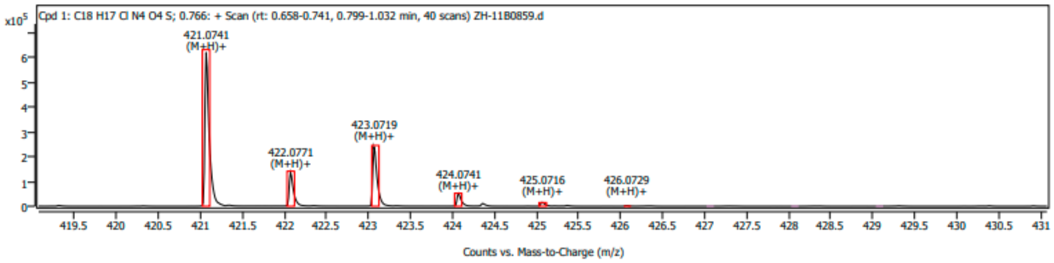

Spectrum Peaks

m/z	m/z (Calc)	Diff (ppm)	Abund	Height %	Height % (Calc)	Ion Species	Z
421.0741	421.0732	2.28	629087	100.00	100.00	(M+H)+	1
422.0771	422.0760	2.52	137629	21.88	22.08	(M+H)+	1
423.0719	423.0707	2.75	244041	38.79	39.62	(M+H)+	1
424.0741	424.0732	1.92	51591	8.20	8.35	(M+H)+	1
425.0716	425.0703	3.00	15738	2.50	2.61	(M+H)+	1
426.0729	426.0716	3.06	2742	0.44	0.43	(M+H)+	1

MassHunter Qual 10.0
(End of Report)

Figure S35. Mass spectrum of compound **11b**

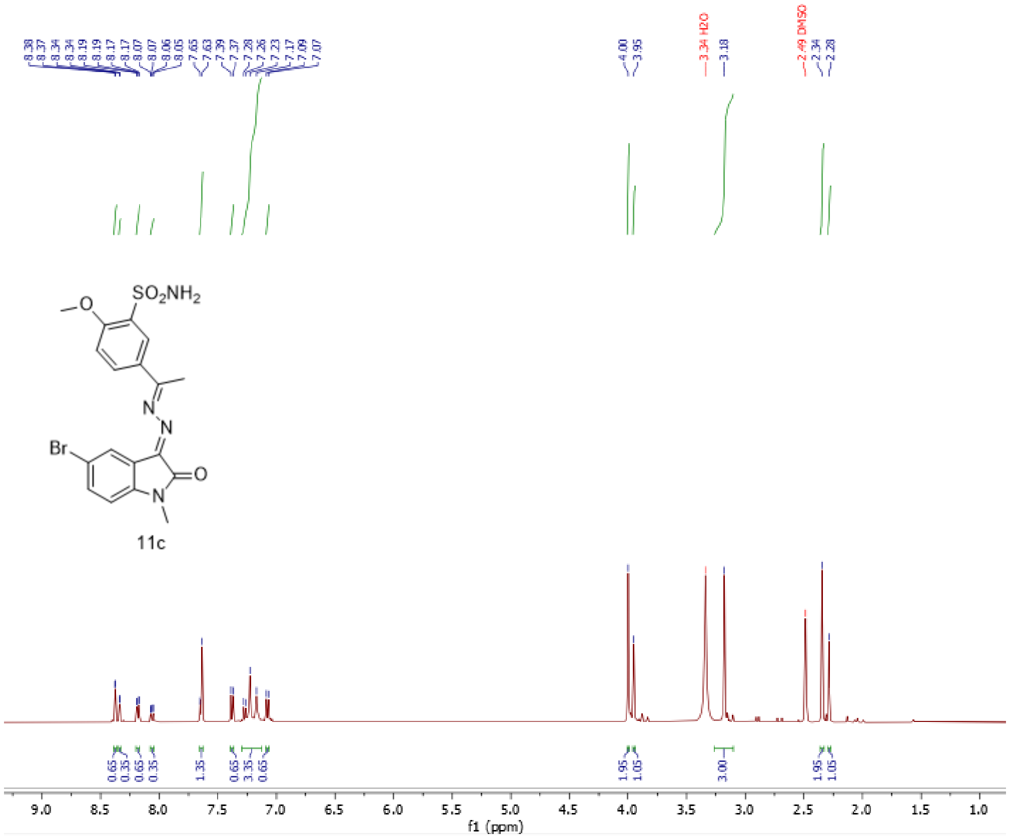

Figure S36. ^{1}H NMR (500 MHz, DMSO-*d6*) spectrum of compound **11c**

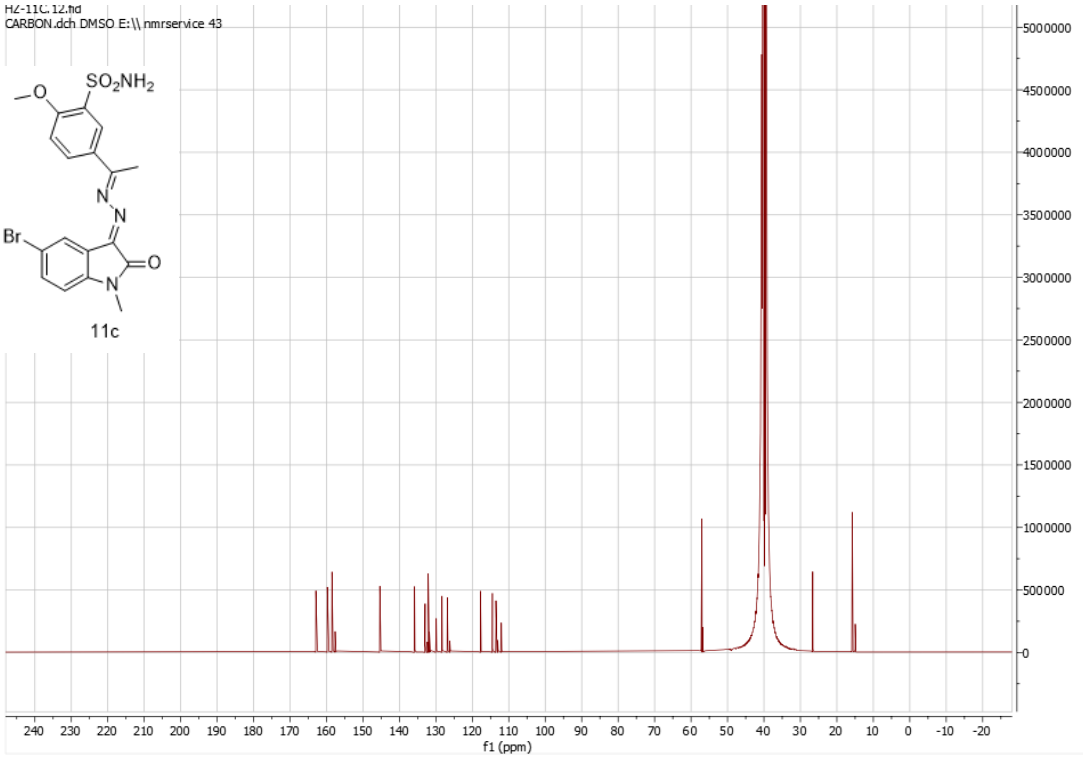

Figure S37. ^{13}C NMR (125 MHz, DMSO-*d6*) spectrum of compound **11c**

WalkUp FbF Workflow Report

UNIVERSITY OF CAMBRIDGE

Spectrum Peaks

m/z	m/z (Calc)	Diff (ppm)	Abund	Height %	Height % (Calc)	Ion Species	Z
465.0232	465.0227	1.11	84870	96.22	95.33	(M+H)+	1
466.0259	466.0255	0.87	18737	21.24	21.05	(M+H)+	1
467.0213	467.0207	1.18	88202	100.00	100.00	(M+H)+	1
468.0241	468.0235	1.37	18689	21.19	21.70	(M+H)+	1
469.0215	469.0204	2.24	6421	7.28	7.23	(M+H)+	1
470.0255	470.0218	7.89	1096	1.24	1.21	(M+H)+	1

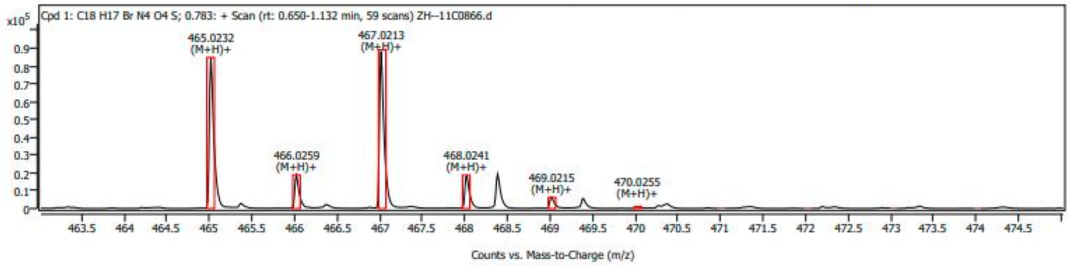

Spectrum Peaks

m/z	m/z (Calc)	Diff (ppm)	Abund	Height %	Height % (Calc)	Ion Species	Z
465.0232	465.0227	1.11	84870	96.22	95.33	(M+H)+	1
466.0259	466.0255	0.87	18737	21.24	21.05	(M+H)+	1
467.0213	467.0207	1.18	88202	100.00	100.00	(M+H)+	1
468.0241	468.0235	1.37	18689	21.19	21.70	(M+H)+	1
469.0215	469.0204	2.24	6421	7.28	7.23	(M+H)+	1
470.0255	470.0218	7.89	1096	1.24	1.21	(M+H)+	1

MassHunter Qual 10.0
(End of Report)

Figure S38. Mass spectrum of compound **11c**

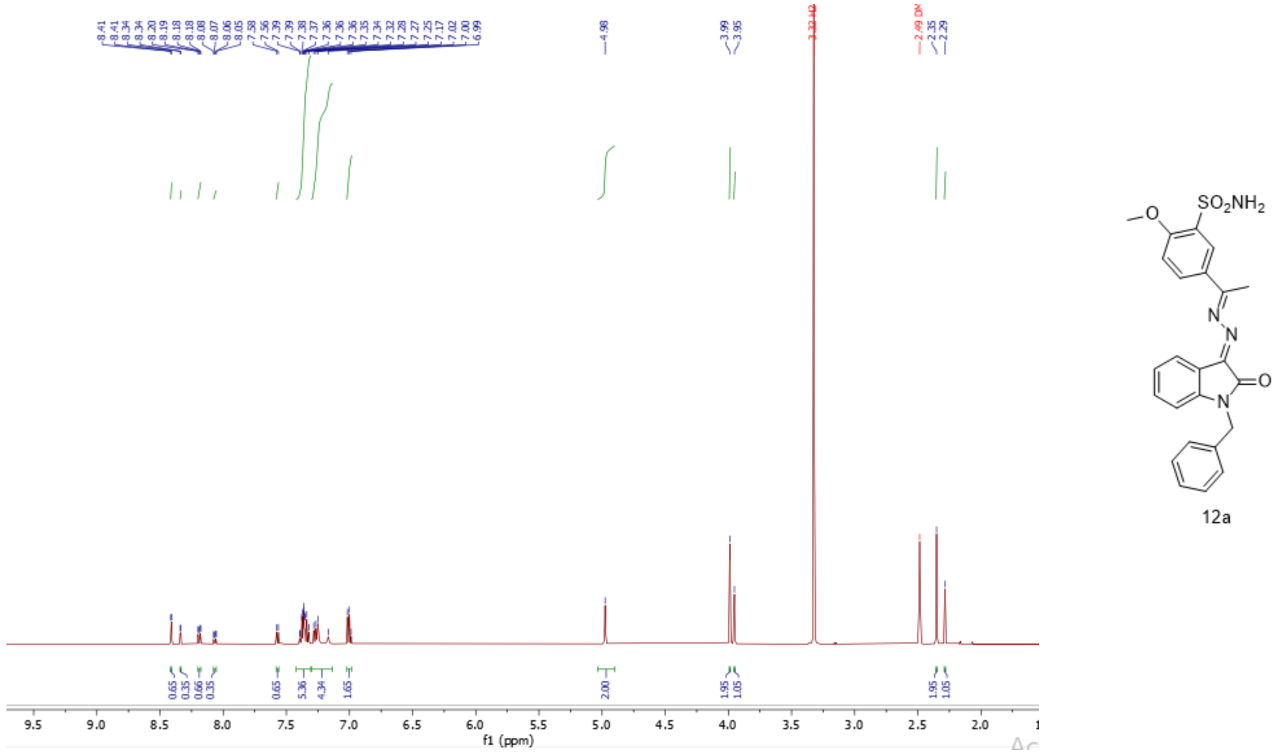

Figure S39. ^{1}H NMR (500 MHz, DMSO-*d6*) spectrum of compound **12a**

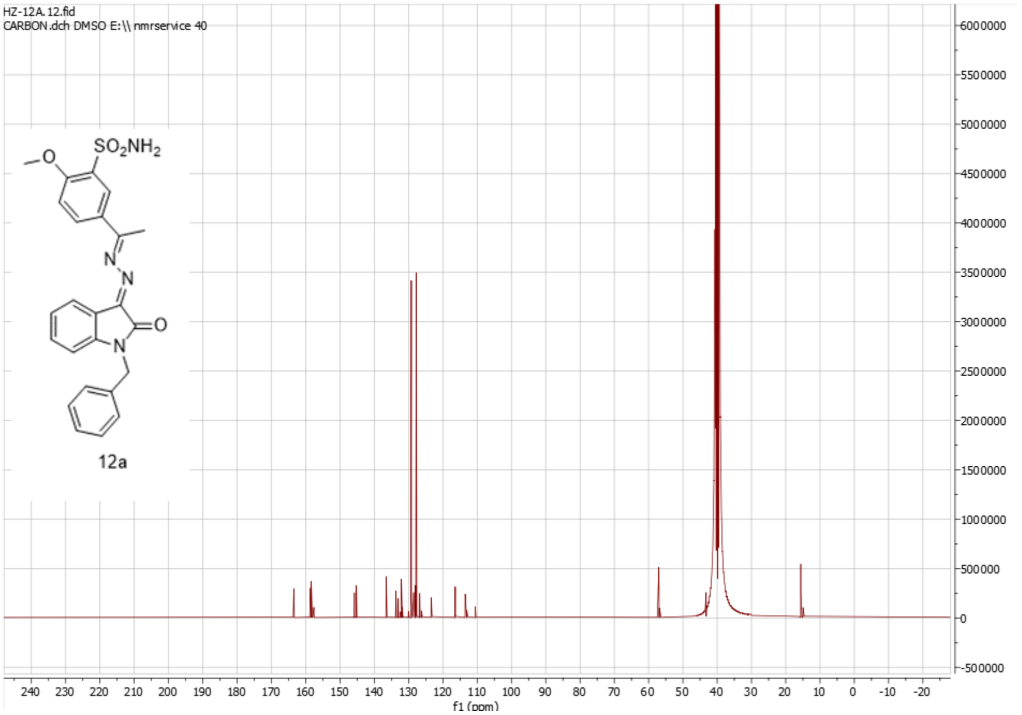

Figure S40. ^{13}C NMR (125 MHz, DMSO-*d6*) spectrum of compound **12a**

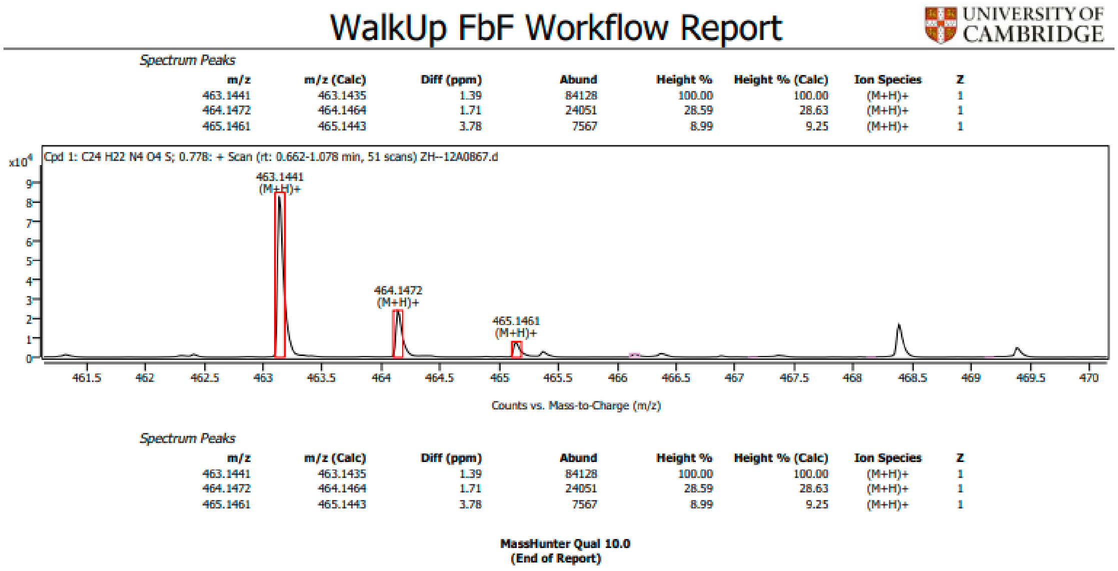

Figure S41. Mass spectrum of compound **12a**

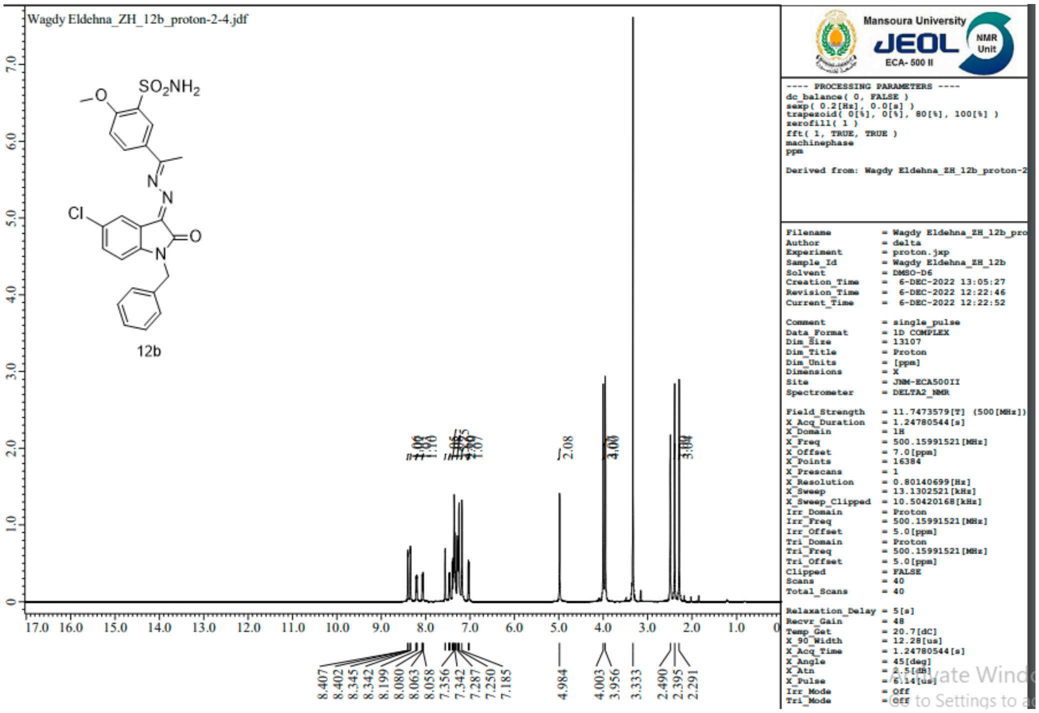

Figure S42. ^{1}H NMR (500 MHz, DMSO-*d6*) spectrum of compound **12b**

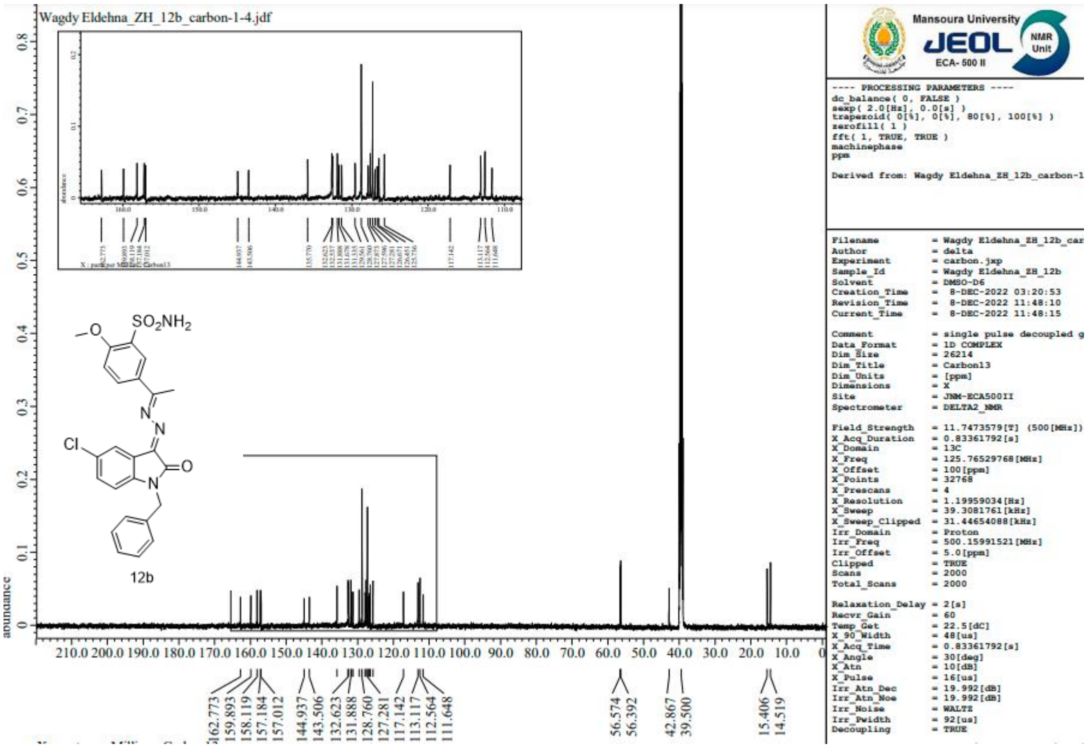

Figure S43. ^{13}C NMR (125 MHz, DMSO-*d6*) spectrum of compound **12b**

WalkUp FbF Workflow Report

UNIVERSITY OF CAMBRIDGE

Spectrum Peaks

m/z	m/z (Calc)	Diff (ppm)	Abund	Height %	Height % (Calc)	Ion Species	Z
497.1052	497.1045	1.44	444174	100.00	100.00	(M+H)+	1
498.1081	498.1074	1.37	127704	28.75	28.61	(M+H)+	1
499.1030	499.1024	1.28	183947	41.41	41.24	(M+H)+	1
500.1051	500.1048	0.56	49648	11.18	10.98	(M+H)+	1
501.1031	501.1028	0.62	14338	3.23	3.23	(M+H)+	1
502.1039	502.1037	0.40	2898	0.65	0.62	(M+H)+	1

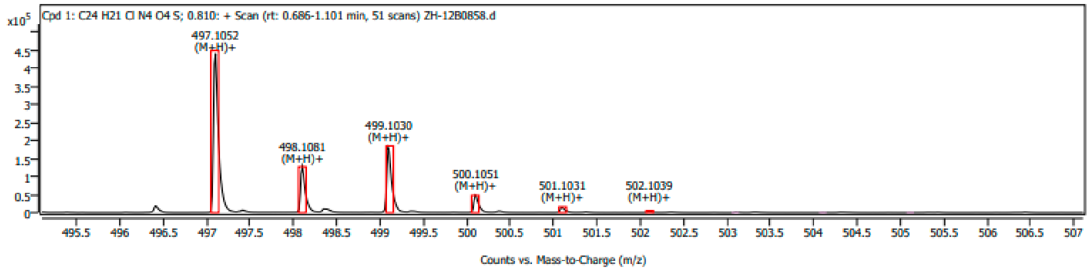

Spectrum Peaks

m/z	m/z (Calc)	Diff (ppm)	Abund	Height %	Height % (Calc)	Ion Species	Z
497.1052	497.1045	1.44	444174	100.00	100.00	(M+H)+	1
498.1081	498.1074	1.37	127704	28.75	28.61	(M+H)+	1
499.1030	499.1024	1.28	183947	41.41	41.24	(M+H)+	1
500.1051	500.1048	0.56	49648	11.18	10.98	(M+H)+	1
501.1031	501.1028	0.62	14338	3.23	3.23	(M+H)+	1
502.1039	502.1037	0.40	2898	0.65	0.62	(M+H)+	1

MassHunter Qual 10.0
(End of Report)

Figure S44. Mass spectrum of compound **12b**

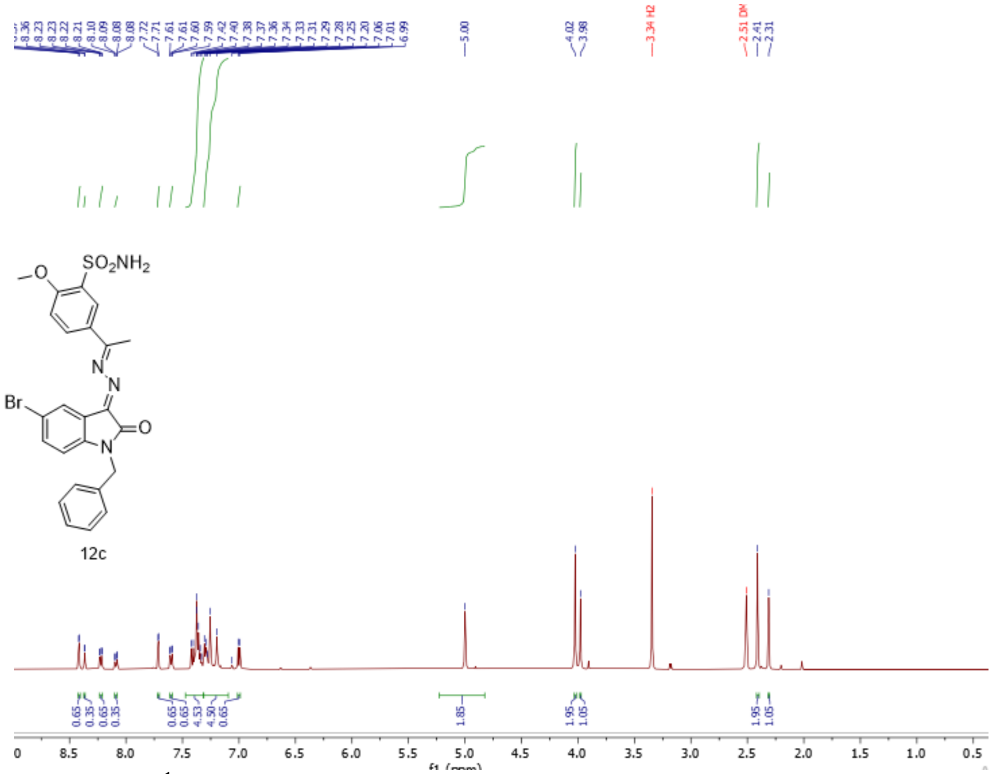

Figure S45. ^{1}H NMR (500 MHz, DMSO-*d6*) spectrum of compound **12c**

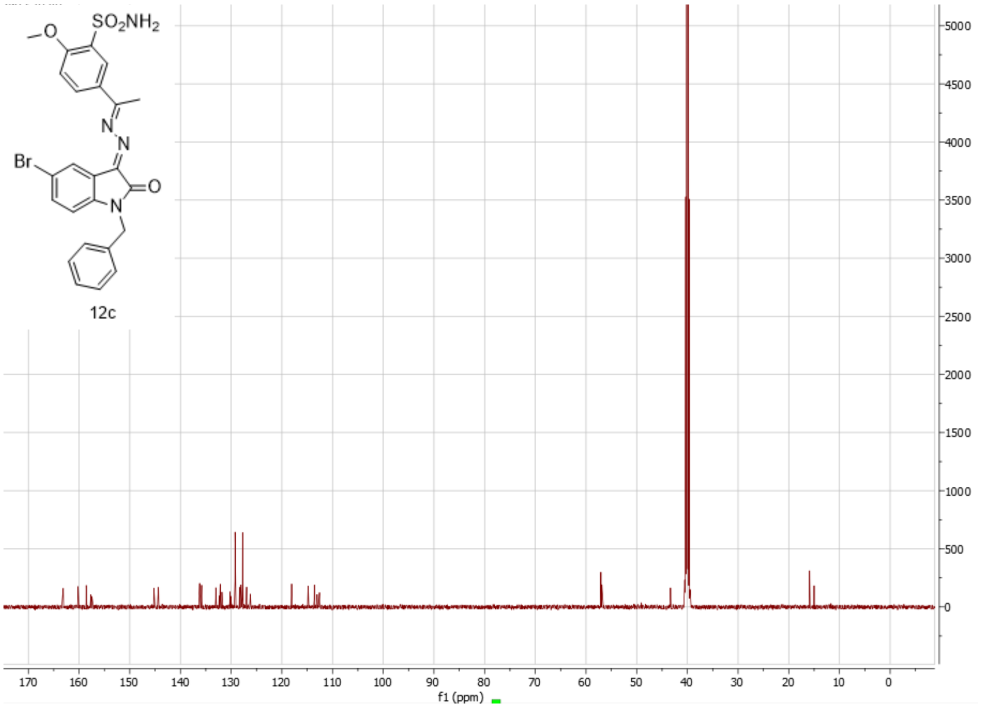

Figure S46. ^{13}C NMR (125 MHz, DMSO-*d6*) spectrum of compound **12c**

WalkUp FbF Workflow Report

UNIVERSITY OF CAMBRIDGE

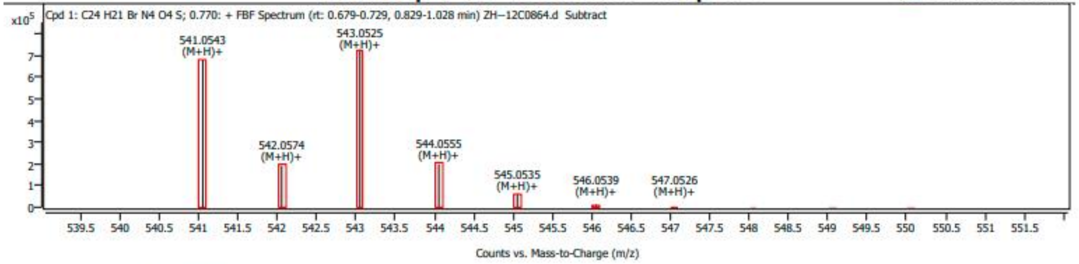

Spectrum Peaks

m/z	m/z (Calc)	Diff (ppm)	Abund	Height %	Height % (Calc)	Ion Species	Z
541.0543	541.0540	0.58	680547	93.35	93.88	(M+H)+	1
542.0574	542.0569	0.95	192425	26.39	26.86	(M+H)+	1
543.0525	543.0522	0.54	729034	100.00	100.00	(M+H)+	1
544.0555	544.0549	1.05	199333	27.34	27.85	(M+H)+	1
545.0535	545.0529	1.02	61570	8.45	8.70	(M+H)+	1
546.0539	546.0538	0.18	11976	1.64	1.70	(M+H)+	1
547.0526	547.0552	-4.75	2664	0.37	0.25	(M+H)+	1

Figure S47. Mass spectrum of compound **12c**

Requester Data

Name:	**Haytham O. Tawfik**	Tel.	**01005356819**
Authority:	**Faculty of Pharmacy Tanta University**	Date	**24/11/2022**

Sample Data

Samples had been submitted for elemental analysis.

Analysis Report

No.	Code	C%	H%	N%	S%
1	**6a**	55.09	4.25	14.95	8.69
2	**6b**	52.11	3.95	14.50	8.18
3	**6c**	52.08	3.85	14.23	8.15
4	**6d**	49.95	3.75	13.90	8.00
5	**6e**	50.40	3.69	13.65	7.80
6	**6f**	45.02	3.30	12.25	7.08
7	**6g**	45.06	3.29	12.52	7.14
8	**6h**	53.99	4.45	14.02	7.93
9	**6i**	47.60	3.33	12.17	7.07
10	**11a**	56.20	4.75	14.39	8.21
11	**11b**	51.11	4.04	13.20	7.55
12	**11c**	46.69	3.72	12.19	6.99
13	**12a**	62.00	4.78	12.03	7.00
14	**12b**	57.75	4.28	11.18	6.50
15	**12c**	53.45	3.90	10.27	5.95

INVESTIGATOR — M. Abd-Elsattar

DIRECTOR — H. Omar

Tel. :0403336007 – 0403336409 (Int. 456)

Email: central.research.lab@pharm.tanta.edu.eg

Address: Faculty of pharmacy, Medical campus, Elgeish Street, Tanta

الكلية معتمدة من الهيئة القومية لضمان جودة التعليم والإعتماد

Figure S48. Elemental analysis of target compounds **6a-i, 11a-c,** and **12a-c**

Developmental Therapeutics Program	NSC: D-837819 / 1	Conc: 1.00E-5 Molar	Test Date: May 02, 2022
One Dose Mean Graph	Experiment ID: 2205OS11		Report Date: Jul 14, 2022

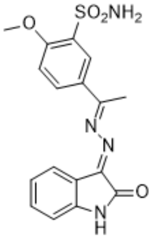

6a

Panel/Cell Line	Growth Percent
Leukemia	
CCRF-CEM	86.72
HL-60(TB)	99.81
K-562	93.50
MOLT-4	93.00
RPMI-8226	94.13
SR	80.50
Non-Small Cell Lung Cancer	
A549/ATCC	95.52
EKVX	91.86
HOP-62	92.64
HOP-92	97.31
NCI-H226	83.86
NCI-H23	95.60
NCI-H322M	95.89
NCI-H460	101.32
NCI-H522	93.51
Colon Cancer	
COLO 205	107.04
HCC-2998	107.73
HCT-116	90.77
HCT-15	98.83
HT29	95.64
KM12	99.89
SW-620	100.52
CNS Cancer	
SF-268	111.32
SF-295	89.59
SF-539	96.26
SNB-75	91.33
U251	85.79
Melanoma	
LOX IMVI	98.67
MALME-3M	102.69
M14	93.61
MDA-MB-435	104.64
SK-MEL-2	95.42
SK-MEL-28	100.85
SK-MEL-5	90.12
UACC-257	97.03
UACC-62	88.01
Ovarian Cancer	
IGROV1	107.19
OVCAR-3	112.02
OVCAR-4	102.55
OVCAR-5	94.79
OVCAR-8	99.31
NCI/ADR-RES	102.37
SK-OV-3	101.53
Renal Cancer	
786-0	93.61
A498	98.44
ACHN	100.74
CAKI-1	90.64
RXF 393	85.81
TK-10	92.53
UO-31	92.69
Prostate Cancer	
PC-3	92.26
DU-145	106.89
Breast Cancer	
MCF7	88.21
MDA-MB-231/ATCC	90.18
HS 578T	93.58
BT-549	81.96
T-47D	92.21
MDA-MB-468	83.86
Mean	95.59
Delta	15.09
Range	31.52

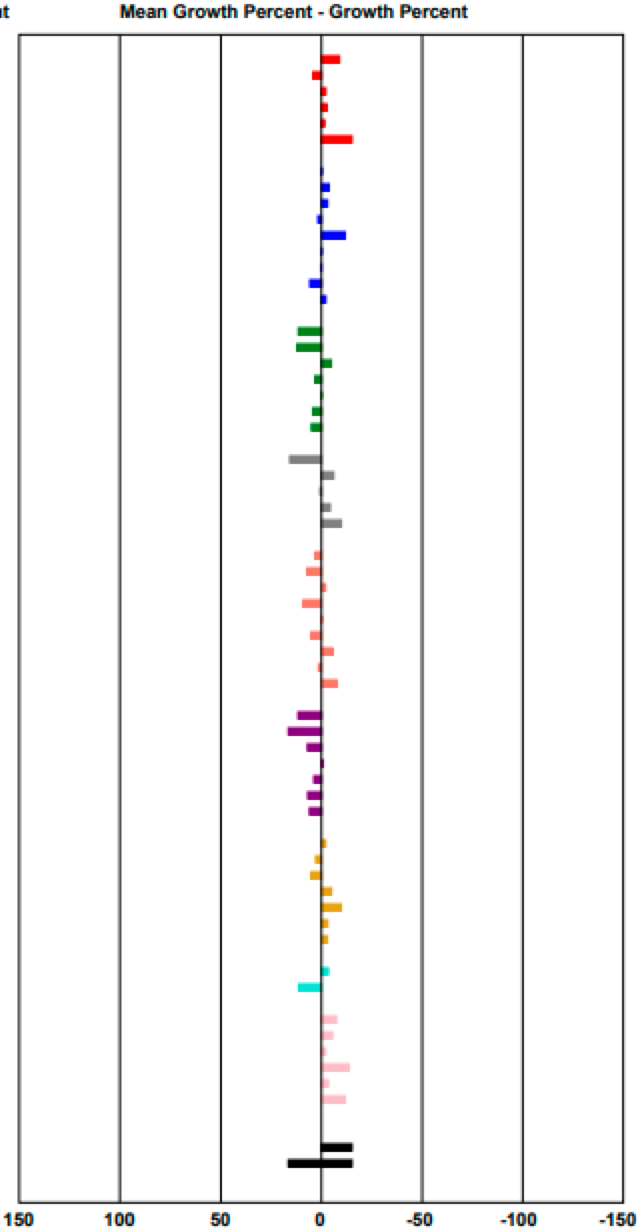

Figure S49. One dose mean graph for compound **6a** at 10 µM

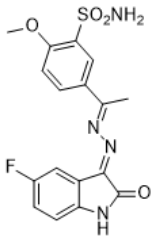

6b

Developmental Therapeutics Program	NSC: D-837820 / 1	Conc: 1.00E-5 Molar	Test Date: May 02, 2022
One Dose Mean Graph	Experiment ID: 2205OS11		Report Date: Jul 14, 2022

Panel/Cell Line	Growth Percent
Leukemia	
CCRF-CEM	87.34
HL-60(TB)	90.00
K-562	99.04
MOLT-4	89.32
RPMI-8226	87.21
SR	81.29
Non-Small Cell Lung Cancer	
A549/ATCC	97.58
EKVX	90.71
HOP-62	86.21
HOP-92	83.13
NCI-H226	76.34
NCI-H23	96.05
NCI-H322M	93.25
NCI-H460	102.25
NCI-H522	88.91
Colon Cancer	
COLO 205	114.46
HCC-2998	113.59
HCT-116	95.38
HCT-15	98.12
HT29	93.62
KM12	101.63
SW-620	103.72
CNS Cancer	
SF-268	96.95
SF-295	89.18
SF-539	92.83
SNB-75	88.08
U251	88.56
Melanoma	
LOX IMVI	94.43
MALME-3M	99.66
M14	97.70
MDA-MB-435	105.06
SK-MEL-2	97.53
SK-MEL-28	100.22
SK-MEL-5	88.58
UACC-257	99.86
UACC-62	86.83
Ovarian Cancer	
IGROV1	106.05
OVCAR-3	108.32
OVCAR-4	107.38
OVCAR-5	93.72
OVCAR-8	94.91
NCI/ADR-RES	99.89
SK-OV-3	105.25
Renal Cancer	
786-0	99.03
A498	108.48
ACHN	99.31
CAKI-1	83.27
RXF 393	90.37
TK-10	112.83
UO-31	93.61
Prostate Cancer	
PC-3	94.00
DU-145	107.02
Breast Cancer	
MCF7	85.59
MDA-MB-231/ATCC	84.17
HS 578T	89.33
BT-549	83.71
T-47D	84.96
MDA-MB-468	75.73
Mean	94.85
Delta	19.12
Range	38.73

Mean Growth Percent - Growth Percent

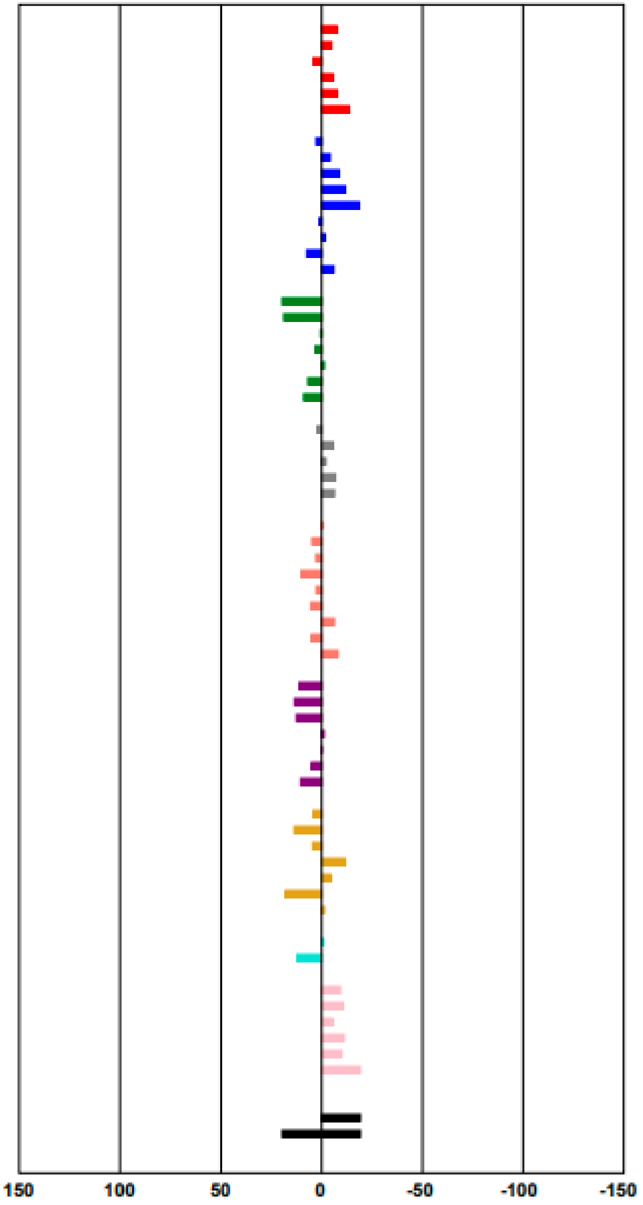

Figure S50. One dose mean graph for compound **6b** at 10 μM

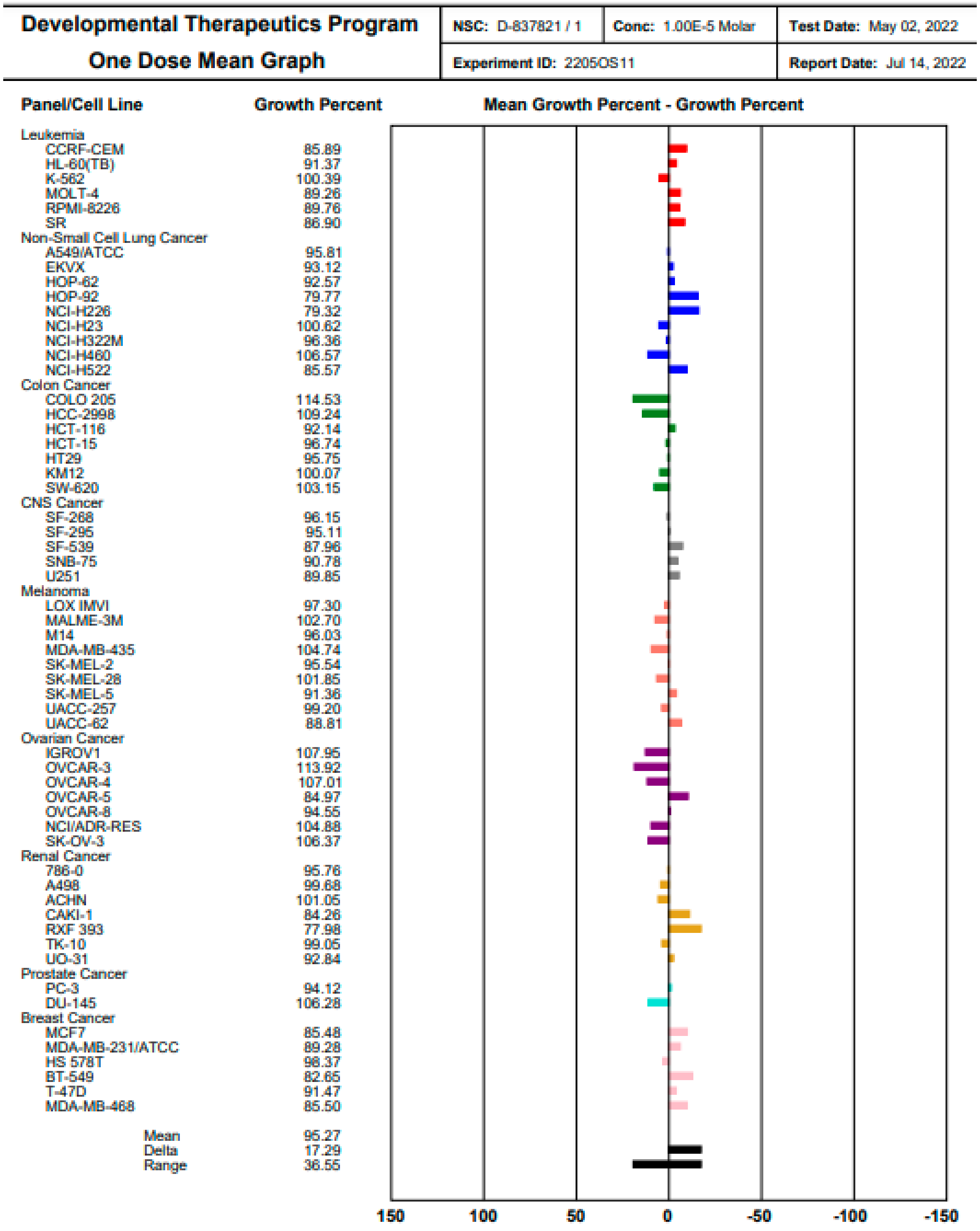

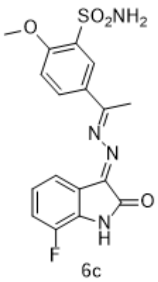

6c

Figure S51. One dose mean graph for compound **6c** at 10 μM

Developmental Therapeutics Program One Dose Mean Graph	NSC: D-837822 / 1	Conc: 1.00E-5 Molar	Test Date: May 02, 2022
	Experiment ID: 2205OS11		Report Date: Jul 14, 2022

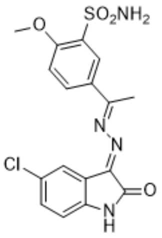

6d

Panel/Cell Line	Growth Percent
**Leukemia**	
CCRF-CEM	79.04
HL-60(TB)	89.75
K-562	95.60
MOLT-4	82.39
RPMI-8226	81.88
SR	81.10
**Non-Small Cell Lung Cancer**	
A549/ATCC	97.28
EKVX	91.69
HOP-62	83.00
HOP-92	78.50
NCI-H226	78.45
NCI-H23	95.25
NCI-H322M	81.98
NCI-H460	92.54
NCI-H522	66.23
**Colon Cancer**	
COLO 205	105.37
HCC-2998	111.70
HCT-116	83.29
HCT-15	96.20
HT29	98.14
KM12	100.11
SW-620	92.66
**CNS Cancer**	
SF-268	91.46
SF-295	90.91
SF-539	83.50
SNB-75	92.13
U251	86.44
**Melanoma**	
LOX IMVI	89.87
MALME-3M	95.71
M14	87.28
MDA-MB-435	105.91
SK-MEL-2	91.05
SK-MEL-28	103.54
SK-MEL-5	85.18
UACC-257	98.69
UACC-62	78.31
**Ovarian Cancer**	
IGROV1	104.17
OVCAR-3	107.71
OVCAR-4	102.18
OVCAR-5	109.45
OVCAR-8	90.57
NCI/ADR-RES	98.19
SK-OV-3	94.94
**Renal Cancer**	
786-0	95.45
A498	94.19
ACHN	95.78
CAKI-1	72.74
RXF 393	88.73
TK-10	106.45
UO-31	94.00
**Prostate Cancer**	
PC-3	90.32
DU-145	95.57
**Breast Cancer**	
MCF7	86.25
MDA-MB-231/ATCC	77.07
HS 578T	67.75
BT-549	78.65
T-47D	75.82
MDA-MB-468	77.54
Mean	90.44
Delta	24.21
Range	45.47

Mean Growth Percent - Growth Percent

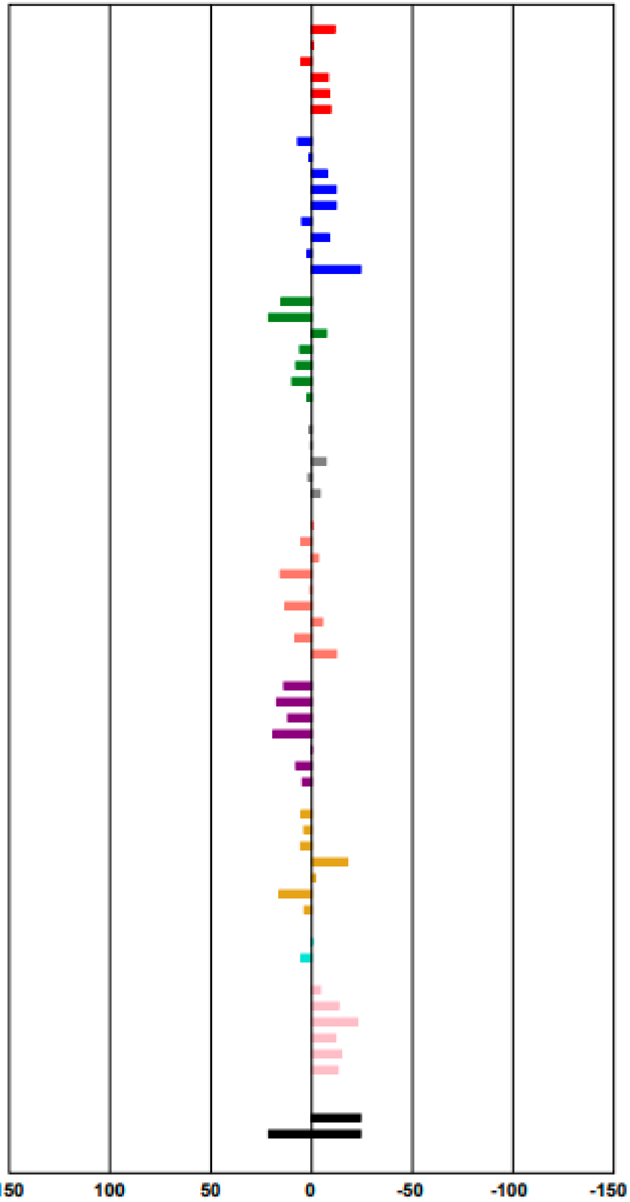

Figure S52. One dose mean graph for compound **6d** at 10 μM

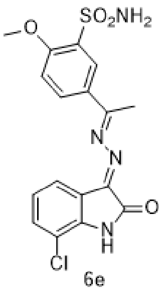

6e

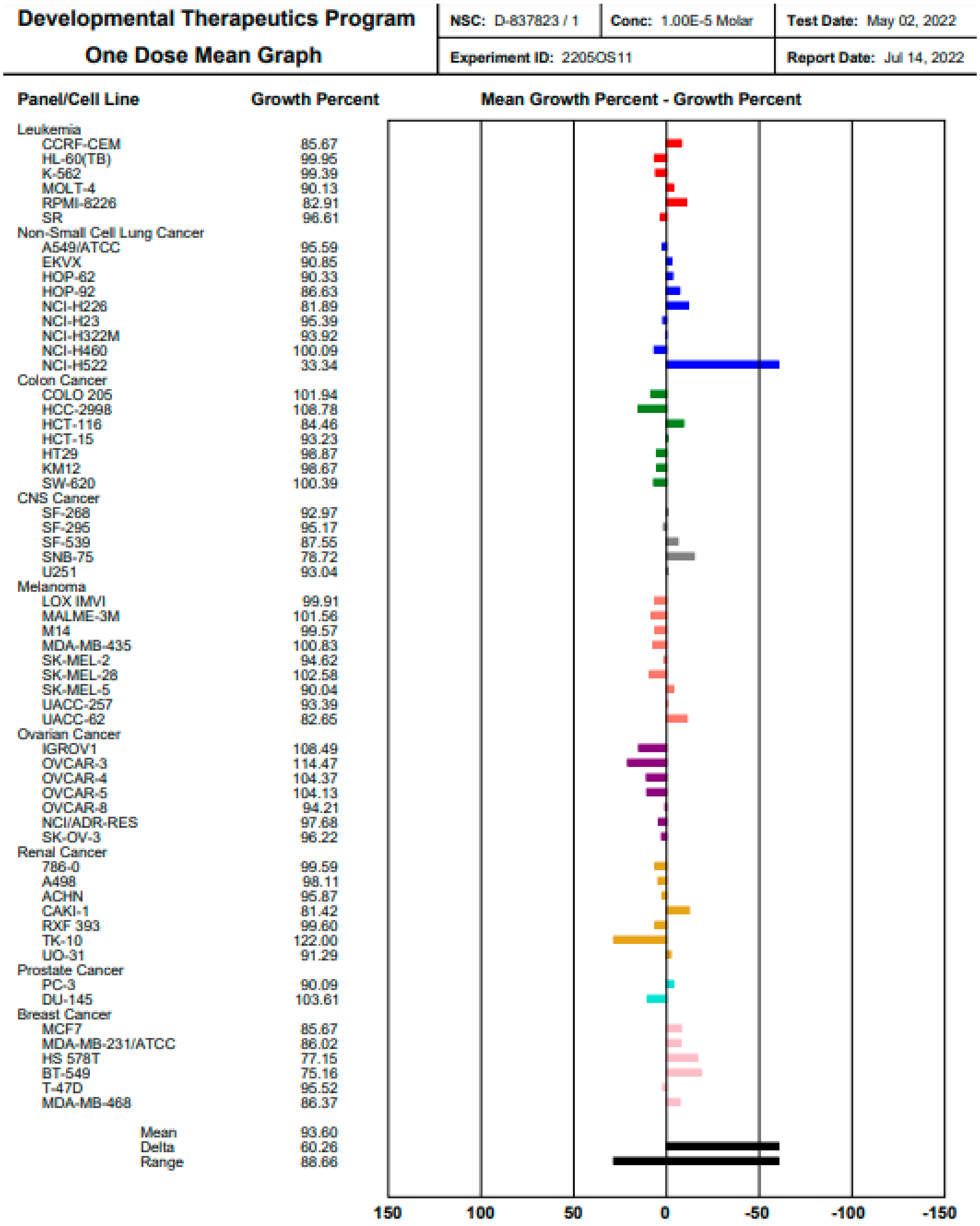

Figure S53. One dose mean graph for compound **6e** at 10 µM

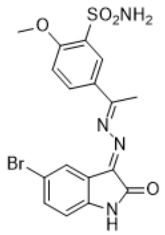

6f

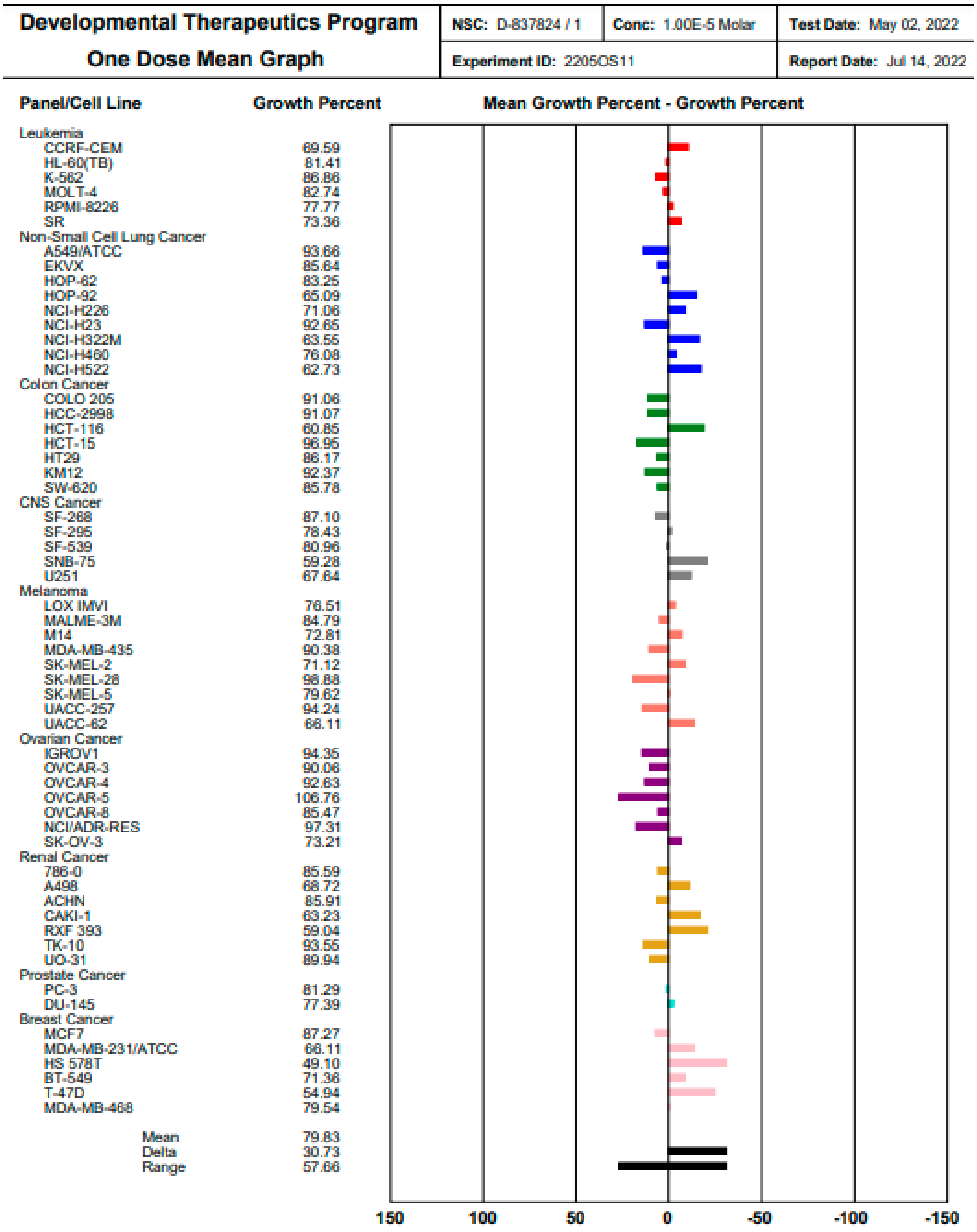

Figure S54. One dose mean graph for compound **6f** at 10 μM

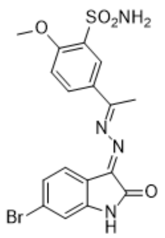

6g

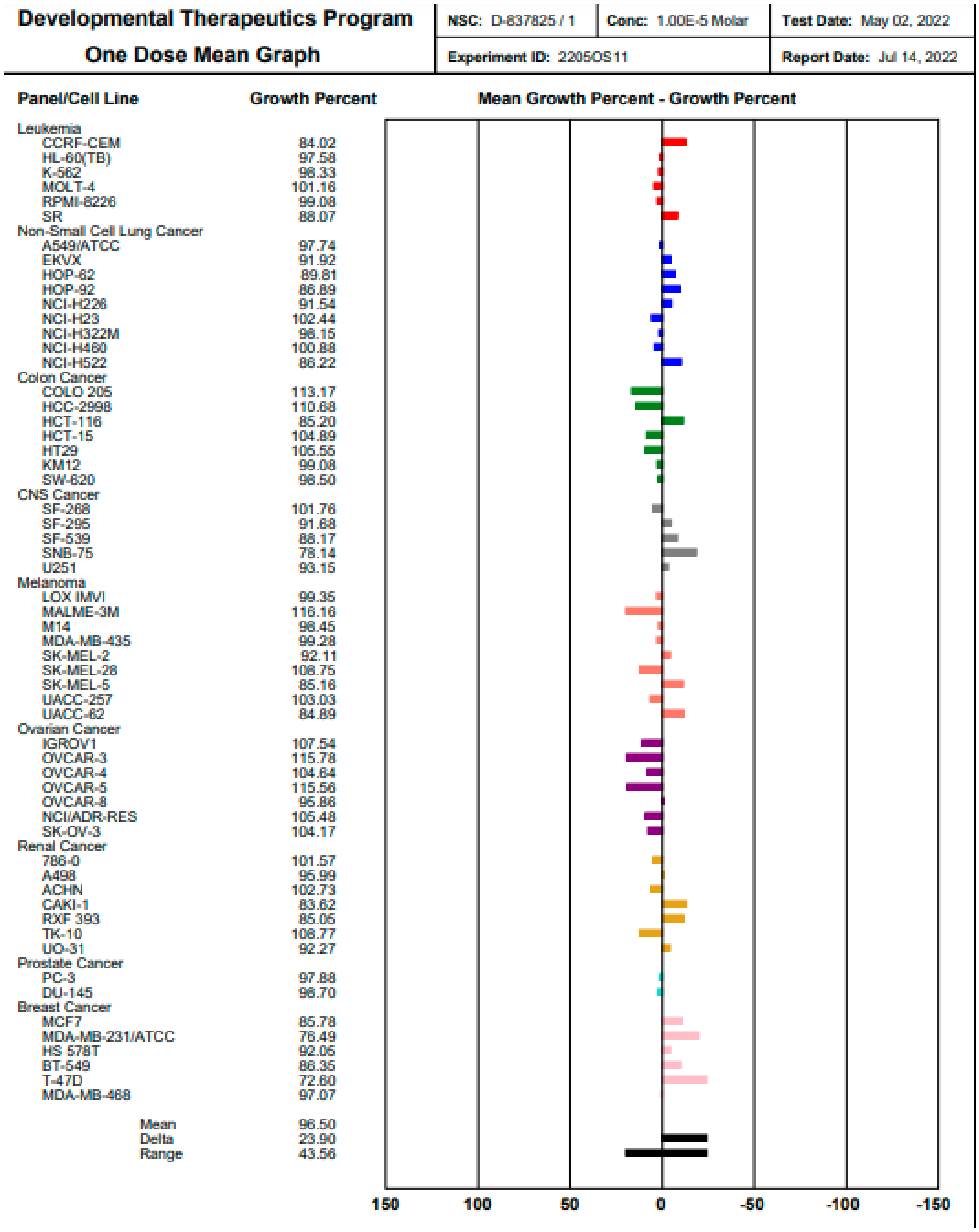

Figure S55. One dose mean graph for compound **6g** at 10 μM

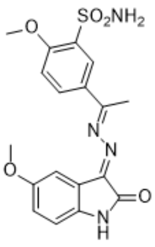

6h

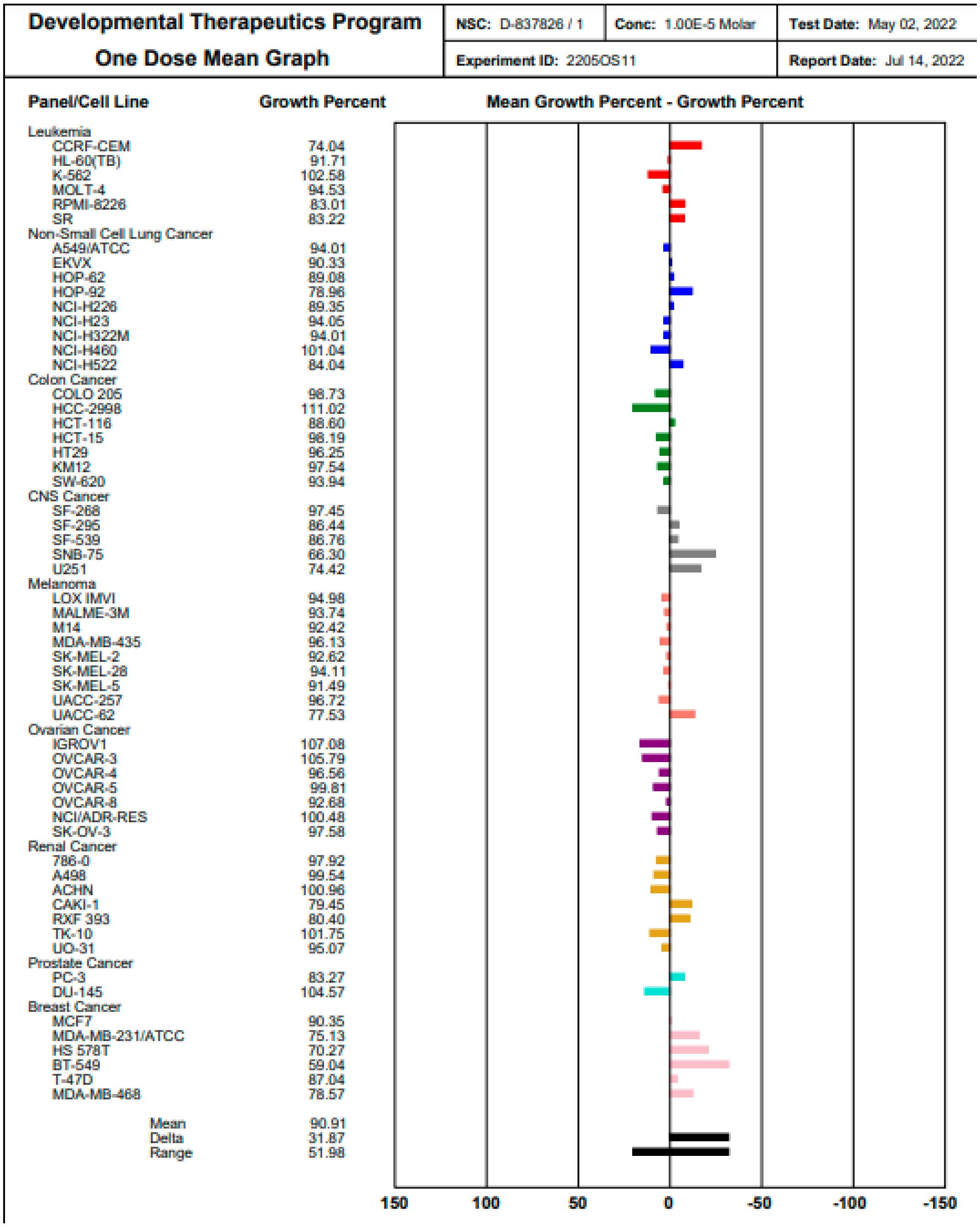

Figure S56. One dose mean graph for compound **6h** at 10 μM

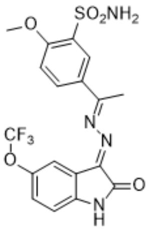

6i

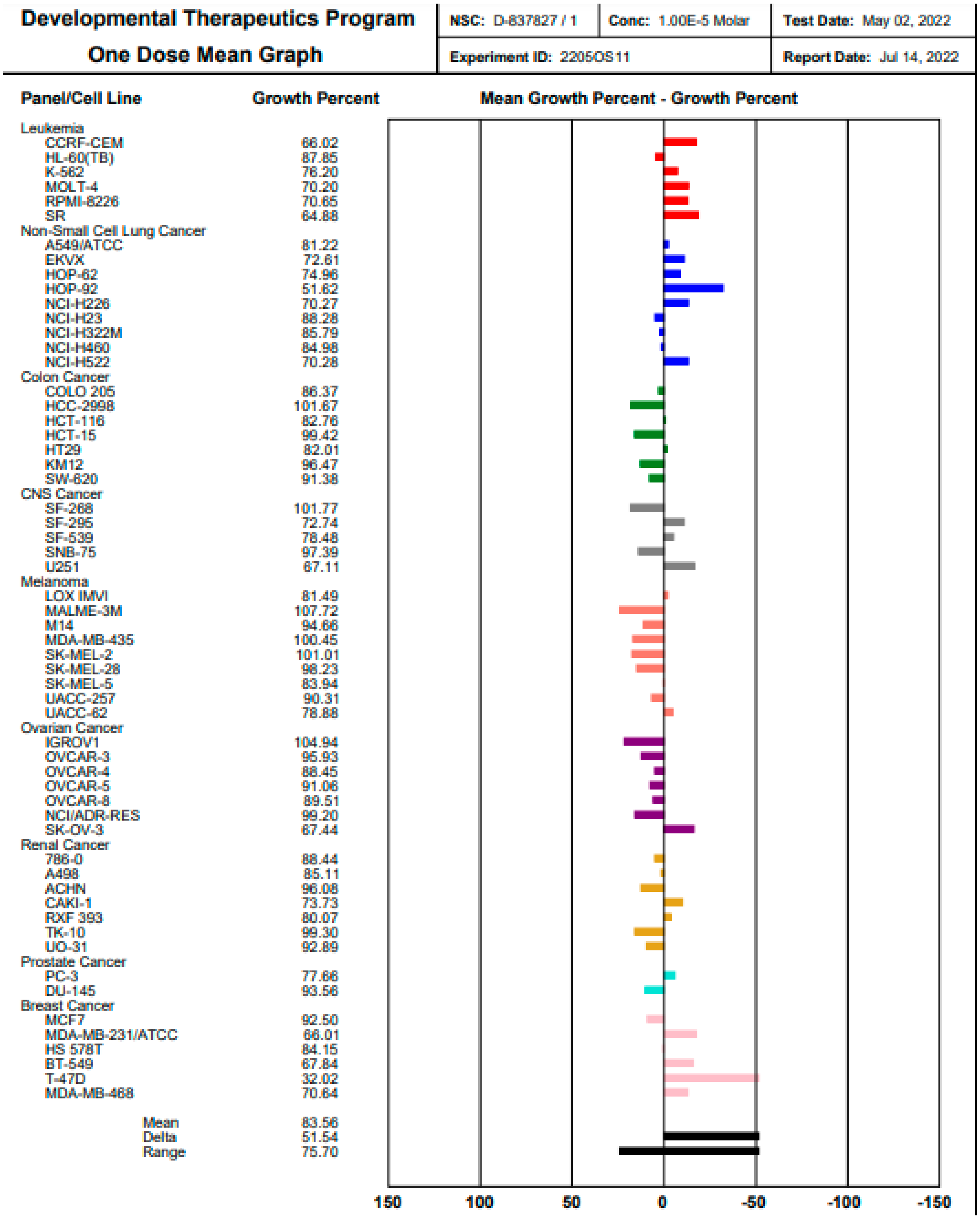

Figure S57. One dose mean graph for compound **6i** at 10 µM

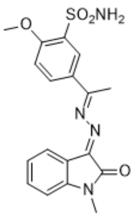

11a

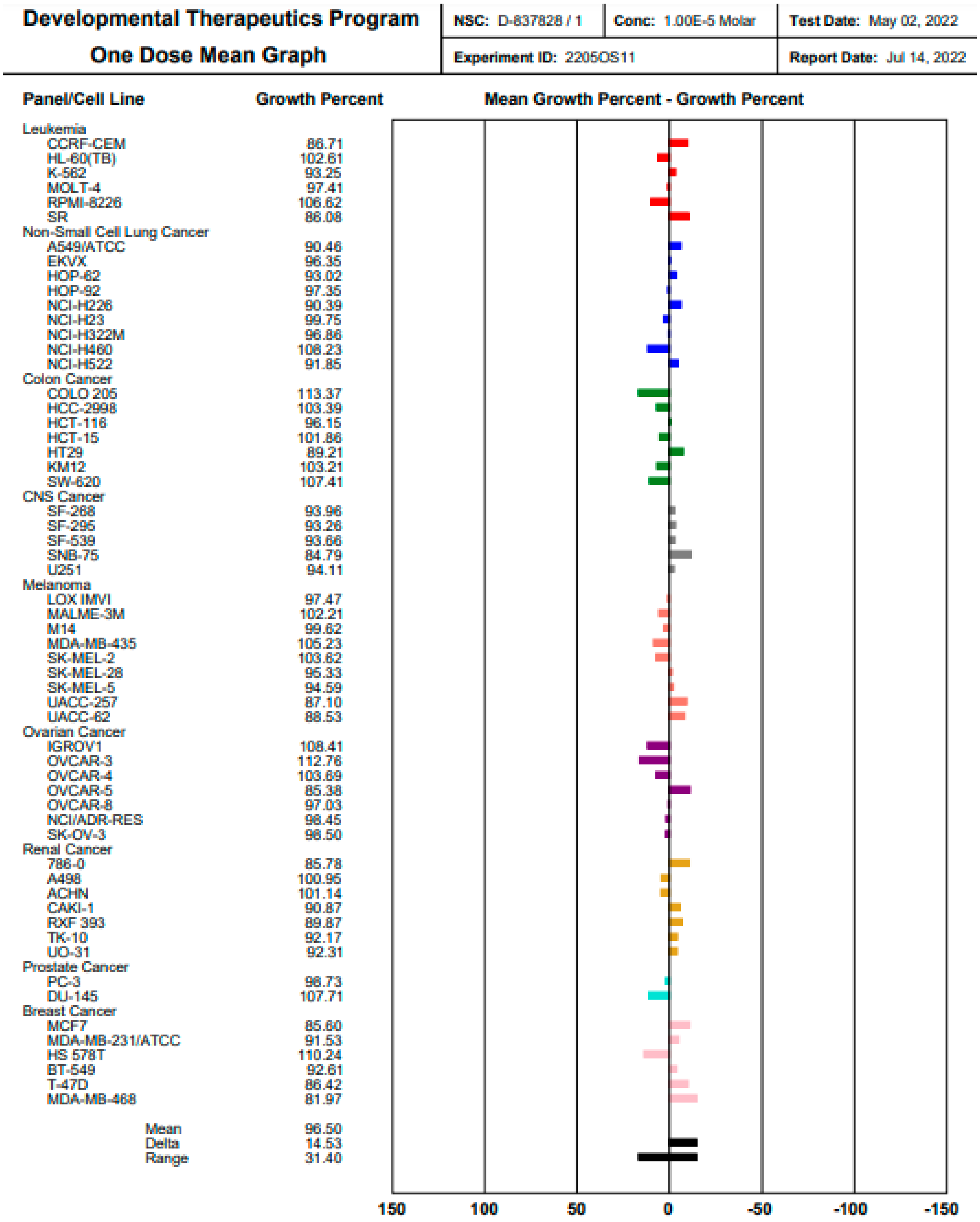

Figure S58. One dose mean graph for compound **11a** at 10 μM

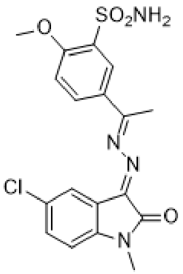

11b

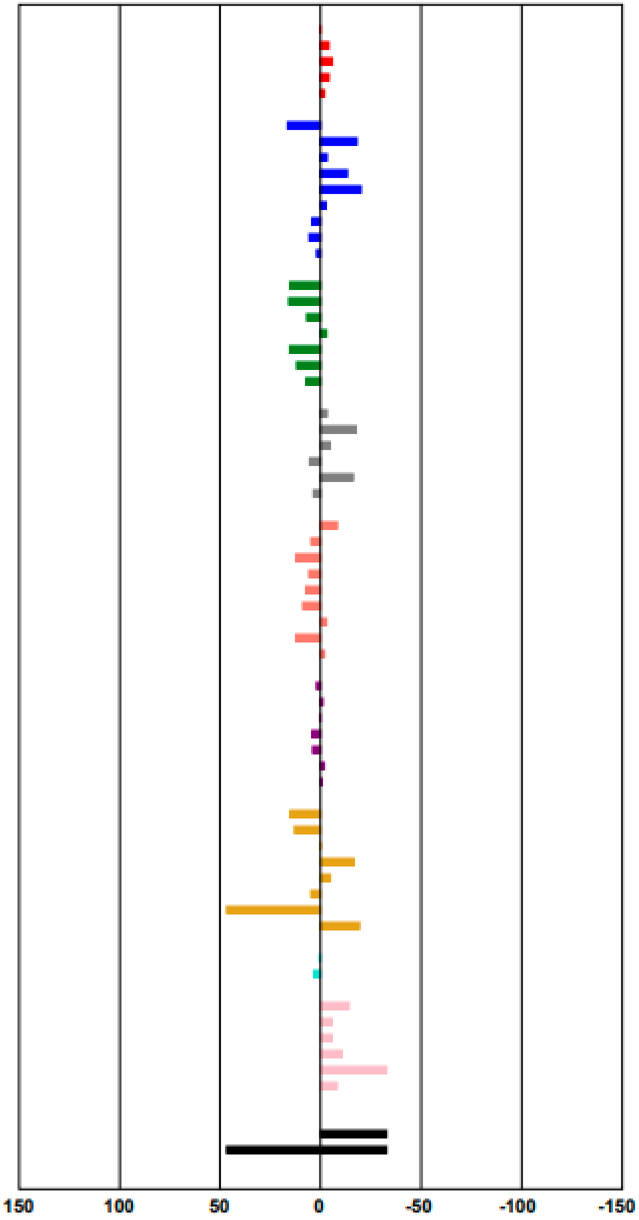

Developmental Therapeutics Program	NSC: D-837132 / 1	Conc: 1.00E-5 Molar	Test Date: Mar 28, 2022
One Dose Mean Graph	Experiment ID: 2203OS96		Report Date: Apr 16, 2022

Panel/Cell Line	Growth Percent
**Leukemia**	
CCRF-CEM	88.33
HL-60(TB)	83.99
K-562	82.28
MOLT-4	83.93
RPMI-8226	86.16
**Non-Small Cell Lung Cancer**	
A549/ATCC	104.40
EKVX	70.01
HOP-62	84.85
HOP-92	74.75
NCI-H226	67.97
NCI-H23	85.44
NCI-H322M	92.24
NCI-H460	93.65
NCI-H522	89.98
**Colon Cancer**	
COLO 205	103.28
HCC-2998	103.78
HCT-116	94.85
HCT-15	85.14
HT29	103.33
KM12	99.84
SW-620	95.21
**CNS Cancer**	
SF-268	84.83
SF-295	70.43
SF-539	83.34
SNB-19	93.43
SNB-75	71.80
U251	91.53
**Melanoma**	
LOX IMVI	79.74
MALME-3M	92.72
M14	100.27
MDA-MB-435	93.79
SK-MEL-2	95.34
SK-MEL-28	96.82
SK-MEL-5	85.13
UACC-257	100.23
UACC-62	86.16
**Ovarian Cancer**	
IGROV1	90.04
OVCAR-3	86.93
OVCAR-4	88.39
OVCAR-5	92.23
OVCAR-8	91.94
NCI/ADR-RES	86.24
SK-OV-3	87.37
**Renal Cancer**	
786-0	103.02
A498	100.99
ACHN	88.14
CAKI-1	71.59
RXF 393	83.22
SN12C	92.76
TK-10	134.72
UO-31	68.72
**Prostate Cancer**	
PC-3	88.55
DU-145	91.26
**Breast Cancer**	
MCF7	73.92
MDA-MB-231/ATCC	82.40
HS 578T	82.43
BT-549	77.56
T-47D	55.29
MDA-MB-468	79.85
Mean	88.08
Delta	32.79
Range	79.43

Figure S59. One dose mean graph for compound **11b** at 10 μM

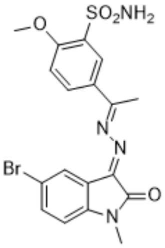

11c

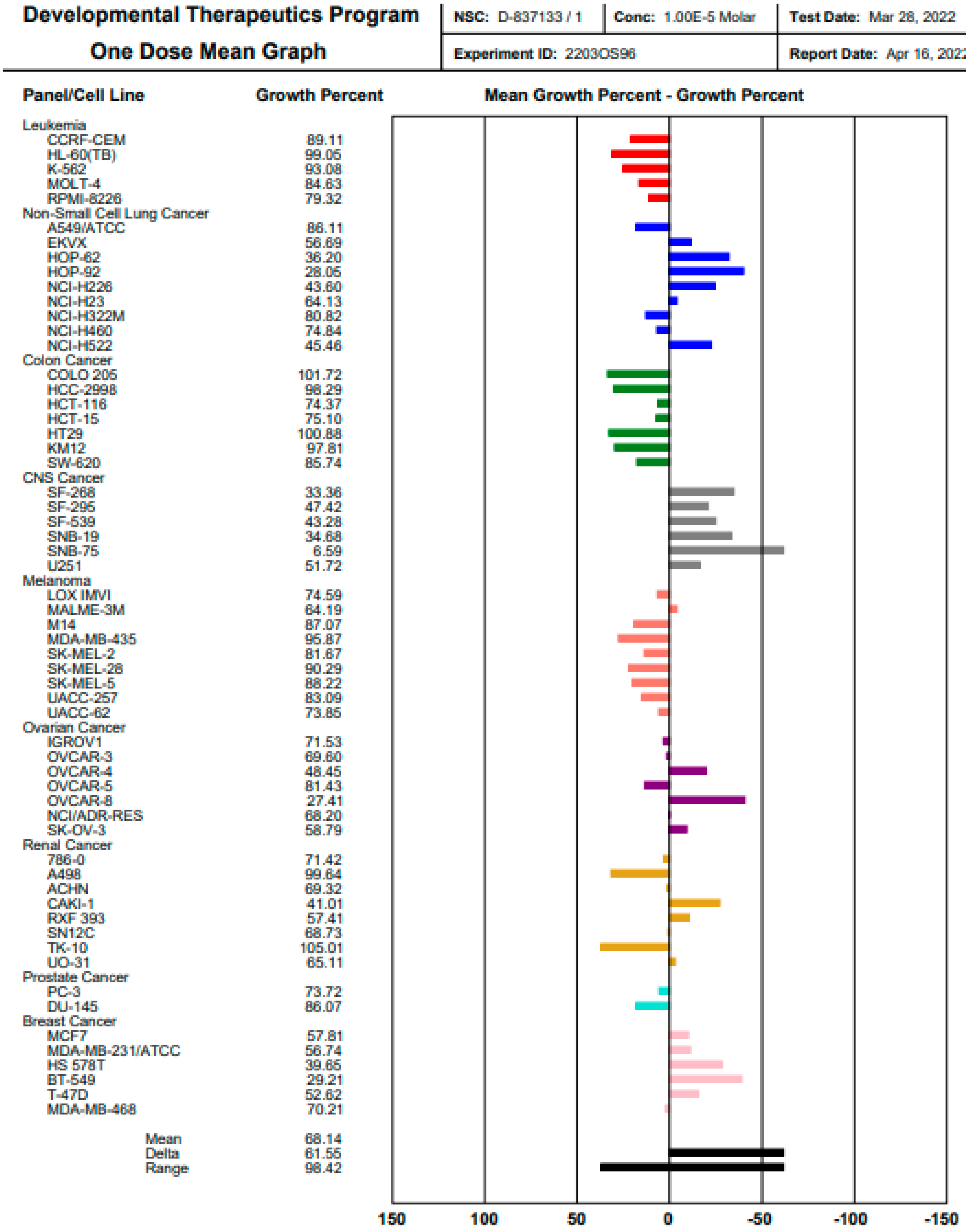

Figure S60. One dose mean graph for compound **11c** at 10 μM

Developmental Therapeutics Program | **NSC:** D-837134 / 1 | **Conc:** 1.00E-5 Molar | **Test Date:** Mar 28, 2022

One Dose Mean Graph | **Experiment ID:** 2203OS96 | **Report Date:** Apr 16, 202[illegible]

Panel/Cell Line	Growth Percent
Leukemia	
HL-60(TB)	93.94
K-562	67.22
MOLT-4	70.07
RPMI-8226	79.47
Non-Small Cell Lung Cancer	
A549/ATCC	82.80
EKVX	78.09
HOP-62	122.07
HOP-92	86.49
NCI-H226	74.58
NCI-H23	83.61
NCI-H322M	92.34
NCI-H460	85.57
NCI-H522	75.45
Colon Cancer	
COLO 205	130.88
HCC-2998	103.90
HCT-116	77.37
HCT-15	82.56
HT29	109.07
KM12	97.55
SW-620	99.10
CNS Cancer	
SF-268	82.38
SF-295	76.25
SF-539	91.36
SNB-19	80.04
SNB-75	73.83
U251	90.49
Melanoma	
LOX IMVI	88.34
MALME-3M	93.37
M14	94.48
MDA-MB-435	92.85
SK-MEL-2	93.36
SK-MEL-28	90.24
SK-MEL-5	80.54
UACC-257	87.07
UACC-62	78.93
Ovarian Cancer	
IGROV1	99.19
OVCAR-3	82.07
OVCAR-4	81.55
OVCAR-5	89.90
OVCAR-8	91.58
NCI/ADR-RES	86.40
SK-OV-3	112.36
Renal Cancer	
786-0	93.27
A498	115.76
ACHN	90.89
CAKI-1	81.20
RXF 393	77.54
SN12C	81.68
TK-10	114.39
UO-31	80.11
Prostate Cancer	
PC-3	81.38
DU-145	96.25
Breast Cancer	
MCF7	81.04
MDA-MB-231/ATCC	76.96
HS 578T	78.49
BT-549	67.52
T-47D	57.06
MDA-MB-468	56.44
Mean	87.22
Delta	30.78
Range	74.44

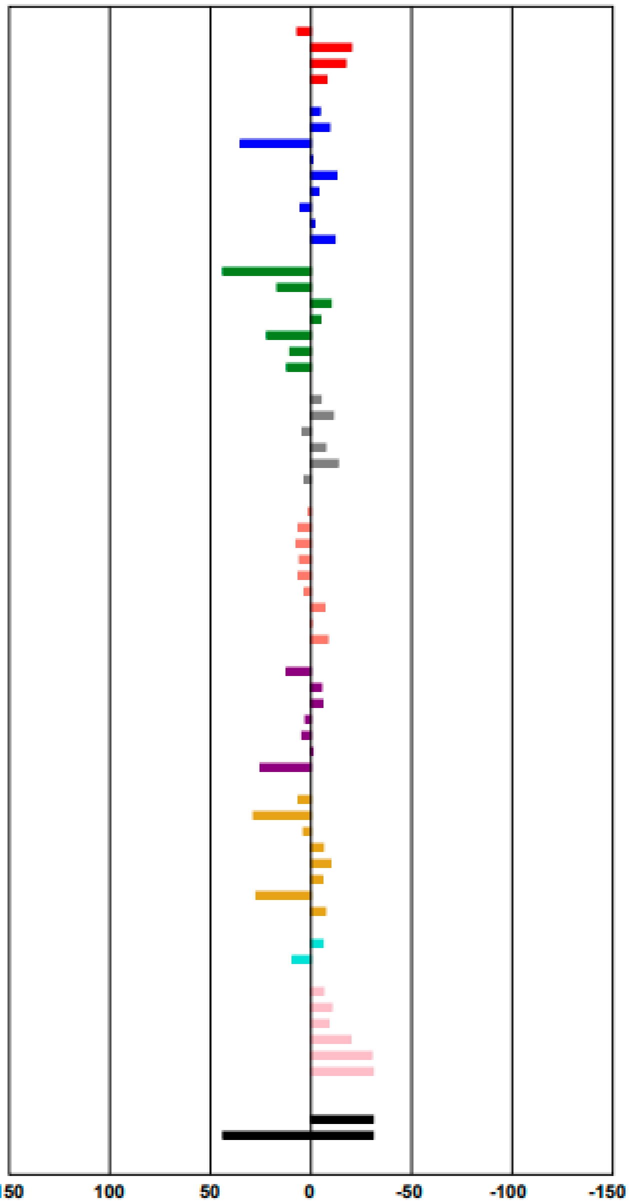

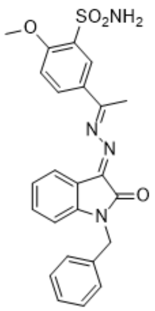

12a

Figure S61. One dose mean graph for compound **12a** at 10 μM

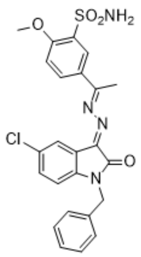

12b

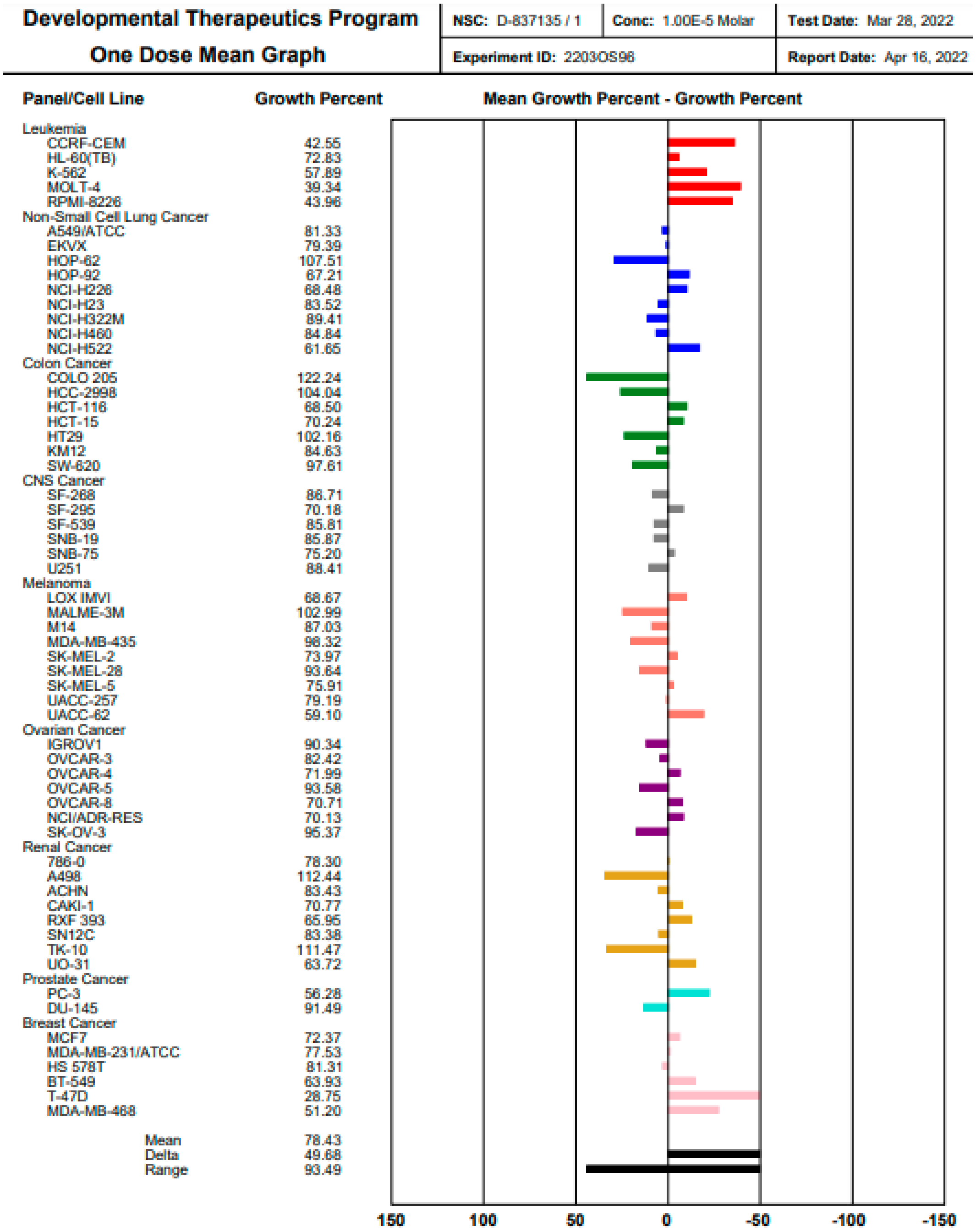

Figure S62. One dose mean graph for compound **12b** at 10 μM

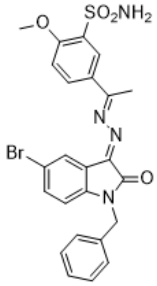

12c

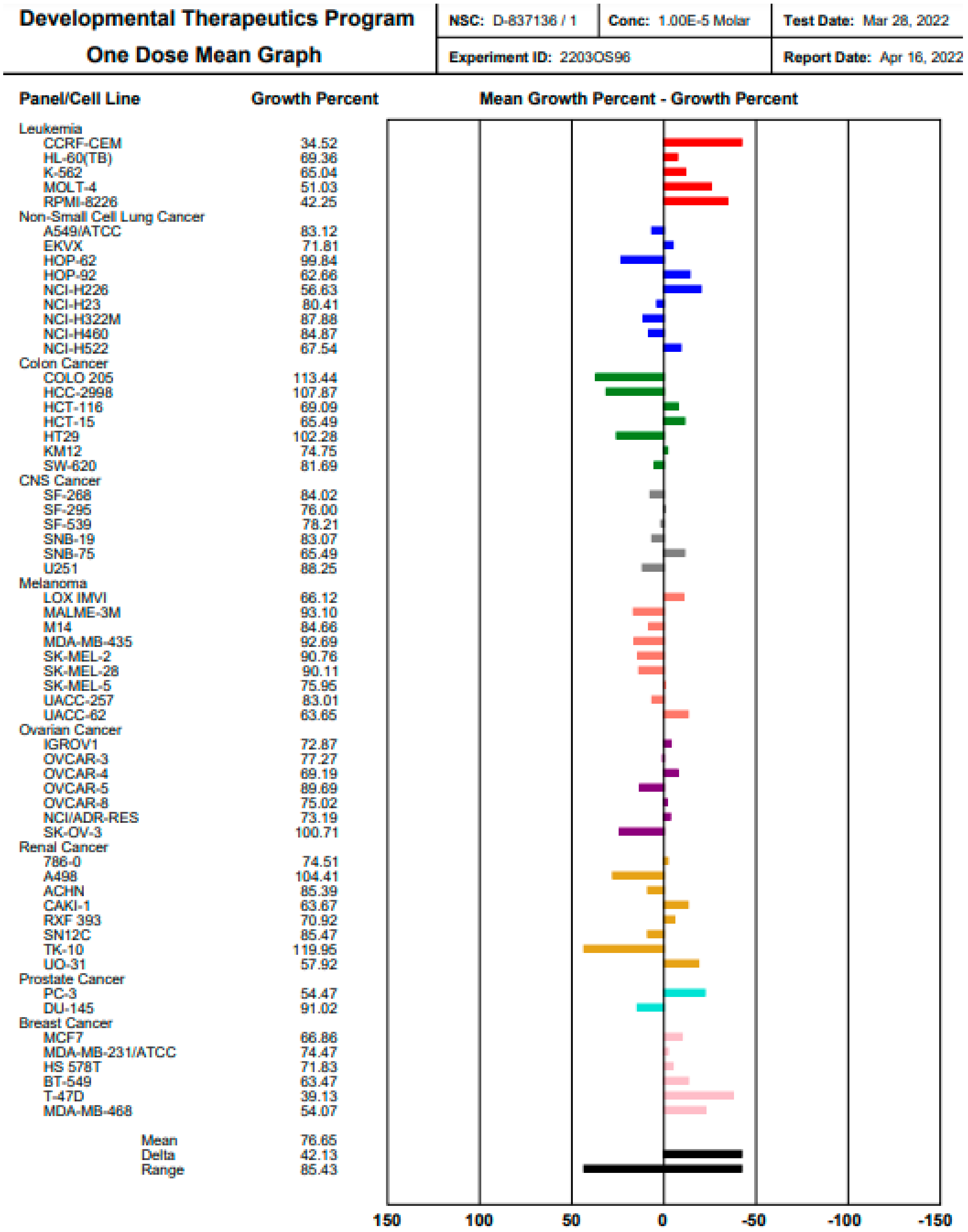

Figure S63. One dose mean graph for compound **12c** at 10 μM

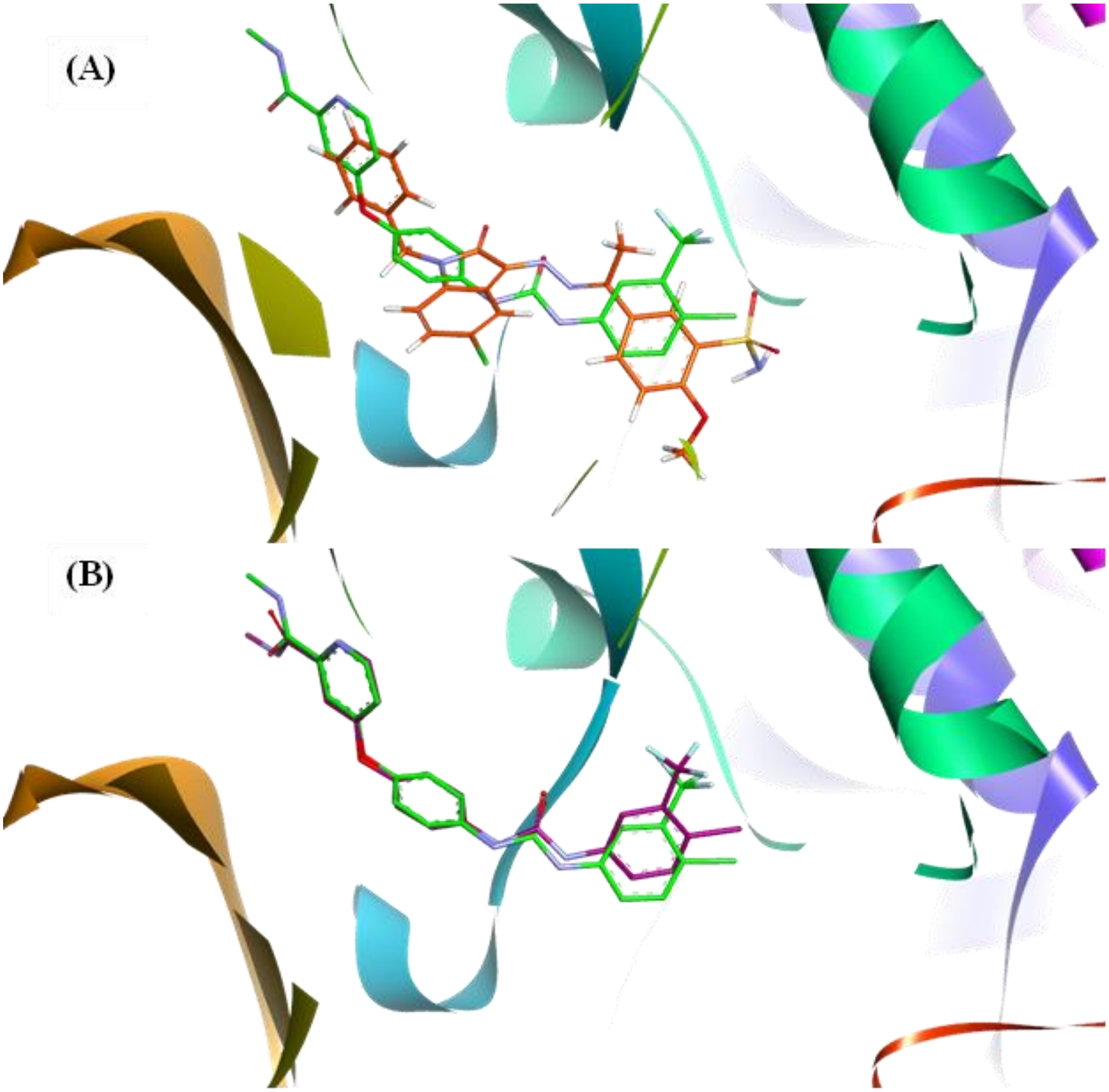

Figure S64. Overlay of (A) **12b** and (B) docked sorafenib (RMSD= 1.005Å) with the co-crystalized sorafenib inside the active site of VEGFR-2 receptor (code: 4ASD).

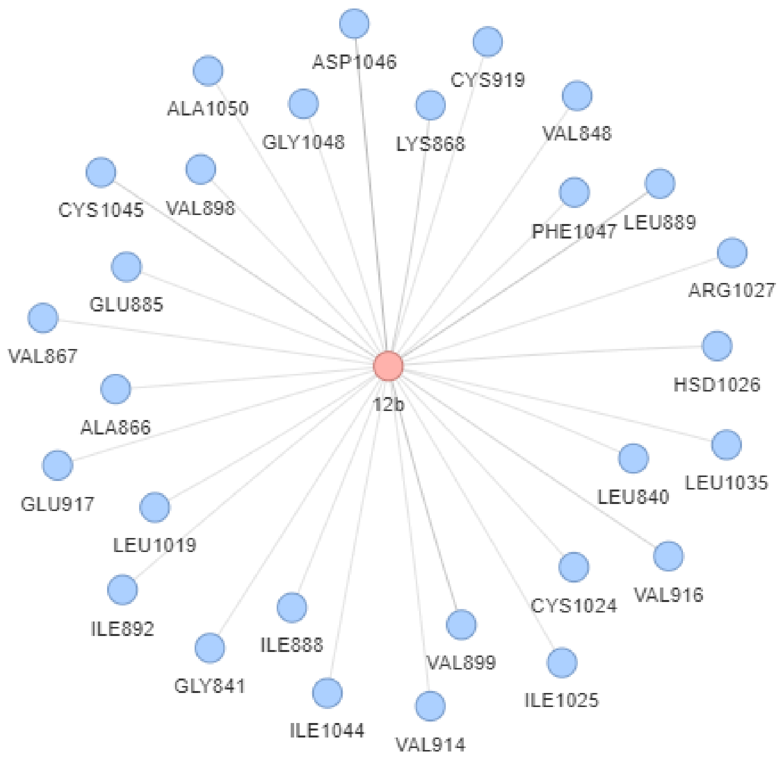

Figure S65. Hydrophobic interactions of **12b** inside the active site of VEGFR-2 receptor.

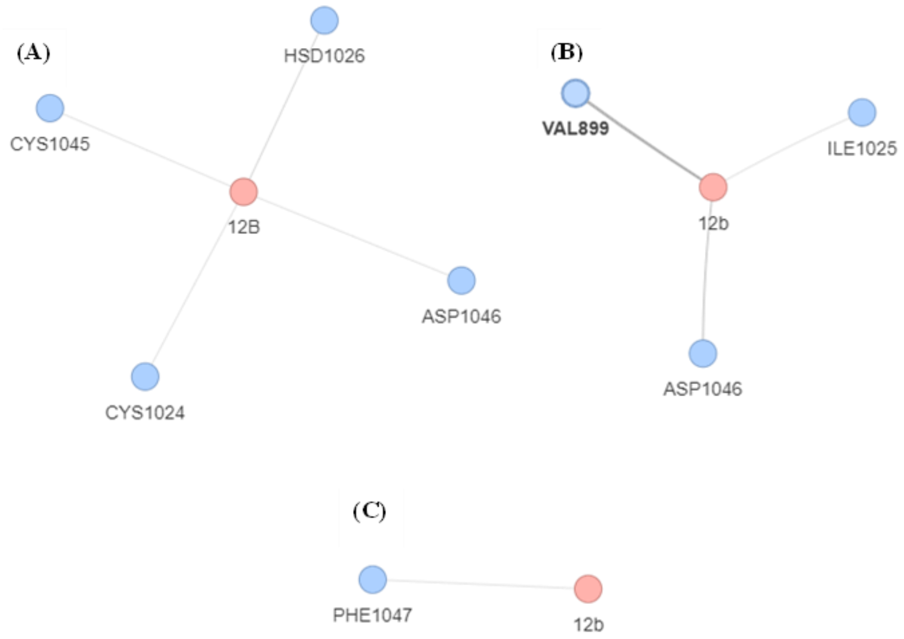

Figure S66. Interactions of **12b** inside the active site of VEGFR-2 receptor (A) H-Bond acceptor, (B) H-Bond donor and (C) π-stacking.

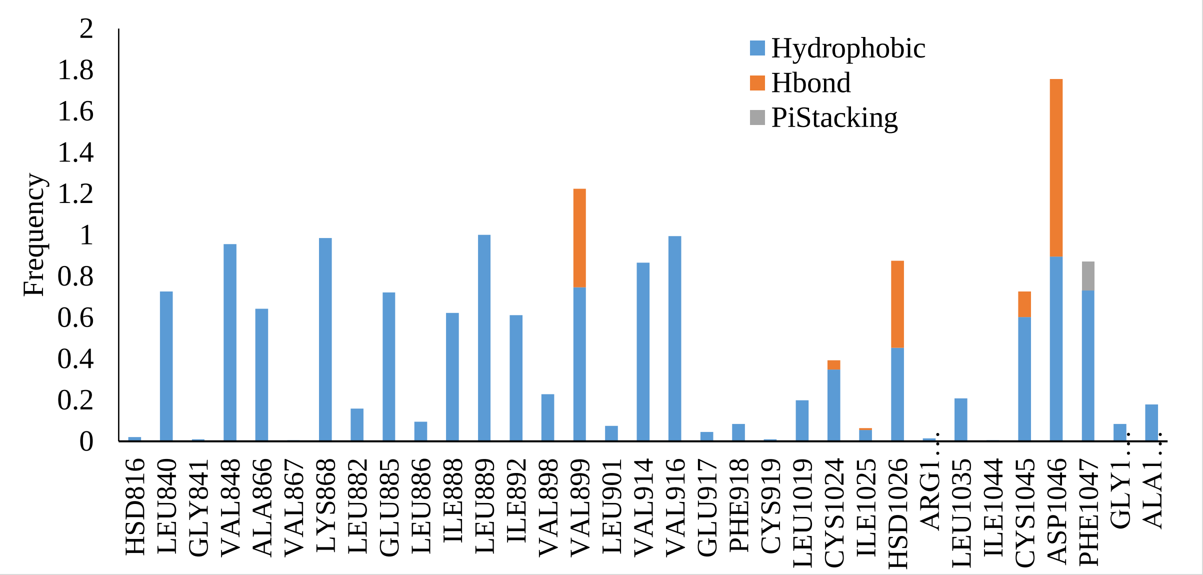

Figure S67. Contribution (frequency and type of interaction) of each amino acid inside the active site of VEGFR-2 receptor (code: 4ASD) in the binding affinity of **12b**.

Biological evaluation

NCI-60 cancer cell lines screening

Primary anticancer assay was performed on a panel of sixty human tumour cell lines derived from nine neoplastic diseases, in accordance with the protocol of the Drug Evaluation Branch, National Cancer Institute, Bethesda, Maryland, USA. Tested compounds were added to the cell culture at a single concentration (10 mM) and the cultures were incubated for 48 h. End point determinations were made with a protein binding dye, sulforhodamine B (SRB). Results for each tested compound were reported as the percent of growth of treated cells when compared to untreated control cells. The percentage growth was evaluated spectrophotometrically versus controls not treated with test agents [1].

In vitro cytotoxicity

The cytotoxic efficacy of the target compounds against T47D cells was determined, using the SRB colorimetric technique, as previously reported. Briefly, exponentially growing cells were trypsinized, counted and seeded at the appropriate densities (5000 cells/100 µL/ well) into 96-well microtiter plates. Cells were incubated in a humidified atmosphere at 37 °C for 24 h. Then, the cells were exposed to the tested compounds at the desired concentrations (0.01, 0.1, 1, 10, and 100 µM) or to 1% dimethyl sulfoxide (DMSO) for 72 h. At the end of the treatment period, the media were removed, and the cells were fixed with 10% trichloroacetic acid at 4 °C for 1 h. Following, the cells were washed with tap water four times and incubated with SRB 0.4% for 30 min. Excess dye was removed by washing repeatedly with 1% (vol/vol) acetic acid. The protein-bound dye was dissolved in 10 mM Tris base solution for (optical density) OD determination at 510 nm using a Spectra Max plus Microplate Reader (Molecular Devices, CA). Cell viability was expressed relative to the untreated control cells [2, 3].

VEGFR-2 inhibition assay

This assay was performed according to the reported method [4, 5]. HTRF (homogeneous time-resolved fluorescence) assay method was used to conduct inhibitory action of all newly synthesized compounds against VEGFR-2. VEGFR-2 kinase (BioAssay Systems - Kinase Assay Kit, EKIN-400) and its substrate were incubated for 5 min in a buffer solution with newly synthesized compounds/Sorafnib to initiate the enzymatic reaction, then ATP was added to the reaction mixture. Hold under 30 min at room temperature. The reaction was stopped by adding detection reagents containing EDTA for 1 h then the IC_{50} values have been determined.

Carbonic anhydrase I, II, and IX inhibition studies

The CO_2 hydration actions catalyzed by carbonic anhydrase for targeted compounds herein presented were measured using an Applied Photophysics stopped-flow instrument. The enzymes are recombinant proteins that have been created in a lab. Following the initial rates of the CA-catalyzed CO_2 hydration reaction for a period of 10-100 s, phenol red (at a concentration of 0.2 mM) was used as an indicator, working at an absorbance maximum of 557 nm, with 20 mM Hepes (pH 7.5) as a buffer, and 20 mM Na_2SO_4 (to maintain constant the ionic strength). The CO2 concentrations used to determine the kinetic parameters and inhibition constants ranged from 1.7 to 17 mM. At least six traces of the first 5-10% of the reaction were utilized to determine the beginning velocity for each inhibitor. In the same way, the uncatalyzed rates were calculated and deducted from the total measured rates. Stock inhibitor solutions (0.1 mM) were produced in distilled-deionized water, followed by dilutions with the assay buffer up to 0.01 nM. Prior to the experiment, the inhibitor and enzyme solutions were preincubated together for 15 minutes at room temperature to allow for the development of the E-I complex. The inhibition constants were calculated using non-linear least-squares methods using the ChengPrusoff equation in PRISM 3. They reflect the average of at least three individual measurements [6, 7].

Cell cycle analysis

T47D cells were seeded at concentrations of 1 x 10^5 cells per well in a 6-well plate, and then incubated for 24 h. The cells were treated for 24 h with vehicles (0.1 percent DMSO) or 10 μM of compounds **11b** and **12b**. Using ice-cold, 70 percent ethanol at 4 °C, cells were harvested and fixed for 12 h. Ethanol was removed, and the cells were washed by cold PBS. Then, the cells were incubated in 0.5 mL of PBS containing 1 mg/mL Ranse for 30 min at 37 °C. In the dark, the cells were stained with propidium iodide for 30 min. Cell cycle analysis and apoptosis were conducted using a FACS Calibur flow cytometer as shown in previous studies [8, 9]. Every experiment has been carried out three times.

Annexin V-FITC apoptosis assay

Cells were incubated with the tested compounds **11b** and **12b** for 24h, trypsinized and washed with cold phosphate-buffer saline (PBS). Cells were then stained with Annexin V-FITC and PI in binding buffer for 15 min at room temperature in the dark. The samples were analyzed using the flow cytometer [8, 9].

MD simulation

GROMACS 2021 was used to run molecular dynamics (MD) simulations on the best docking poses [10]. The CHARMM General Force Field (CGenFF) server was utilized to generate the ligands topology, whereas the input files for the MD calculations were created applying the CHARMM force field parameters for protein [11]. They were immersed in a TIP3P water model cubic box with 10 padding and neutralized by adding Na^+ and Cl^- ions. For long-range electrostatic interactions and Non-bonded interactions were treated with a 12 ˚A cutoff distance and the neighbor searching list were buffered with the Verlet cutoff-scheme [12] and the long-range electrostatic interactions were treated with the particle mesh Ewald (PME) method [13]. CHARMM36 forcefield was applied on the protein-ligand complex [14]. Prior to production simulation, energy minimization of the system was carried out by using steepest descent algorithm (5000 steps). The complex was then equilibrated for stabilizing its temperature and pressure by subjecting it to NVT and NPT ensemble and simulating for 125 ps at 300.15 K temperature using 400 kJ mol^{-1} nm^{-2} and 40 kJ mol^{-1} nm^{-2} positional restraints on the backbone and side chains, respectively. Finally, the complex is subjected to production simulation run for 100 ns in NPT ensemble at 300.15 K and 1 bar. To maintain the temperature Nose-Hoover thermostat was used [15] and similarly for maintaining the pressure Parrinello-Rahman barostat was used [16]. LINCS algorithm was used for constraining H-bonds [17]. The V-rescale thermostat at 300 K with a coupling constant of 1 ps was used. The trajectories were stored every 2 ps. Simulations of 100 ns in NPT assembly were performed for the production stage. GROMACS utilities were used for the analysis of the MD simulations [18]. The root mean square deviation (RMSD) of atom position for ligand and protein was calculated by fitting protein backbone atom with the *gmx_rms* subprogram. Similarly, root mean square fluctuations (RMSF) based on the protein C-alpha atoms were calculated using *gmx_rmsf*. Radius of gyration of all protein atoms was calculated with the *gmx_gyrate* and number of hydrogen bonds were calculated (in-side the protein-ligand interface) with the *gmx_hbond*. The utility *gmx_distance* was used to calculate the center of mass distance between the protein and the ligand during the simulation. The VMD molecular graphics program was used for trajectory visualization and protein-ligand contact frequency analysis. For systems which were chosen for further analysis, MM/PBSA (Molecular Mechanics/Poisson−Boltzmann Surface Area) calculations were done using *g_mmpbsa*, a GROMACS tool used to calculate an estimated binding affinity [19].

References

[1] M. H. Saad, T. F. El-Moselhy, E.-D. S, Nabaweya, A. B. Mehany, A. Belal, M. A. Abourehab, H. O. Tawfik and M. H. El-Hamamsy. Discovery of new symmetrical and asymmetrical nitrile-containing 1, 4-dihydropyridine derivatives as dual kinases and P-glycoprotein inhibitors: synthesis, in vitro assays, and in silico studies. *Journal of Enzyme Inhibition and Medicinal Chemistry.* 37 (2022) 2489-2511.

[2] A. Hassan, M. Badr, D. Abdelhamid, H. A. Hassan, M. A. Abourehab and G. E. D. A. Abuo-Rahma. Design, synthesis, in vitro antiproliferative evaluation and in silico studies of new VEGFR-2 inhibitors based on 4-piperazinylquinolin-2 (1H)-one scaffold. *Bioorganic Chemistry.* 120 (2022) 105631.

[3] A. Hassan, M. Badr, H. A. Hassan, D. Abdelhamid and G. E. D. A. Abuo-Rahma. Novel 4-(piperazin-1-yl) quinolin-2 (1H)-one bearing thiazoles with antiproliferative activity through VEGFR-2-TK inhibition. *Bioorganic & medicinal chemistry.* 40 (2021) 116168.

[4] M. M. Al-Sanea, A. Hamdi, A. A. Mohamed, H. W. El-Shafey, M. Moustafa, A. A. Elgazar, W. M. Eldehna, H. Ur Rahman, D. G. Parambi and R. M. Elbargisy. New benzothiazole hybrids as potential VEGFR-2 inhibitors: design, synthesis, anticancer evaluation, and in silico study. *Journal of Enzyme Inhibition and Medicinal Chemistry.* 38 (2023) 2166036.

[5] S. S. Zahran, F. A. Ragab, M. G. El-Gazzar, A. M. Soliman, W. R. Mahmoud and M. M. Ghorab. Antiproliferative, antiangiogenic and apoptotic effect of new hybrids of quinazoline-4 (3H)-ones and sulfachloropyridazine. *European Journal of Medicinal Chemistry.* 245 (2023) 114912.

[6] H. O. Tawfik, A. Petreni, C. T. Supuran and M. H. El-Hamamsy. Discovery of new carbonic anhydrase IX inhibitors as anticancer agents by toning the hydrophobic and hydrophilic rims of the active site to encounter the dual-tail approach. *European Journal of Medicinal Chemistry.* 232 (2022) 1-21.

[7] A. I. Zain-Alabdeen, T. F. El-Moselhy, N. Sharafeldin, A. Angeli, C. T. Supuran and M. H. El-Hamamsy. Synthesis and anticancer activity of new benzensulfonamides incorporating s-triazines as cyclic linkers for inhibition of carbonic anhydrase IX. *Scientific reports.* 12 (2022) 16756.

[8] R. Chen, Z. Wang, L. Sima, H. Cheng, B. Luo, J. Wang, B. Guo, S. Mao, Z. Zhou and J. Peng. Design, synthesis and evaluation of 2, 6, 8-substituted Imidazopyridine derivatives as potent PI3K α inhibitors. *Journal of Enzyme Inhibition and Medicinal Chemistry.* 38 (2023) 2155638.

[9] H. Kaur, A. Singh, K. Kaur, A. Kumar, S. Attri, F. Rashid, S. Singh, N. Bedi, H. S. Tuli and S. Haque. 4-methylthiobutyl isothiocyanate synergize the antiproliferative and pro-apoptotic effects of paclitaxel in human breast cancer cells. *Biotechnology and Genetic Engineering Reviews.* (2023) 1-25.

[10] M. J. Abraham, T. Murtola, R. Schulz, S. Páll, J. C. Smith, B. Hess and E. Lindahl. GROMACS: High performance molecular simulations through multi-level parallelism from laptops to supercomputers. *SoftwareX.* 1-2 (2015) 19-25.

[11] K. Vanommeslaeghe, E. Hatcher, C. Acharya, S. Kundu, S. Zhong, J. Shim, E. Darian, O. Guvench, P. Lopes, I. Vorobyov and A. D. Mackerell, Jr. CHARMM general force field: A force field for drug-like molecules compatible with the CHARMM all-atom additive biological force fields. *J Comput Chem.* 31 (2010) 671-690.

[12] T. F. D. Silva, D. Vila-Viçosa, P. Reis, B. L. Victor, M. Diem, C. Oostenbrink and M. Machuqueiro. The Impact of Using Single Atomistic Long-Range Cutoff Schemes with the GROMOS 54A7 Force Field. *J Chem Theory Comput.* 14 (2018) 5823-5833.

[13] T. Nozawa, K. Yasuoka and K. Z. Takahashi. Critical test of isotropic periodic sum techniques with group-based cut-off schemes. *Sci Rep.* 8 (2018) 4185.
[14] A. Croitoru, S. J. Park, A. Kumar, J. Lee, W. Im, A. D. MacKerell, Jr. and A. Aleksandrov. Additive CHARMM36 Force Field for Nonstandard Amino Acids. *J Chem Theory Comput.* 17 (2021) 3554-3570.
[15] D. J. Evans and B. L. Holian. The Nose–Hoover thermostat. 83 (1985) 4069-4074.
[16] R. Martonák, A. Laio and M. Parrinello. Predicting crystal structures: the Parrinello-Rahman method revisited. *Phys Rev Lett.* 90 (2003) 075503.
[17] B. Hess, H. Bekker, H. J. C. Berendsen and J. G. E. M. Fraaije. LINCS: A linear constraint solver for molecular simulations. 18 (1997) 1463-1472.
[18] D. Van Der Spoel, E. Lindahl, B. Hess, G. Groenhof, A. E. Mark and H. J. C. Berendsen. GROMACS: Fast, flexible, and free. 26 (2005) 1701-1718.
[19] N. Homeyer and H. Gohlke. Free Energy Calculations by the Molecular Mechanics Poisson-Boltzmann Surface Area Method. *Mol Inform.* 31 (2012) 114-122.
